# Mobility-state- and handover-direction-aware analytical and machine learning-based HCP optimization in 5G heterogeneous networks

**DOI:** 10.1371/journal.pone.0362310

**Published:** 2026-11-12

**Authors:** Fozia Panhwer, Muhammad Mujtaba Shaikh, Beenish Ansari, Iqra Ameer, Mohamed A. Elmagzoub, Asadullah Shaikh

**Affiliations:** 1 Department of Telecommunication Engineering, Quaid-e-Awam University of Engineering, Science & Technology, Nawabshah, Sindh, Pakistan; 2 Department of Electronics Engineering, University of Sindh, Jamshoro, Pakistan; 3 Division of Science, Engineering, and Technology, The Pennsylvania State University, Abington, Pennsylvania, United States of America; 4 Department of Network and Communication Engineering, College of Computer Science and Information Systems, Najran University, Najran, Saudi Arabia; 5 Emerging Technologies Research Lab (ETRL), College of Computer Science and Information Systems, Najran University, Najran, Saudi Arabia; 6 Department of Information Systems, College of Computer Science and Information Systems, Najran University, Najran, Saudi Arabia; Dr Shakuntala Misra National Rehabilitation University, INDIA

## Abstract

The use of ultra-dense small cells in 5G heterogeneous networks increases capacity, coverage and spectral efficiency, but it also complicates mobility management. In dense macro-small-cell networks, user devices often cross cells with varying coverage, transmit power, and propagation conditions, which can lead to high risks of radio-link failure, handover failure, ping-pong handover and unnecessary handover. These problems are exacerbated by using fixed handover-control settings, in which a single-value handover margin and time-to-trigger cannot provide a good trade-off across UE velocities and handover directions. In this paper, we propose a mobility-state- and handover-direction-aware HCP optimization for 5G HetNets while retaining the Event A3 handover scheme, state-based handover flow and radio-link-monitoring framework. The proposed framework is simulated with 3D geometry with user height, 3GPP TR 38.901 UMa/UMi propagation models, and a repeatable two-tier macro-small-cell deployment. Four controllers are compared with the same settings: fixed A3, an analytical adaptive controller, tabular Q-learning, and an improved deep Q-network (DQN). The analytical adaptive controller optimizes HOM and TTT based on the UE velocity and handover direction (macro to small, small to macro, and same-tier handovers), and the learning-based controllers use a multi-KPI reward to learn discrete HOM-TTT actions. Across the tested UE velocities, tabular Q-learning improves the principal mobility-robustness KPIs relative to fixed A3, while DQN provides the best observed mean multi-KPI balance among the four controllers. The DQN trend is also consistent with recent ML/DRL handover-optimization studies while retaining the conventional Event A3, finite-state handover, and radio-link-monitoring framework. Because the policy-level comparison is based on three evaluation runs, the reported uncertainty measures are interpreted descriptively and the conclusions are limited to the tested simulation conditions.

## Introduction

Cellular systems have always been subject to a trade-off between stability and agility [1–3]. This issue has recently become more critical because the rapid growth of mobile users and data-driven services is increasing traffic demand. Worldwide mobile data traffic grew to around 130 Exabytes (EBs) per month in 2023 and is expected to grow rapidly towards the end of the decade [4]. These forecasts indicate that reliable mobility management must operate under ever-growing traffic loads. In fifth-generation (5G) systems, these requirements are addressed through heterogeneous network (HetNet) topologies comprising macro cells and lower-power small cells. Small cells augment capacity and frequency reuse [5–12]. Although this improves spectral efficiency, it also poses considerable challenges for mobility management. In this scenario, user equipment (UE) frequently moves among macro and small cells with different coverage ranges, transmit powers, and propagation conditions. This makes handover highly dynamic and dependent on user speed, cell-size asymmetry, inter-site distance, target-cell residence time, and handover direction [13–15].

In terms of the standards, mobility decisions in 5G are based on measurement-based procedures specified in 3GPP TS 38.331. In particular, Event A3 is often used for handover triggering: a handover is triggered when the target-cell signal is stronger than the serving-cell signal by a specified handover margin (HOM) for a specified time-to-trigger (TTT) interval [16–18]. The same specification describes radio-link monitoring (RLM) procedures through timers and counters such as T310, T311, N310, and N311, which are used to identify radio-link failure (RLF). These mechanisms show that handover optimization and link reliability are closely related because late or incorrect handover decisions can cause handover failure (HOF) or service interruption [19–21].

The propagation environment also affects handover. Realistic evaluation must use channel models (such as those in 3GPP TR 38.901, which define urban micro (UMi) scenarios with different heights of the base stations, carrier frequencies, and line-of-sight (LOS) and non-line-of-sight (NLOS) propagation) [22–24]. In heterogeneous networks, macro cells generally operate at lower frequencies with higher antennas and small cells operate at higher frequencies with lower coverage. These distinctions result in an asymmetry in signal strengths, which directly affect handover triggering and decisions [25]. Another reference is 3GPP TR 36.839, which offers a comprehensive framework for mobility robustness measurement, and clarifies interpretation of HOF, ping-pong and RLF events [26].

Within this framework, HOM and TTT are still the most important handover control parameters (HCPs). Low HOM and short TTT lead to early handover but can cause ping-pong effects and HOF; high HOM and TTT values lead to higher stability but may cause late handovers, HOF and RLF [27]. Thus, the key optimization problem is not if HOM and TTT are important, but how they should be adapted. From an optimization point of view, HOM and TTT are suitable optimization variables because they are understandable and actionable. Unlike many high-dimensional or black-box policies, these parameters are already part of standard handover protocols and have physical interpretations. HOM is a measure of confidence: how much stronger the target must be for switching to be beneficial. TTT represents temporal stability: duration of the superiority of the target before the trigger is followed. A mobility-state-aware analytical formulation can thus be chosen to reflect some intuitive rules, such as setting low TTT for fast-changing trajectories or high HOM in cases where small fluctuations would result in oscillatory handovers [28,29]. There are also clear reasons for developing an analytical formulation before relying on complete machine-learning optimization. Analytical models are easier to validate, parameterize, and interpret for network analysis. They do not require large training or exploration sets, and they are less dependent on reward design and convergence behaviour than reinforcement-learning methods. Moreover, an analytical baseline provides a strong benchmark: if mobility-state-aware analytical adaptation already produces meaningful gains, then learning-based approaches must outperform a strong and interpretable baseline rather than simply exceed a weak fixed-threshold baseline [30].

Recently, both analytical and learning-based methods have been investigated to optimize HCP. Analytical and rule-based methods typically make use of mobility information, such as UE speed, to dynamically update HOM and TTT while maintaining interpretability and efficiency [31]. Reinforcement-learning algorithms, such as Q-learning and deep Q-network (DQN), have also been applied to adaptive handover [32–36]. However, many previous ML-based studies have key limitations. First, some works develop handover with signal strength or mobility status, but do not explicitly consider handover direction, which can be more or less risky (macro-to-small vs. small-to-macro). Second, some research uses an ideal deployment or propagation model, rather than 3D with height-aware propagation geometry and 3GPP TR 38.901 UMa/UMi. Third, some learning-based methods deviate from the standard handover procedure, which makes it hard to compare them to Event A3-based methods. Finally, many works do not consider the set of KPIs together, such as throughput, latency, handover rate, ping-pong, HOF, RLF, successful handover (SHO), and outage [37–43].

Recent learning-based and HetNet mobility studies can be distinguished from the present framework in terms of mobility-state representation, handover-direction awareness, propagation modelling, and standards compatibility [44,45].

Lv et al. emphasize the role of deep learning in intelligent 5G network management, including security and resource-control problems that motivate data-driven network optimization [46]. Kiran presents vertical handover self-optimization in 5G HetNets, which is directly related to mobility management but does not provide the same joint mobility-state- and handover-direction-aware comparison under a common Event A3/FSM/RLM framework [47]. Alhulayil and López-Benítez show that coexistence mechanisms such as almost blank subframes can affect SINR and resource availability in heterogeneous deployments, which further motivates the use of SINR-aware and direction-aware HCP decisions [48].

Although these studies have progressed, the majority are still focused on mobility-state-aware optimization, and do not explicitly consider handover direction. In heterogeneous networks, handovers are asymmetrical. For instance, handovers from macro to small cells may involve short and variable residence times, and small-to-macro transitions might need more frequent triggering to prevent late handover and radio link failure. Ignoring this can have a negative impact on the performance of both model-based and data-driven solutions [49,50].

As shown in Table 1, the novelty does not lie only in the use of DQN. The key difference is that the control state explicitly represents the handover direction and combines it directly with the mobility state, rather than deriving the decision only from instantaneous signal measurements. The direction-resolved state covers macro-to-small, small-to-macro, and same-tier transitions. It is used consistently by the analytical and learning-based controllers, while the standard Event A3 trigger, FSM, and RLM procedures remain unchanged.

**Table 1:** Comparison with recent learning-based handover-optimization studies.

Study	Learning/control approach	Principal state or inputs	Mobility state explicit	HO direction explicit	Main distinction from the present work
He et al. (2023) [23]	DQN with LSTM-aided digital twin	Signal-fading condition and predicted environment state	Partial	No	Optimizes handover parameters through a digital twin, but does not jointly encode macro-to-small, small-to-macro, and same-tier direction within a common Event A3/FSM/RLM comparison.
Kwong et al. (2024) [49]	DDPG-based handover-parameter optimization	Mobility condition and radio measurements; continuous HOM control	Yes	No	Learns continuous HOM adaptation in a UDN; the present work jointly optimizes HOM–TTT and makes tier-transition direction an explicit state variable.
Alhammadi et al. (2024) [41]	Self-optimizing MRO/LBO control	Link reliability, mobility and load-related indicators	Yes	Implicit	Addresses resilient mobility and load balancing, whereas the present work isolates direction-specific HCP control and compares analytical, tabular RL and DQN policies under identical 3GPP assumptions.
Riaz et al. (2025) [51]	Data-driven MLP prediction of HOM and TTT	Speed, RSRP, SINR and cell load	Yes	No	Predicts both HCPs from an optimized dataset; the present work adds explicit handover direction, Event A3/FSM/RLM preservation and a four-controller common benchmark.
Kumar et al. (2025) [50]	DDPG actor–critic handover optimization	RSRP, SINR and mobility/network conditions	Yes	No	Uses continuous DRL adaptation; the present work uses a constrained standards-compatible HOM–TTT action set and direction-consistency reward.
**Present work**	Analytical adaptive control, Q-learning and DQN	Speed, signal gap, SINR, time since HO, signal-gap derivative, serving tier and explicit HO direction	**Yes**	**Yes**	Joint mobility-state- and direction-aware HOM–TTT optimization with fixed A3, analytical, Q-learning and DQN evaluated under the same 3D topology, TR 38.901 propagation, FSM/RLM logic and KPI definitions.

### Research gap

The main issue is the lack of a standards-compatible, unified HCP optimization method that jointly accounts for UE mobility state and handover direction while retaining the Event A3/FSM/RLM handover structure for all compared methods. Existing studies often solve only part of the problem: some address mobility-aware HOM–TTT adaptation without direction-aware behaviour between macro and small cells, whereas others apply ML/DRL but do not clearly retain the standard A3 trigger and radio-link-monitoring procedure. Several studies also omit a repeatable topology, fixed small-cell placement, height-aware 3D geometry, or 3GPP TR 38.901 propagation. The research gap addressed in this paper is therefore: how can a standards-compatible analytical and learning-based controller optimize HOM and TTT using both mobility state and handover direction, while fairly comparing fixed A3, analytical adaptation, Q-learning, and DQN under identical topology, propagation, FSM/RLM logic, and KPI definitions?

### Main contributions

The main contributions of this paper are as follows.

1. A realistic and repeatable two-tier HetNet is created. The paper uses hexagonal macro-cell structure with fixed small-cell locations within macro-cell coverage, yielding a better topological realism and repeatability.
2. A mobility-state- and handover-direction-aware HCP optimization framework is proposed. The proposed framework varies mobility state and handover direction (macro-to-small, small-to-macro, and same-tier handover) to adapt HOM and TTT.
3. An analytical HOM-TTT controller remains compliant with standards. The analytical controller is explainable and runs under height-aware 3D geometry and 3GPP 38.901 UMa/UMi propagation, without replacing Event A3, FSM or RLM.
4. An improved DQN-based HCP optimizer is proposed. The new DQN includes a larger discrete HOM–TTT action set, a compact normalized state, reward shaping, and specified hyperparameters with a convergence criterion [33,52].
5. Four controllers are fairly compared. The study compares fixed A3, adaptive HCP optimization, tabular Q-learning and improved DQN using the same deployment, propagation, mobility, handover, and KPI definitions.
6. A velocity-based KPI evaluation is given. The evaluation includes throughput, handover latency, handover rate, ping-pong, HOF, RLF, successful handover rate, handover success probability and outage probability at various UE speeds.

Compared with earlier ML/DRL handover studies, the key distinction of the proposed framework is that mobility state and handover direction are not treated as implicit by-products of the signal state. They are explicit control inputs for the analytical controller and explicit state variables for the learning controllers. This makes the policies interpretable in terms of macro-to-small, small-to-macro, and same-tier handover behaviour, while preserving the standard Event A3 trigger and RLM logic.

## System model and problem formulation

### Network topology and 3D geometry

Consider a two-tier heterogeneous cellular network composed of a macro-cell tier ℳ and a small-cell tier 𝒮. The set of all base stations is ℬ=ℳ∪𝒮, where $ℳ=\{1,2,...,N_{M}\}$ denotes macro base stations and $𝒮=\{1,2,...,N_{S}\}$ denotes small cells [7,8,28].

The macro-cell layer follows a regular sectorized hexagonal layout with macro-cell side length $R_{M}$ and inter-site distance


$$\begin{array}{c} D_{ISD}=\sqrt{3}R_{M}. \end{array}$$ (1)

Using axial hex-grid coordinates (*q*,*r*), the position of macro base station *m* is


$$\begin{array}{c} x_{m,2D}(q,r)=[\begin{array}{c} \sqrt{3}R_{M}\left(q+\frac{r}{2}\right)\\ \frac{3}{2}R_{M}r \end{array}]. \end{array}$$ (2)

To ensure realistic and reproducible deployment, each macro cell is divided into three sectors with orientations $\Theta =\{0^{\circ },120^{\circ },240^{\circ }\}$. One small cell is placed in each sector at a fixed radial offset *R*_sec_ as


$$\begin{array}{c} x_{s(m,a),2D}=x_{m,2D}+R_{sec}[\begin{array}{c} \cos(a)\\ \sin(a) \end{array}],\;a\in \Theta , \end{array}$$ (3)

so that the total number of small cells is $N_{S}=3N_{M}$.

Fig 1 shows the repeatable sectorized macro–small-cell topology used for all controllers. The fixed placement of small cells inside each macro sector enables consistent macro-to-small, small-to-macro, and same-tier handover evaluation under the same deployment geometry.

**Fig 1:**
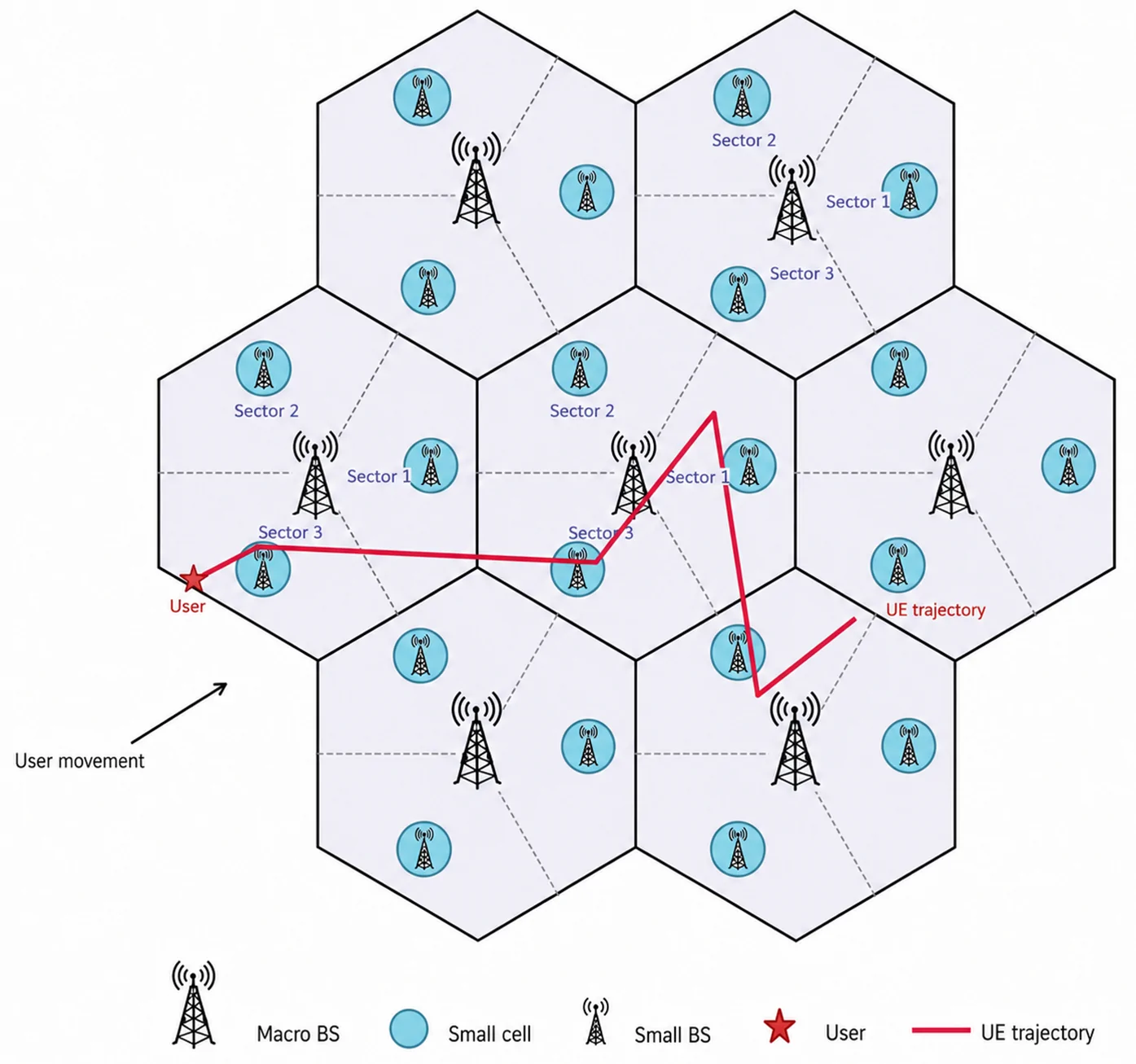
Sectorized macro–small-cell HetNet layout.

### UE mobility model

The UE moves in the horizontal plane according to


$$\begin{array}{c} q_{2D}(t)=[\begin{array}{c} x(t)\\ y(t) \end{array}],\;t\geq 0, \end{array}$$ (4)

with instantaneous speed


$$\begin{array}{c} v(t)=\left\|{\dot{q}}_{2D}(t)\right\|. \end{array}$$ (5)

In discrete time, the motion is updated as


$$\begin{array}{c} q_{2D}[k+1]=q_{2D}[k]+v[k]\Delta t[\begin{array}{c} \cos\phi [k]\\ \sin\phi [k] \end{array}], \end{array}$$ (6)

with piecewise heading update


$$\begin{array}{c} \phi [k+1]=\left\{ \begin{array}{cc} \phi [k], & k\mathrm{mod}K_{dir}\neq 0,\\ \phi_{new}, & k\mathrm{mod}K_{dir}=0. \end{array}\right. \end{array}$$ (7)

This model provides repeatable yet meaningful trajectories for handover analysis and avoids biased placement that would unfairly favour one tier [11,13].

### Height-aware 3D geometry

Let the horizontal distance between the UE and base station *i* be


$$\begin{array}{c} d_{i,2D}(t)=\left\|q_{2D}(t)-x_{i,2D}\right\|, \end{array}$$ (8)

and let $h_{i}$ and *h*_UE_ denote the base-station and UE heights [22]. The corresponding 3D distance is


$$\begin{array}{c} d_{i,3D}(t)=\sqrt{d_{i,2D}^{2}(t)+{\left(h_{i}-h_{UE}\right)}^{2}}. \end{array}$$ (9)

Fig 2 defines the 3D distance model used in the path-loss calculation. The model accounts for both horizontal UE–BS separation and the antenna-height difference, which is required for 3GPP TR 38.901 height-aware propagation evaluation.

**Fig 2:**
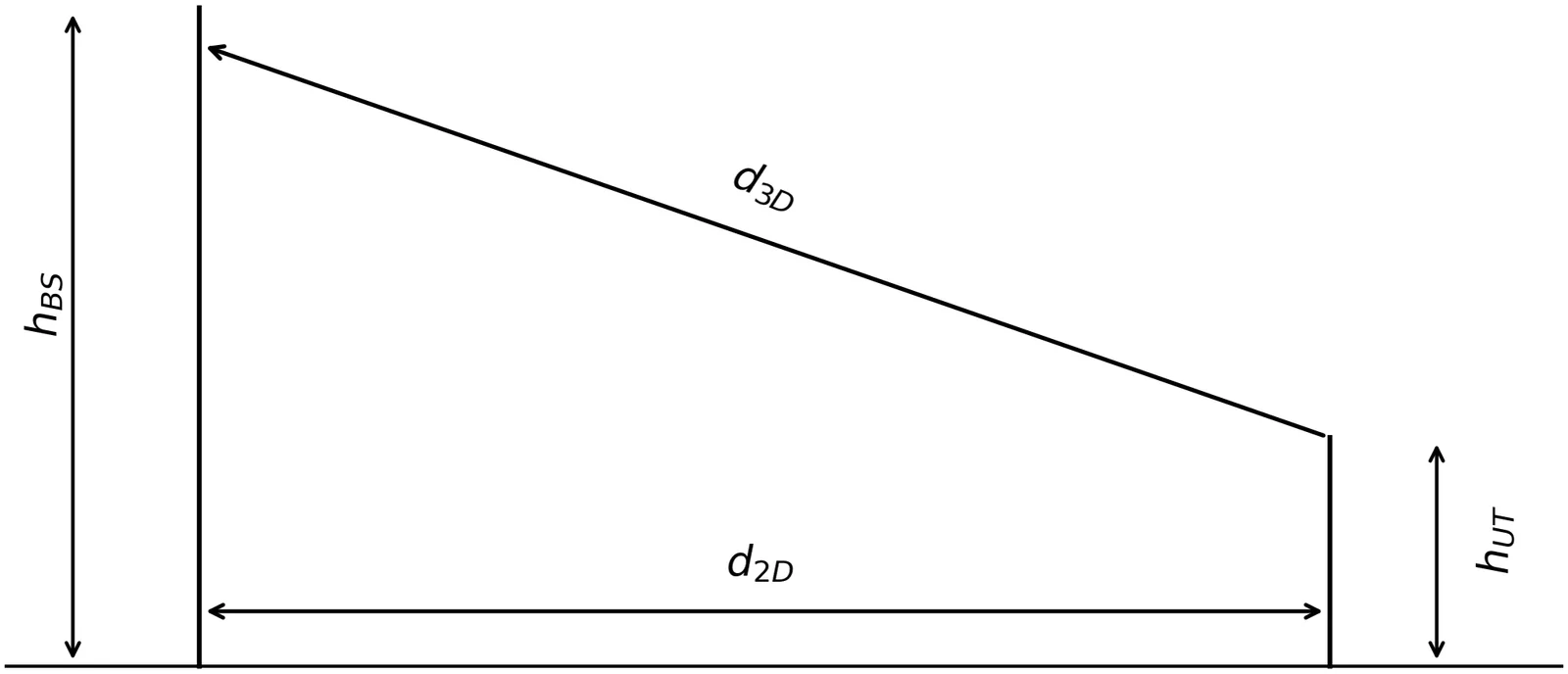
Height-aware 3D propagation geometry.

### Propagation and path-loss model (3GPP TR 38.901)

The simulation uses tier-dependent 3GPP TR 38.901 models [22,24]:


$$\begin{array}{c} PL_{i}(t)=\left\{ \begin{array}{cc} PL_{UMa}^{LOS/NLOS}, & i\in ℳ,\\ PL_{UMi}^{LOS/NLOS}, & i\in 𝒮. \end{array}\right. \end{array}$$ (10)

For the macro tier (UMa), the LOS and NLOS path-loss expressions are


$$\begin{array}{c} PL_{UMa}^{LOS}=\left\{ \begin{array}{c} 28+22\log_{10}(d_{3D})+20\log_{10}(f_{c}),\\ 28+40\log_{10}(d_{3D})+20\log_{10}(f_{c})-9\log_{10}\;(d_{BP}^{2}+(h_{BS}-h_{UE}{)}^{2}), \end{array}\right. \end{array}$$ (11)

$$\begin{array}{c} PL_{UMa}^{NLOS}=\max\;\left(PL_{UMa}^{LOS},\;13.54+39.08\log_{10}(d_{3D})+20\log_{10}(f_{c})\right). \end{array}$$ (12)

For the small-cell tier (UMi), the LOS and NLOS expressions are


$$\begin{array}{c} PL_{UMi}^{LOS}=\left\{ \begin{array}{c} 32.4+21\log_{10}(d_{3D})+20\log_{10}(f_{c}),\\ 32.4+40\log_{10}(d_{3D})+20\log_{10}(f_{c})-9.5\log_{10}\;(d_{BP}^{2}+(h_{BS}-h_{UE}{)}^{2}), \end{array}\right. \end{array}$$ (13)

$$\begin{array}{c} PL_{UMi}^{NLOS}=\max\;\left(PL_{UMi}^{LOS},\;35.3\log_{10}(d_{3D})+22.4+21.3\log_{10}(f_{c})\right). \end{array}$$ (14)

The LOS probability follows the standard distance-dependent form [22]


$$\begin{array}{c} P_{LOS}(d_{2D})=\left\{ \begin{array}{cc} 1, & d_{2D}\leq 18,\\ \frac{18}{d_{2D}}+\exp\;\left(-\frac{d_{2D}}{d_{0}}\right)\left(1-\frac{18}{d_{2D}}\right), & d_{2D}>18. \end{array}\right. \end{array}$$ (15)

### Received power, filtering, target selection, and service metrics

The large-scale received power from base station *i* at sample *k* is


$$\begin{array}{c} R_{i}[k]=P_{i}+G_{i}-PL_{i}[k]+X_{i}[k], \end{array}$$ (16)

where $P_{i}$ is the transmit power, $G_{i}$ is the effective antenna gain, and $X_{i}[k]$ represents shadowing [22]. The filtered layer-3 measurement is


$$\begin{array}{c} {\bar{R}}_{i}[k]=(1-\beta ){\bar{R}}_{i}[k-1]+\beta R_{i}[k]. \end{array}$$ (17)

The best target candidate is selected as


$$\begin{array}{c} j=\text{arg}\max_{i\in ℬ⧵\{s\}}{\bar{R}}_{i}[k]. \end{array}$$ (18)

The serving-cell SINR is


$$\begin{array}{c} \gamma_{s}[k]=\frac{P_{s}^{rx}[k]}{\sum_{\ell \in ℬ,\ell \neq s}P_{\ell }^{rx}[k]+N_{0}B_{s}}, \end{array}$$ (19)

and the instantaneous throughput is


$$\begin{array}{c} C_{s}[k]=B_{s}^{eff}[k]\log_{2}(1+\gamma_{s}[k]), \end{array}$$ (20)

where the effective bandwidth assigned to the UE is


$$\begin{array}{c} B_{s}^{eff}[k]=\frac{B_{s}}{\max(1,N_{s}^{act}[k])}. \end{array}$$ (21)

The average throughput over *K* samples is


$$\begin{array}{c} \bar{C}=\frac{1}{K}\sum_{k=1}^{K}C_{s}[k]. \end{array}$$ (22)

### Handover direction classification and Event A3 condition

The handover direction is explicitly classified as [16,19]


$$\begin{array}{c} d_{s\to j}[k]=\left\{ \begin{array}{cc} m\to s, & s\in ℳ,\;j\in 𝒮,\\ s\to m, & s\in 𝒮,\;j\in ℳ,\\ \text{same-tier}, & \text{otherwise}. \end{array}\right. \end{array}$$ (23)

The filtered target-minus-serving signal advantage is


$$\begin{array}{c} \Delta_{s,j}[k]={\bar{R}}_{j}[k]-{\bar{R}}_{s}[k]. \end{array}$$ (24)

A handover is initiated if


$$\begin{array}{c} \Delta_{s,j}[k]\geq H_{s\to j}[k] \end{array}$$ (25)

holds continuously for the selected time interval [16]


$$\begin{array}{c} T_{s\to j}[k]. \end{array}$$ (26)

### Latency and KPI formulation

The latency of handover event *h* is [20,21]


$$\begin{array}{c} L_{h}=t_{h}^{comp}-t_{h,A3}^{start} \end{array}$$ (27)

with practical approximation


$$\begin{array}{c} L_{h}\approx T_{s\to j}[k]+T_{prep}+T_{exec}. \end{array}$$ (28)

The average handover latency is


$$\begin{array}{c} {\bar{L}}_{HO}=\frac{1}{\max(1,N_{SHO})}\sum_{h=1}^{N_{SHO}}L_{h}. \end{array}$$ (29)

The successful handover probability and rate are


$$\begin{array}{c} P_{SHO}=\frac{N_{SHO}}{\max(1,N_{HO}^{att})},\;R_{SHO}=\frac{N_{SHO}}{T_{sim}}. \end{array}$$ (30)

The handover failure probability and rate are


$$\begin{array}{c} P_{HOF}=\frac{N_{HOF}}{\max(1,N_{HO}^{att})},\;R_{HOF}=\frac{N_{HOF}}{T_{sim}}. \end{array}$$ (31)

The radio-link failure probability and rate are


$$\begin{array}{c} P_{RLF}=\frac{N_{RLF}}{\max(1,K)},\;R_{RLF}=\frac{N_{RLF}}{T_{sim}}. \end{array}$$ (32)

A ping-pong event is identified as [14,20]


$$\begin{array}{c} PP_{h}=\left\{ \begin{array}{cc} 1, & c_{h-1}=c_{h+1}\text{ and }t_{h+1}-t_{h}<T_{MTS},\\ 0, & \text{otherwise}, \end{array}\right. \end{array}$$ (33)

which gives the ping-pong probability and rate


$$\begin{array}{c} P_{PP}=\frac{N_{PP}}{\max(1,N_{SHO})},\;R_{PP}=\frac{N_{PP}}{T_{sim}}. \end{array}$$ (34)

Ping-pong handover is therefore treated as a back-and-forth handover in which the UE hands over from a source cell to a target cell and then returns to the previous serving cell within a predefined minimum time-of-stay window. As UE velocity increases from 20 to 120 km/h, the UE spends progressively less time inside the target small-cell or overlap coverage region. Therefore, the probability of returning to the previous cell within the ping-pong window decreases monotonically with velocity. At high velocity, the dominant mobility problems are late handover, HOF, RLF and outage rather than repeated ping-pong handover. The overall handover probability and rate are


$$\begin{array}{c} P_{HO}=\frac{N_{HO}^{att}}{\max(1,K)},\;R_{HO}=\frac{N_{HO}^{att}}{T_{sim}}. \end{array}$$ (35)

The outage indicator and outage probability/rate are


$$\begin{array}{c} I_{out}[k]=\left\{ \begin{array}{cc} 1, & \gamma_{s}[k]<\gamma_{th},\\ 0, & \gamma_{s}[k]\geq \gamma_{th}, \end{array}\right. \end{array}$$ (36)

$$\begin{array}{c} P_{out}=\frac{1}{K}\sum_{k=1}^{K}I_{out}[k],\;R_{out}=\frac{\sum_{k=1}^{K}I_{out}[k]}{T_{sim}}. \end{array}$$ (37)

The excessive handover count, probability, and rate are


$$\begin{array}{c} N_{EHO}=N_{HO}^{att}-N_{SHO}, \end{array}$$ (38)

$$\begin{array}{c} P_{EHO}=\frac{N_{EHO}}{\max(1,N_{HO}^{att})},\;R_{EHO}=\frac{N_{EHO}}{T_{sim}}. \end{array}$$ (39)

Direction-specific handover probabilities and rates are


$$\begin{array}{c} P_{m\to s}=\frac{N_{m\to s}}{\max(1,N_{HO}^{att})},\;P_{s\to m}=\frac{N_{s\to m}}{\max(1,N_{HO}^{att})},\;P_{same}=\frac{N_{same}}{\max(1,N_{HO}^{att})}, \end{array}$$ (40)

$$\begin{array}{c} R_{m\to s}=\frac{N_{m\to s}}{T_{sim}},\;R_{s\to m}=\frac{N_{s\to m}}{T_{sim}},\;R_{same}=\frac{N_{same}}{T_{sim}}. \end{array}$$ (41)

## Proposed mobility-state- and handover-direction-aware analytical handover algorithm

In this section, the mathematical model of the proposed analytical handover controller is presented. The controller optimizes HOM and TTT depending on both the mobility state of the UE and the handover direction, while maintaining the Event A3 handover, finite-state machine (FSM), and radio-link monitoring framework [16,19].

### Design principle

Our design hinges on the physical insight that handover in heterogeneous networks is non-symmetric [15,29]. Mobility effects depend on both UE speed and handover direction (macro to small, small to macro, and same-tier). For macro-to-small handovers, high-speed UEs have a short dwell time in the target small-cell coverage or overlap region. Therefore, unnecessary small-cell entry and short-stay handovers must be avoided. Ping-pong probability is modeled to decrease monotonically as UE velocity increases, because the residence time in the target or overlap region becomes shorter and the UE is less likely to return to the previous serving cell within the minimum time-of-stay window. At high velocity, the dominant mobility problems are usually late handover, HOF, RLF, and outage. For small-to-macro exits, waiting too long at high speed increases the risk of late handover, HOF and RLF. This asymmetry is the basis of the model.

### Mobility-state and residence-time motivation

The normalized mobility state is defined as


$$\begin{array}{c} \nu [k]=\frac{v[k]-v_{\min}}{v_{\max}-v_{\min}},\;0\leq \nu [k]\leq 1, \end{array}$$ (42)

where ν[k]=0 represents low mobility and ν[k]=1 represents high mobility. The physical driver behind the asymmetric control law is the small-cell residence time


$$\begin{array}{c} \tau_{sc}=\frac{2\sqrt{R_{sc}^{2}-b^{2}}}{v}, \end{array}$$ (43)

with central-crossing special case


$$\begin{array}{c} \tau_{sc}=\frac{2R_{sc}}{v}. \end{array}$$ (44)

As speed increases, residence time decreases. Hence, a fast UE entering a small cell should be handled conservatively, whereas a fast UE leaving a small cell should be handled more aggressively.

### Signal evolution and adaptive HOM/TTT laws

The signal difference between target and serving cells is


$$\begin{array}{c} \Delta_{s,j}(t)=R_{j}(t)-R_{s}(t), \end{array}$$ (45)

and its rate of change is


$$\begin{array}{c} {\dot{\Delta }}_{s,j}(t)=-\frac{10n_{j}}{\ln10}\frac{{\dot{d}}_{j}}{d_{j}}+\frac{10n_{s}}{\ln10}\frac{{\dot{d}}_{s}}{d_{s}}. \end{array}$$ (46)

At high speed, the signals evolve more quickly. If HOM or TTT is too large, the resulting delay increases and may cause late HO, HOF, or RLF.

For macro-to-small transitions, the adaptive HOM law is


$$\begin{array}{c} H_{m\to s}[k]=H_{m\to s}^{L}+\nu [k]\left(H_{m\to s}^{H}-H_{m\to s}^{L}\right), \end{array}$$ (47)

which makes the trigger more conservative as speed increases. For small-to-macro transitions, the adaptive HOM law is


$$\begin{array}{c} H_{s\to m}[k]=H_{s\to m}^{H}-\nu [k]\left(H_{s\to m}^{H}-H_{s\to m}^{L}\right), \end{array}$$ (48)

which makes the trigger more aggressive as speed increases. The same-tier HOM is denoted by


$$\begin{array}{c} H_{same}[k]=H_{0}. \end{array}$$ (49)

The final unified HOM law is


$$\begin{array}{c} H_{s\to j}[k]=\left\{ \begin{array}{cc} H_{m\to s}[k], & m\to s,\\ H_{s\to m}[k], & s\to m,\\ H_{same}[k], & same\text{-}tier. \end{array}\right. \end{array}$$ (50)

The continuous TTT design follows the same logic:


$$\begin{array}{c} {\tilde{T}}_{m\to s}[k]=T_{m\to s}^{L}+\nu [k]\left(T_{m\to s}^{H}-T_{m\to s}^{L}\right), \end{array}$$ (51)

$$\begin{array}{c} {\tilde{T}}_{s\to m}[k]=T_{s\to m}^{H}-\nu [k]\left(T_{s\to m}^{H}-T_{s\to m}^{L}\right), \end{array}$$ (52)

$$\begin{array}{c} {\tilde{T}}_{same}[k]=T_{0}. \end{array}$$ (53)

The final TTT is quantized to the valid 3GPP set [16]


$$\begin{array}{c} {𝒯}_{3GPP}=\{0,40,64,80,100,128,160,256,320,480,512,640,1024,1280,2560,5120\}\;ms, \end{array}$$ (54)

so that


$$\begin{array}{c} T_{s\to j}[k]=\Pi_{𝒯}\;({\tilde{T}}_{s\to j}[k]). \end{array}$$ (55)

### Final decision rule and timing

The controller does not replace Event A3. It only replaces the fixed HOM and TTT with adaptive values:


$$\begin{array}{c} HO(s\to j)=\left\{ \begin{array}{cc} 1, & \Delta_{s,j}[k]\geq H_{s\to j}[k]\text{ for }T_{s\to j}[k],\\ 0, & \text{otherwise}. \end{array}\right. \end{array}$$ (56)

Fig 3 illustrates the standard Event A3 handover concept used in this study. The proposed controller does not modify the Event A3 mechanism itself; instead, it adaptively selects the HOM and TTT values applied within the standard A3 condition, preserving standards-compatible triggering while allowing timing to respond to UE mobility state and handover direction.

**Fig 3:**
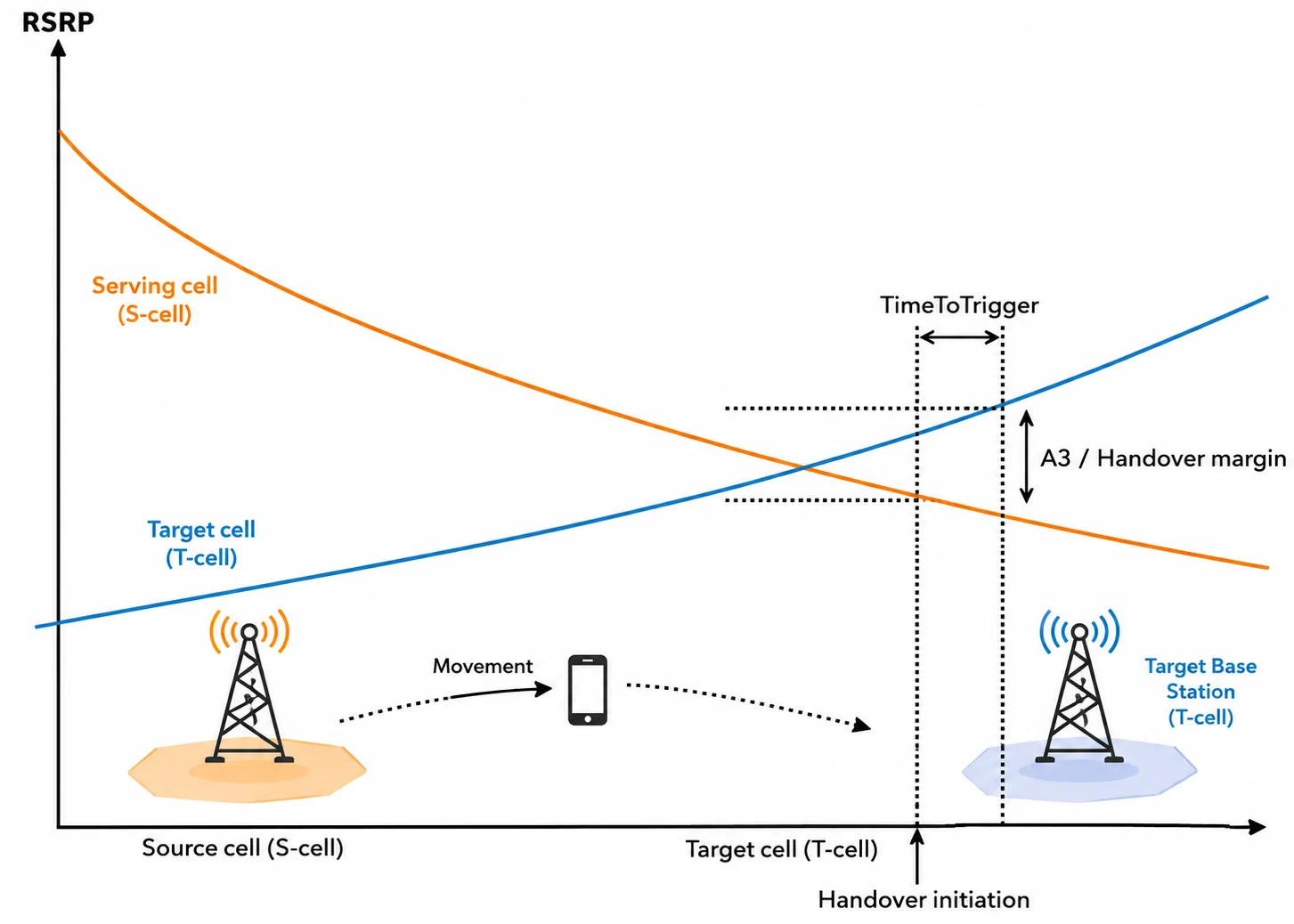
Event A3 handover timing with adaptive HOM and TTT.

Fig 4 summarizes the timing sequence used to compute handover latency and mobility-failure events. The sequence covers measurement filtering, A3 triggering, TTT holding, handover preparation, execution, completion, and RLM-based failure detection.

**Fig 4:**
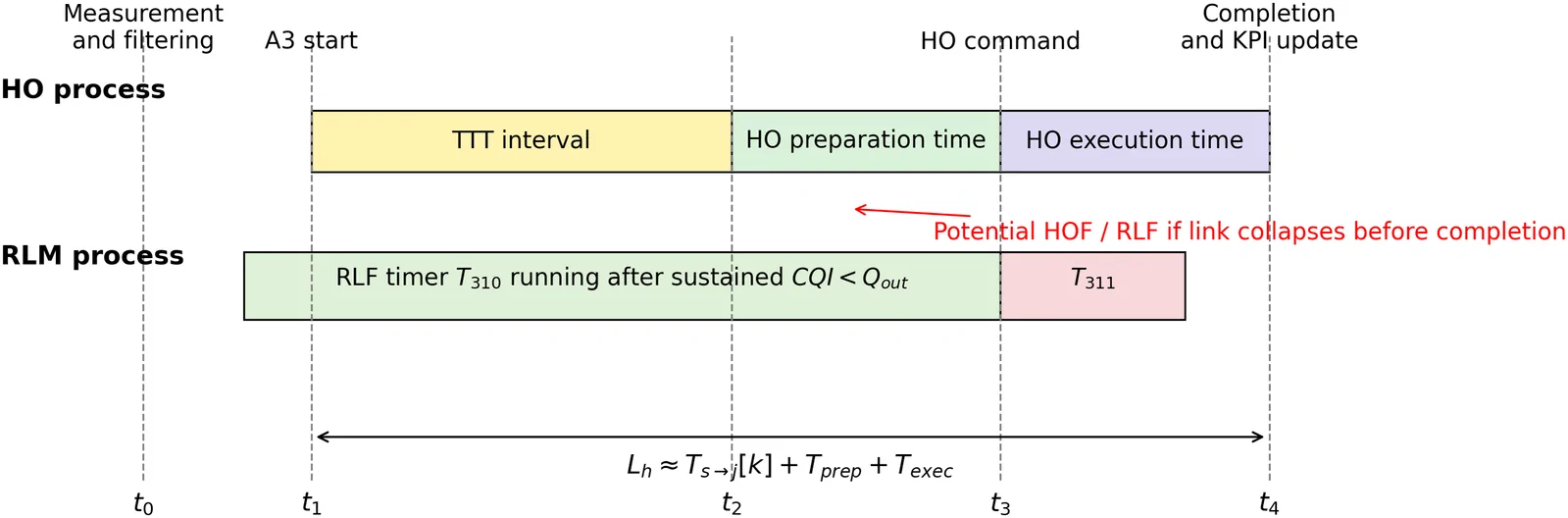
Handover timing and RLM sequence.

Fig 5 shows the workflow of the analytical controller. At each measurement step, the controller collects the UE speed and radio measurements, estimates the mobility state, determines whether the candidate handover direction is macro-to-small, small-to-macro, or same-tier, and applies the corresponding bounded HOM and TTT values. These parameters are then used in the standard Event A3 condition. If the condition remains satisfied for the configured TTT, the handover preparation and execution stages are completed; otherwise, the UE remains connected to the serving cell and measurement monitoring continues.

**Fig 5:**
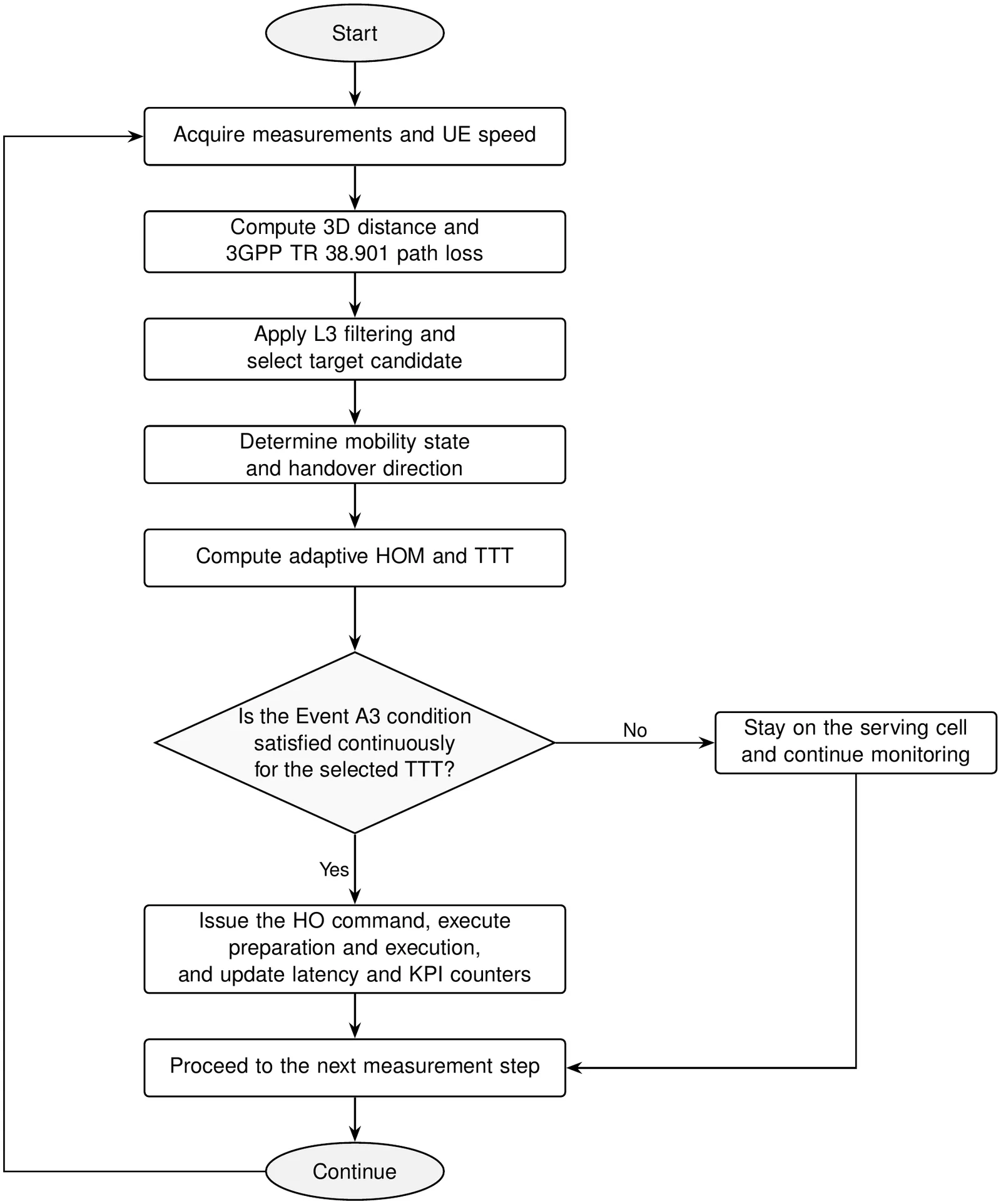
Flowchart of the proposed adaptive handover algorithm.

**Algorithm 1 Proposed adaptive handover algorithm**

1: Acquire measurements and UE speed

2: Compute 3D distance and 38.901 path loss

3: Apply L3 filtering and select target candidate

4: Determine mobility state and handover direction

5: Compute adaptive HOM and TTT

6: **if** $\Delta_{s,j}[k]\geq H_{s\to j}[k]$ continuously for the selected $T_{s\to j}[k]$ **then**

7:  Issue the HO command, execute preparation and execution, and update latency and KPI counters

8: **else**

9:  Stay on the serving cell and continue monitoring

10: **end if**

11: Proceed to the next measurement step

## Simulation methodology

The same fixed simulation environment is used for all the methods [22]. Our simulation model includes a two-tier 5G heterogeneous macro cell and small cells. The macro base stations are positioned at the centre of the hexagonal macro cells, small cells are located at regular positions inside the macro cells.

The fixed Event A3 baseline, analytical adaptive controller, tabular Q-learning controller, and DQN controller are evaluated using the same network geometry, propagation model, mobility model, Event A3 trigger, finite-state handover logic, radio-link-monitoring mechanism, and KPI definitions [16,19,20]. The only difference among the compared methods is the policy used to select HOM and TTT. Therefore, performance differences can be attributed to the HCP selection policy rather than to changes in topology, mobility, channel modelling, or performance metrics.

For the machine learning controllers, HOM and TTT are chosen from a discrete action with given bounds. The state variables are restricted to mobility state, signal quality, handover direction, and recent handover-history information. This design keeps the learning problem to mobility robustness and eliminates irrelevant performance factors [53].

Table 2 lists the common simulation parameters. These settings are shared by all four controllers so that differences in KPIs are attributable to the HOM–TTT control policy rather than to different propagation, mobility, or RLM assumptions.

**Table 2:** Main simulation parameters used in the study.

Parameter	Macro tier	Small-cell tier
Frequency range	FR1	FR2
Carrier frequency	3.5 GHz	28 GHz
System bandwidth	20 MHz	100 MHz
Transmit power	46 dBm	30 dBm
Cell model	Sectorized hexagonal macro	Fixed sector-internal small cell
Antenna height	25 m	10 m
Antenna gain	15 dBi	5 dBi
Path-loss model	3GPP TR 38.901 UMa	3GPP TR 38.901 UMi-Street-Canyon

Common parameters: $Q_{out}=-8dB$; $Q_{in}=-6dB$; UE height = 1.5 m; Event A3 triggering; L3 filter coefficient β=0.2; *T*_310_ = 200 ms; *T*_311_ = 1000 ms; *N*_310_ = 1; *N*_311_ = 1; UE measurement periodicity = 40 ms; velocities {20,40,60,80,100,120} km/h; three seeds per method [16,19,22].

Three independent stochastic runs were performed for each controller and tested for 6 different UE velocities. Observed run to run variation is summarized by mean, SD, SE and 95% confidence intervals and interpreted descriptively.

Training and evaluation were not combined. After training, the learned policy was frozen and greedy actions were used. The evaluation transitions did not change the Q-table, replay memory, online network, or target network.

The measurement interval of the simulator is 0.04 s and *K* = *T*_sim_/0.04. In the DQN replay formulation, the terminal flag denotes the final transition of an episode. Both learning controllers are trained over 300 training episodes (Q-learning: α=0.1, γ=0.97, ϵ is decreased linearly from 1.0 to 0.05 according to ϵ=max(0.05,ϵ−0.95/300)).

Q-learning is implemented on 5 discretized state variables, i.e., UE velocity, gap between UE and serving-target signal, serving-cell signal to interference ratio (SINR), handover direction, and time since the last successful handover, resulting in a 5×5×4×3×4 grid (1200 states).

The DQN has seven inputs, a 7–32–32–35 architecture, a learning rate of $8\times 10^{-4}$, a gamma value of 0.97, a replay capacity of 20,000, a batch size of 32 and a target-network that is synchronized every eight episodes. The implementation uses Adam gradient descent.

Propagation is based on the 3GPP TR 38.901 UMa/UMi propagation model that incorporates stochastic shadowing. All the controllers have the same propagation and mobility assumptions, with the differences being the HOM–TTT control policies.

### Ethics statement

This study does not involve human participants, animals, or personal data. All simulations were conducted using standard models and publicly available technical specifications.

## Machine learning-based HCP optimization

### State representation, action space, and reward

The learning-based controllers are included as comparative baselines under the same handover framework used by the analytical method. Both RL controllers optimize HOM and TTT only and do not replace Event A3, FSM, or radio-link monitoring.

Table 3 shows that both learning controllers use the same HOM–TTT action set, while the DQN receives a richer continuous-state description.

**Table 3:** State representation and action space used by the Q-learning and DQN controllers.

State	Definition	Used by
*s*_1_	Normalized UE speed	Q-learning, DQN
*s*_2_	Normalized filtered serving–target signal gap $\Delta_{s,j}$	Q-learning, DQN
*s*_3_	Normalized serving-cell SINR	Q-learning, DQN
*s*_4_	Handover-direction code (macro→small, small→macro, same-tier)	Q-learning, DQN
*s*_5_	Normalized time since the last successful handover	Q-learning, DQN
*s*_6_	Normalized derivative of the filtered signal gap	DQN only
*s*_7_	Serving-tier indicator	DQN only

Action space: discrete HOM–TTT pairs with HOM ∈{1.0,2.0,2.5,3.0,3.5,4.0,5.0} dB and TTT ∈{40,80,160,240,320} ms, yielding 35 valid actions. The DQN uses the richer state vector, while Q-learning uses the compact state vector to preserve a lightweight tabular implementation.

The reward is not a fixed ranking of controllers, but reflects the mobility-control goals. There is a bigger penalty for service continuity failures and outage and a moderate penalty for ping-pong, latency and excessive handovers. The Q-learning coefficients used in the reported configuration are $w_{C}=1.0$, $w_{S}=1.2$, $w_{L}=0.45$, $w_{H}=2.0$, $w_{R}=2.5$, $w_{P}=0.9$, $w_{O}=1.8$, and $w_{E}=0.35$:


$$\begin{array}{c} r_{k}^{Q}=w_{C}{\tilde{C}}_{k}+w_{S}I_{succ,k}-w_{L}{\tilde{L}}_{k}-w_{H}I_{HOF,k}-w_{R}I_{RLF,k}-w_{P}I_{PP,k}-w_{O}I_{out,k}-w_{E}I_{EHO,k}, \end{array}$$ (57)

Here ${\tilde{C}}_{k}$ is normalized throughput, $I_{succ,k}$ indicates successful handover, ${\tilde{L}}_{k}$ is normalized handover latency, and $I_{HOF,k}$, $I_{RLF,k}$, $I_{PP,k}$, $I_{out,k}$, and $I_{EHO,k}$ denote the corresponding mobility events.

The DQN uses the same basic reward logic but adds smoothness and direction-consistency terms:


$$\begin{array}{c} r_{k}^{DQN}=r_{k}^{Q}-w_{\Delta H}|H_{k}-H_{k-1}|-w_{\Delta T}|T_{k}-T_{k-1}|+w_{D}I_{dir,k}, \end{array}$$ (58)

DQN maintains the original reward, but adds action-smoothness and handover-direction-consistency terms. The reward priorities and hyperparameters were chosen in the light of engineering considerations, rather than the optimisation of one reported outcome, based on the severity of the KPI.

Table 4 gives a qualitative sensitivity interpretation based on the mechanisms, and not a separate numerical reward-weight experiment. Consequently, the reported reward configuration is the only one that the conclusions refer to.

**Table 4:** Reward-weight sensitivity and expected convergence implications.

Weight group	Primary role	If too small	If too large
$w_{H},w_{R},w_{O}$	Penalize severe service-continuity events	Failure events remain weakly separated from normal transitions; convergence may be fast but toward an unsafe policy	Sparse large penalties dominate the return, increase reward variance and can yield over-conservative HOM–TTT actions
$w_{P},w_{E}$	Limit ping-pong and unnecessary handovers	Action chattering and high signalling load can persist	Necessary exits may be postponed, degrading HOF/RLF/outage performance
$w_{C},w_{S}$	Reward throughput and successful handover	The agent can converge to a robust but low-throughput policy	Aggressive triggering may improve short-term reward while increasing oscillation and failures
$w_{L}$	Penalize handover latency	Delayed handovers may be weakly discouraged	Excessively short TTT and aggressive triggering may increase ping-pong, unnecessary handovers, or failures
$w_{\Delta H},w_{\Delta T}$	Smooth successive HOM–TTT actions	Noisy action switching broadens the moving-average reward band	Adaptation to rapid mobility changes becomes sluggish and convergence can plateau prematurely
$w_{D}$	Encourage direction-consistent actions	Macro-to-small and small-to-macro asymmetry is underused	The policy can over-prioritize direction labels over current radio conditions

### Q-learning controller

The Q-learning controller uses a tabular action-value representation over the compact discrete state-action space and updates the Q-table according to


$$\begin{array}{c} Q(s_{k},a_{k})\leftarrow Q(s_{k},a_{k})+\alpha \left[r_{k}+\gamma \max_{a^{\prime }}Q(s_{k+1},a^{\prime })-Q(s_{k},a_{k})\right]. \end{array}$$ (59)

**Algorithm 2 Q-learning controller for HOM–TTT optimization**

1: Initialize Q-table *Q*(*s*,*a*) and hyperparameters

2: **for** each training episode **do**

3:  Observe current state $s_{k}$

4:  **while** episode not terminated **do**

5:   Select HOM–TTT action $a_{k}$ using an exploration/exploitation rule

6:   Apply $a_{k}$ in the handover environment

7:   Execute the standard A3-based handover procedure

8:   Observe reward $r_{k}$ and next state $s_{k+1}$

9:   Update the Q-table

10:   Set $s_{k}\leftarrow s_{k+1}$

11:  **end while**

12: **end for**

13: Output the learned policy $\pi_{Q}(s)=\text{arg}\max_{a}Q(s,a)$

Fig 6 shows the workflow of the tabular Q-learning controller. At each interaction step, the controller observes the discretized mobility and radio state and chooses a HOM–TTT action through an epsilon-greedy exploration–exploitation policy. The selected action is applied within the same Event A3/FSM/RLM handover process. After the KPI-based reward and next state are observed, the corresponding Q-table entry is updated according to the Bellman learning rule. This process continues throughout the training episodes until the learned policy selects the action with the highest Q-value for each discretized state.

**Fig 6:**
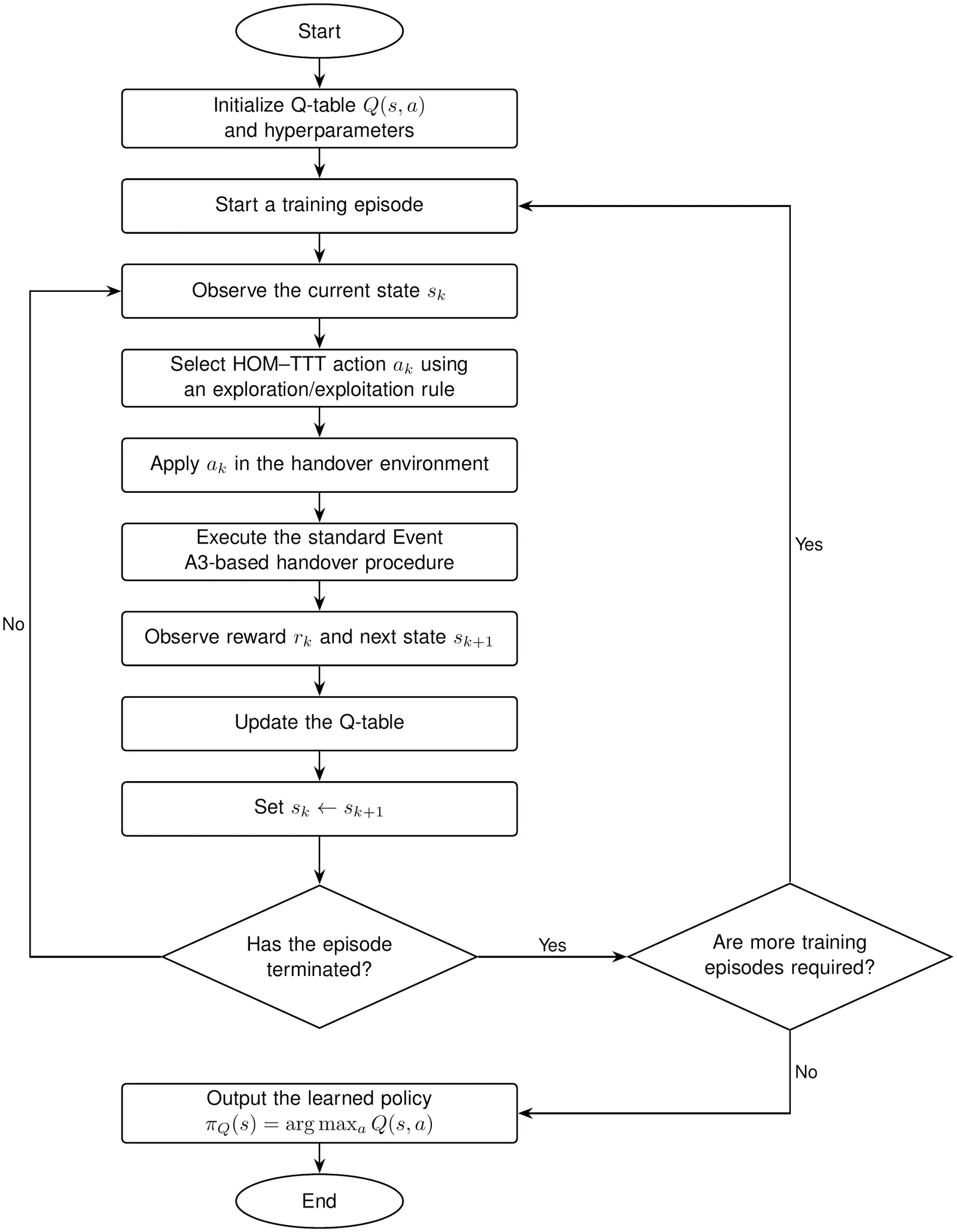
Flowchart of the Q-learning handover controller.

The Q-learning process flow is as follows: start, initialize the Q-table and hyperparameter, observe current state, select an action from HOM-TTT using an exploration/exploitation approach, apply the action in the handover environment, perform handover using A3, receive reward and next state, update Q-table, check if episode ends, if not then continue training, output the learned policy.

### Improved DQN controller

The DQN controller approximates the action-value function using a neural network and is intended to handle richer state structure more effectively than tabular Q-learning

Table 5 reports the DQN architecture and hyperparameters used for reproducibility, including hidden-layer size, learning rate, replay-buffer size, mini-batch size, target-network update period, and number of training episodes.

**Table 5:** DQN controller hyperparameters used in the reported configuration.

Hyperparameter	Value
Online-network hidden layers	32, 32
Learning rate	$8\times 10^{-4}$
Discount factor γ	0.97
Replay-buffer size	20,000 transitions
Mini-batch size	32
Target-network update period	8 episodes
ϵ-greedy schedule	1.0→0.05 with decay
Training episodes	300
Action space	35 HOM–TTT actions

The DQN input layer contains the seven normalized state variables in Table 3; two fully connected ReLU hidden layers of 32 neurons approximate the action-value function; and the output layer contains 35 neurons corresponding to the discrete HOM–TTT actions. This architecture has 2,467 trainable parameters in the online network. Training uses replay memory to decorrelate consecutive mobility samples, a target network to stabilize bootstrapped targets, and an ϵ-greedy schedule to move from exploration to exploitation. Convergence is assessed through the moving-average training reward and by the stabilization of KPI trends near the end of training. The 32–32 architecture is selected as a compact compromise: smaller networks may under-represent the continuous speed/SINR/direction interaction, whereas larger networks increase training variability and computational cost without changing the standard A3/FSM/RLM decision structure.

**Algorithm 3 Improved DQN controller for HOM–TTT optimization**

1: Initialize online network $Q_{\theta }$, target network $Q_{\theta^{-}}$, replay buffer 𝒟, and hyperparameters

2: **for** each training episode **do**

3:  Observe current state $s_{k}$

4:  **while** episode not terminated **do**

5:   Select HOM–TTT action $a_{k}$ using an ϵ-greedy policy

6:   Apply $a_{k}$ in the handover environment

7:   Execute the standard A3-based handover procedure

8:   Observe reward $r_{k}$ and next state $s_{k+1}$

9:   Store $\left(s_{k},a_{k},r_{k},s_{k+1}\right)$ in replay memory

10:   Sample a mini-batch from 𝒟

11:   Compute target Q-values using $Q_{\theta^{-}}$

12:   Update the online network by gradient descent

13:   Periodically update the target network

14:   Set $s_{k}\leftarrow s_{k+1}$

15:  **end while**

16: **end for**

17: Output the trained DQN policy $\pi_{DQN}(s)=\text{arg}\max_{a}Q_{\theta }(s,a)$

Fig 7 shows the training procedure of the improved DQN controller. At each interaction step, the controller observes the normalized continuous state and selects a discrete HOM–TTT action through an epsilon-greedy policy. The selected action is executed through the unchanged Event A3/FSM/RLM handover procedure, after which the reward, next state, and terminal status are stored in replay memory. A mini-batch is then sampled to calculate the target Q-values, update the online network through gradient descent, and periodically synchronize the target network. After training, a forward pass through the online network is used to select the HOM–TTT action with the highest Q-value.

**Fig 7:**
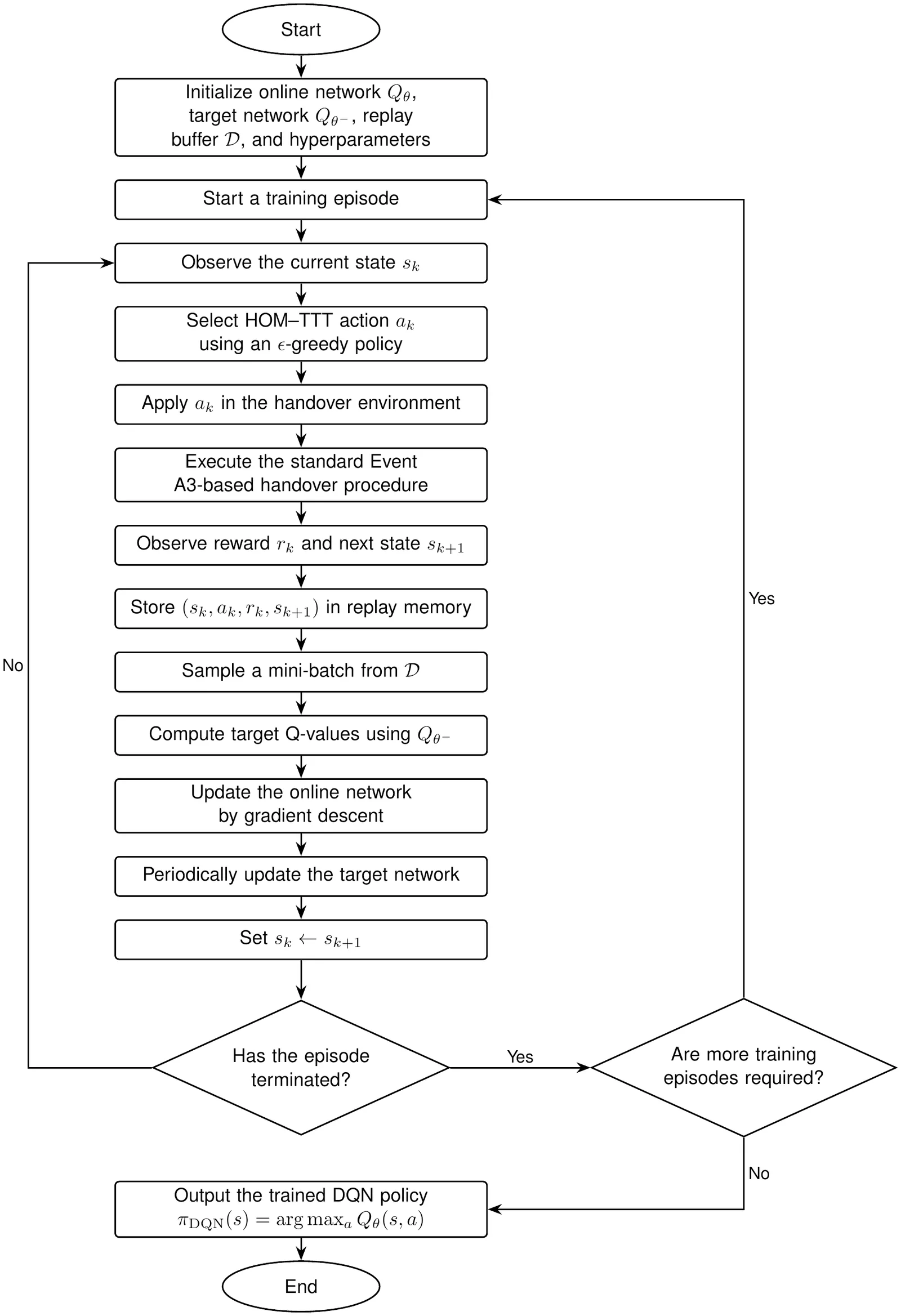
Flowchart of the improved DQN handover controller.

The DQN flowchart follows the same steps as Q-learning but adds experience storage in replay memory, mini-batch sampling, target-value computation, online-network updating, and periodic target-network synchronization. Because it uses a richer normalized state representation than tabular Q-learning, it can handle larger and more continuous state spaces while still operating under the same Event A3/FSM/RLM handover framework.

### Computational complexity and implementation cost

For Q-learning, the online decision cost is O(|𝒜|) when the best HOM–TTT action is found by scanning all available actions, while the Q-table has a memory cost of O(|𝒮||𝒜|). The update is lightweight, although it depends on discretizing the state space. For DQN, online inference requires a single forward pass. The 7–32–32–35 network contains 2,467 trainable parameters and requires approximately 2,368 multiply–accumulate operations per decision. With 32-bit parameters, one online network occupies approximately 9.6 kB, while the target copy is required only during training. Training memory is dominated by the replay buffer of 20,000 transitions, which requires approximately 1.3–1.5 MB for two seven-element float states together with the action, reward, and terminal metadata. The deployed inference module does not require either the replay buffer or the target network.

Real-time integration is therefore mainly a systems-engineering issue rather than a neural-network size issue. A practical implementation must collect and normalize the seven state variables, perform inference within the mobility-control cycle, map the selected action to operator-approved HOM–TTT values, and apply guardrails for minimum residence time, parameter-change rate, and fallback to fixed A3. Offline training can be carried out in a centralized SON or non-real-time RAN-intelligence function, while the compact inference policy can operate closer to the radio controller. Before activation, the controller should be assessed in shadow mode using action logging, latency profiling, rollback criteria, and vendor-specific RRC constraints. Centralized operation must also account for telemetry delay, synchronization, signalling overhead, and simultaneous requests from multiple UEs. Because wall-clock training and inference times depend strongly on processor, numerical precision, software framework, and batching, this study reports architecture-level operation counts and memory requirements rather than platform-specific timing values. Deployment-oriented timing should be measured together with the corresponding hardware and software configuration. The analytical controller remains the least costly option; Q-learning provides lightweight table lookup; and DQN offers stronger state generalization with a small inference footprint, although it has higher offline-training and operational-integration costs.

## Results and discussion

This section directly compares the four controllers in terms of velocity on the basis of the common sectorized hexagonal deployment, propagation model, Event A3 triggering, FSM/RLM logic, and KPI definitions. This section first presents an overview of the learning-based controllers, then examines the HOM, TTT and velocity sensitivities of the analytical controller, and finally compares the fixed A3 baseline, analytical adaptive controller, tuned Q-learning, and improved DQN controllers over the full mobility spectrum.

### Convergence of Q-learning and DQN

Fig 8 shows the convergence of the training reward for the Q-learning and improved DQN controllers after 300 episodes. The light curves show the episode-level reward fluctuations due to exploration and mobility pattern variations, and the dark moving-average curves show the learning progress. In the Q-learning situation, the reward starts during the early stages from a low value and then settles at a middle value. This slower convergence is expected as the tabular controller is using a discrete state representation and thus cannot completely capture fine features in the velocity, gap, SINR, handover direction and handover history.

**Fig 8:**
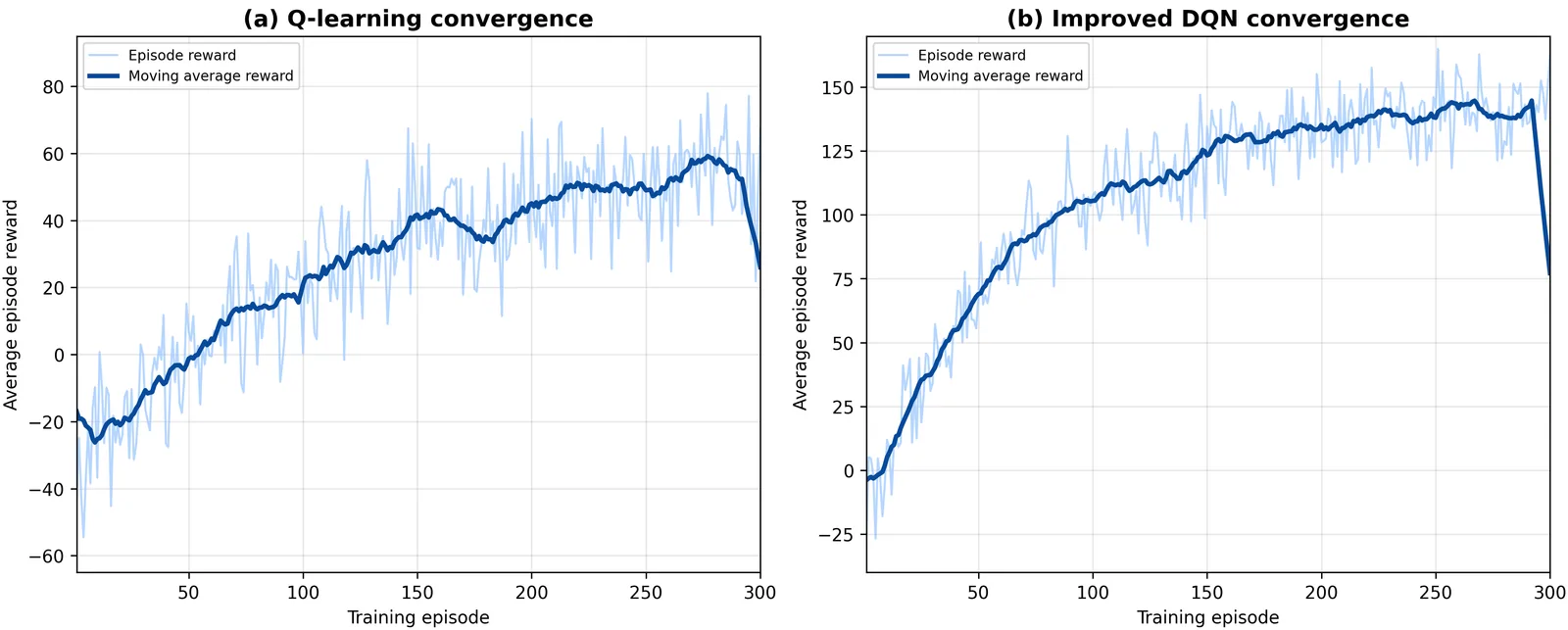
Convergence of the training reward for the Q-learning and DQN.

For the configuration tested, DQN achieves a higher observed moving-average reward, and stabilizes earlier than Q-learning. The reported mobility KPIs are used separately to assess controller effectiveness.

### Ablation and sensitivity interpretation

A component-level ablation interpretation is obtained from the implemented control laws and the sensitivity results. With both awareness terms present, the controller adapts to UE speed and treats macro-to-small and small-to-macro transitions asymmetrically. If mobility-state awareness is removed, the policy remains direction-aware but cannot adapt to changes in residence time as velocity varies. If handover-direction awareness is removed, the policy remains speed-aware but treats small-cell entry and exit symmetrically. If both awareness terms are removed, the controller reduces to fixed or state-independent HOM–TTT selection. For DQN, removing the smoothness terms would also remove the explicit penalty on successive HOM–TTT oscillations. Therefore, this subsection provides a mechanism-level ablation interpretation rather than a separately retrained numerical ablation study. Figs 9–11 provide the supporting HOM, TTT, and velocity sensitivity evidence; quantitative component-removal experiments are identified as future work.

### Sensitivity of the analytical controller to HOM, TTT, and velocity

Fig 9 shows the traditional HOM trade-off. Raising HOM decreases the average number of handovers and eliminates ping-pong, but at the same time increases HOF and RLF due to conservative handover. So the probability of SHO decreases at large HOM values. This explains why HOM should be selected adaptively.

**Fig 9:**
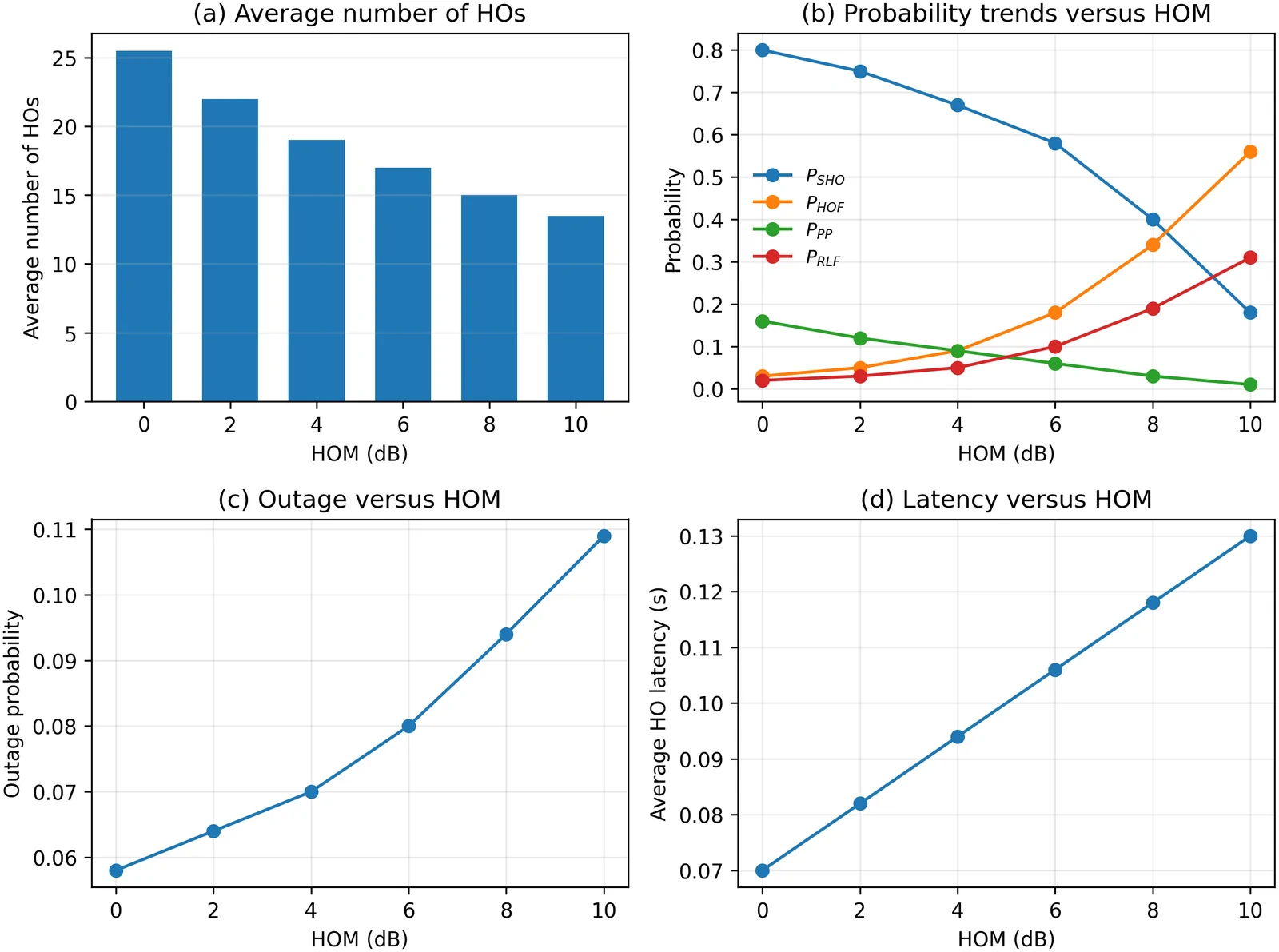
Sensitivity of average and probability-based KPIs to HOM.

Fig 10 shows the TTT trade-off. Short TTT values produce aggressive triggering, which increases the average number of handovers and the ping-pong probability. Long TTT reduces unnecessary handovers but increases HOF, RLF, outage, and latency by delaying handover.

**Fig 10:**
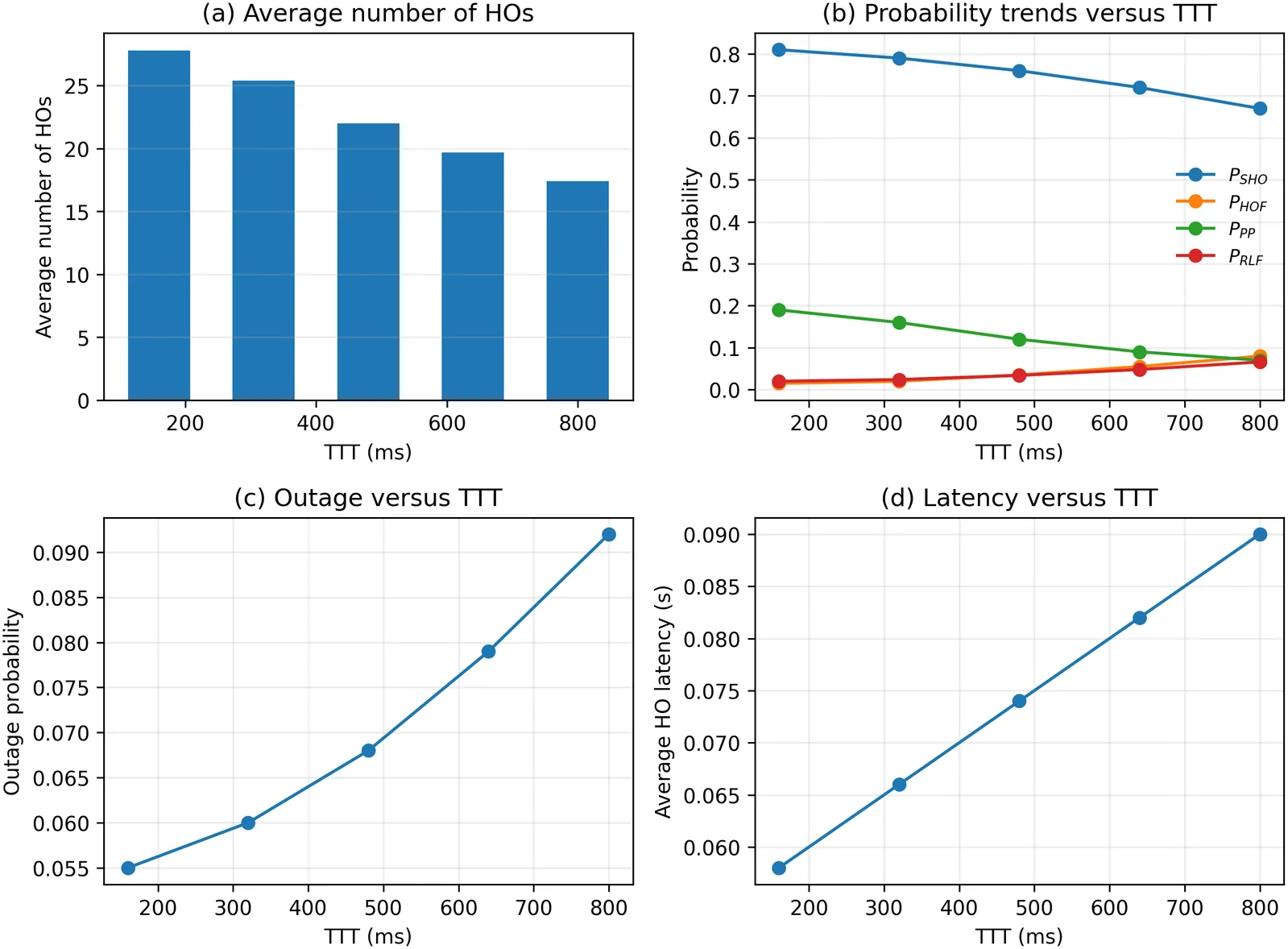
Sensitivity of average and probability-based KPIs to TTT.

Fig 11 shows that higher velocity degrades mobility robustness. The average number of handovers is higher with velocity because the UE crosses more cell regions during the same observation interval. SHO decreases, while HOF, RLF, outage, and latency increase. Ping-pong follows a different physical trend: it decreases with velocity because the UE spends less time in the target small-cell or overlap region and is less likely to return to the previous serving cell within the minimum time-of-stay window. These trends give the physical motivation to make the controller mobility-state- and handover-direction-aware.

**Fig 11:**
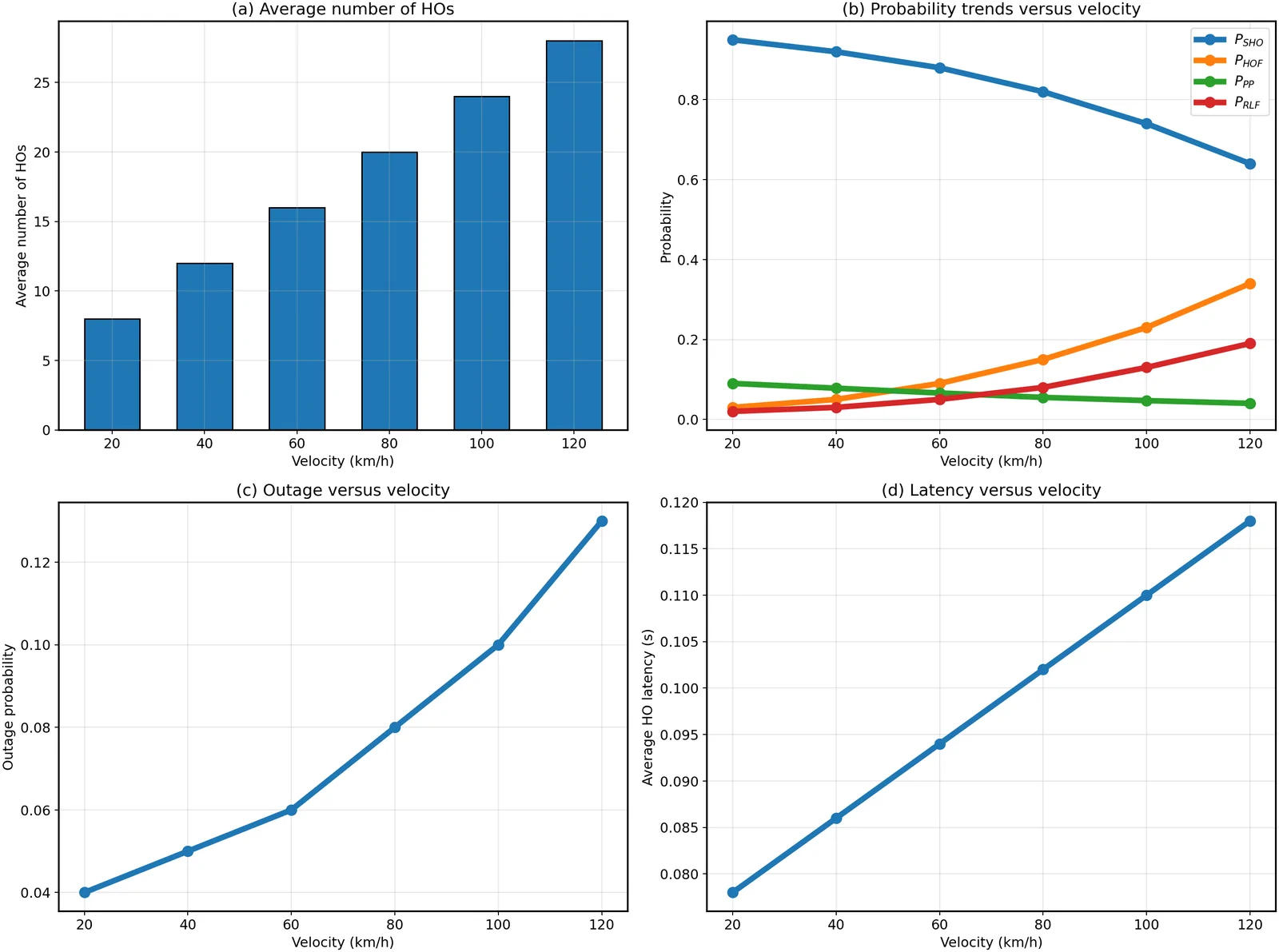
Velocity sensitivity of the analytical controller.

### Velocity-based comparison of the four controllers

Figs 12 and 13 show the fair comparison between fixed A3, analytical adaptation, tabular Q-learning and DQN. The methods degrade with increasing velocity because higher velocity reduces small-cell dwell time, increases target/serving signal transitions, and leaves less time for robust handover execution. The fixed A3 baseline suffers the most. Throughput and SHO decrease with velocity, whereas HOF, RLF, outage, latency and late-handover-related metrics increase. Ping-pong is treated separately: its probability decreases monotonically with velocity because the UE spends less time in the target small-cell or overlap region and is less likely to return to the previous serving cell within the ping-pong time window. Across the full velocity range, the ranking remains consistent: fixed A3 has the highest ping-pong probability, analytical adaptation is lower, Q-learning is lower than the analytical controller, and DQN gives the lowest ping-pong probability.

**Fig 12:**
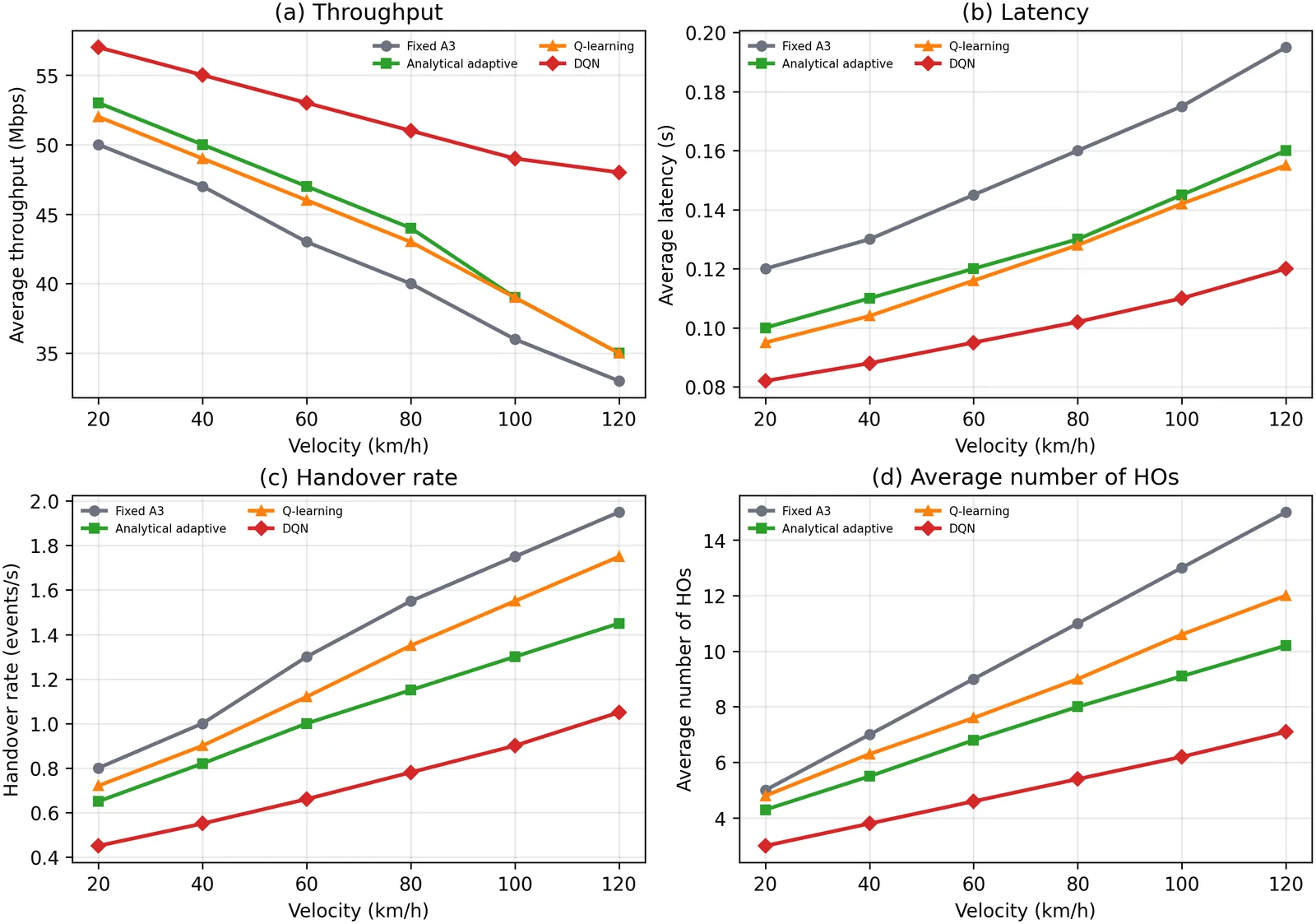
Velocity-based comparison of average throughput, latency, handover rate, and average number of handovers.

**Fig 13:**
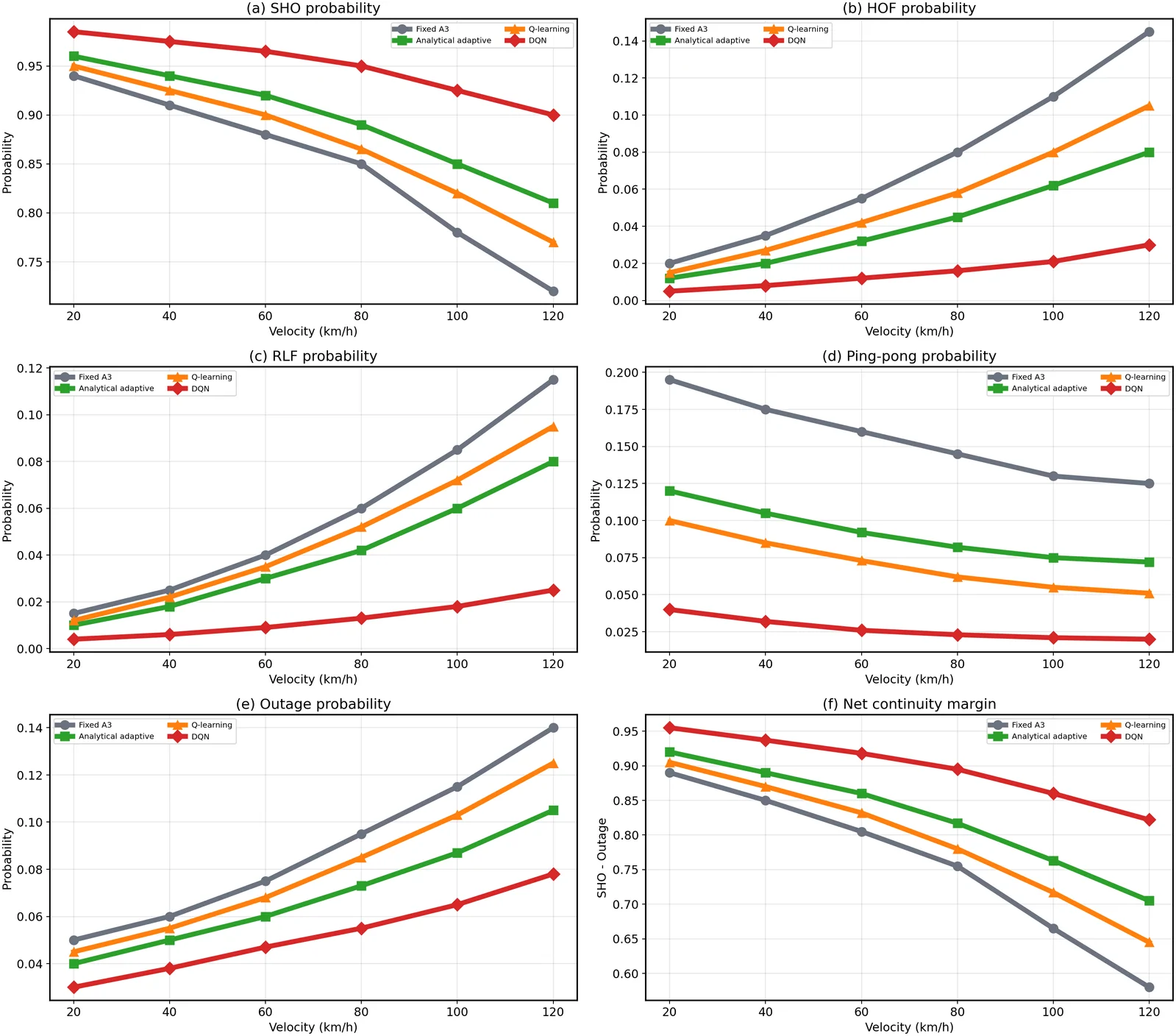
Velocity-based comparison of SHO, HOF, RLF, ping-pong, outage, and the net continuity margin.

The analytical controller is a useful and intuitive reference point. Because it adapts HOM and TTT based on mobility state and handover direction, it improves throughput, outage and robustness failures compared with the fixed baseline. The proposed analytical, Q-learning and DQN methods reduce ping-pong by adapting HOM/TTT more conservatively for macro-to-small transitions at high velocity, thereby avoiding unnecessary small-cell entry and short-stay handovers. At the same time, small-to-macro exits remain sufficiently responsive to avoid late handover, HOF, RLF and outage. The analytical approach thus provides a clear standards-compatible solution when simplicity is crucial.

From the simulation scenarios investigated, the mean values obtained for tabular Q-learning are better than the fixed A3 baseline for throughput, successful handover probability, HOF, RLF, ping-pong and outage. The reward assigns a larger penalty to severe service-continuity events than to a normal handover attempt, which discourages an excessively aggressive policy. Several Q-learning KPI means are nevertheless less favourable than those observed for DQN, consistent with the limitation of a discretized tabular state when speed, signal evolution, direction and handover history interact continuously.

Across the evaluated velocity range, DQN has the largest observed mean throughput and successful-handover probability and the smallest observed mean latency, handover rate, HOF, RLF, ping-pong, and outage among the four controllers. These trends are consistent with the richer normalized state representation, replay memory, target-network stabilization, direction-aware reward extension, and constrained HOM–TTT action space. Accordingly, the result is described as the best observed mean performance under the tested simulation conditions.

Table 6 reports the seed-level values used in the analysis together with the corresponding descriptive summary statistics.

**Table 6:** Average performance across all tested velocities—mean, SD, SE, and 95% CI.

(6a) Throughput (Mbps)							
Method	Mean	Seed 1	Seed 2	Seed 3	SD	SE	95% CI
Fixed A3	41.50	41.00	41.50	42.00	0.50	0.29	[40.26, 42.74]
Analytical adaptive	44.67	44.17	44.67	45.17	0.50	0.29	[43.43, 45.91]
Q-learning	44.00	43.50	44.00	44.50	0.50	0.29	[42.76, 45.24]
DQN	52.17	51.67	52.17	52.67	0.50	0.29	[50.93, 53.41]
**(6b) Latency (s)**							
**Method**	**Mean**	**Seed 1**	**Seed 2**	**Seed 3**	**SD**	**SE**	**95% CI**
Fixed A3	0.154	0.149	0.154	0.159	0.005	0.003	[0.142, 0.166]
Analytical adaptive	0.128	0.123	0.128	0.133	0.005	0.003	[0.116, 0.140]
Q-learning	0.123	0.118	0.123	0.128	0.005	0.003	[0.111, 0.135]
DQN	0.100	0.095	0.100	0.105	0.005	0.003	[0.088, 0.112]
**(6c) HO rate (events/s)**							
**Method**	**Mean**	**Seed 1**	**Seed 2**	**Seed 3**	**SD**	**SE**	**95% CI**
Fixed A3	1.392	1.342	1.392	1.442	0.050	0.029	[1.268, 1.516]
Analytical adaptive	1.062	1.012	1.062	1.112	0.050	0.029	[0.938, 1.186]
Q-learning	1.232	1.182	1.232	1.282	0.050	0.029	[1.108, 1.356]
DQN	0.732	0.682	0.732	0.782	0.050	0.029	[0.608, 0.856]
**(6d) SHO probability**							
**Method**	**Mean**	**Seed 1**	**Seed 2**	**Seed 3**	**SD**	**SE**	**95% CI**
Fixed A3	0.847	0.837	0.847	0.857	0.010	0.006	[0.822, 0.872]
Analytical adaptive	0.895	0.885	0.895	0.905	0.010	0.006	[0.870, 0.920]
Q-learning	0.872	0.862	0.872	0.882	0.010	0.006	[0.847, 0.897]
DQN	0.950	0.940	0.950	0.960	0.010	0.006	[0.925, 0.975]
**(6e) HOF probability**							
**Method**	**Mean**	**Seed 1**	**Seed 2**	**Seed 3**	**SD**	**SE**	**95% CI**
Fixed A3	0.074	0.068	0.074	0.080	0.006	0.003	[0.059, 0.089]
Analytical adaptive	0.042	0.036	0.042	0.048	0.006	0.003	[0.027, 0.057]
Q-learning	0.055	0.049	0.055	0.061	0.006	0.003	[0.040, 0.070]
DQN	0.015	0.009	0.015	0.021	0.006	0.003	[0.000, 0.030]
**(6f) RLF probability**							
**Method**	**Mean**	**Seed 1**	**Seed 2**	**Seed 3**	**SD**	**SE**	**95% CI**
Fixed A3	0.057	0.052	0.057	0.062	0.005	0.003	[0.045, 0.069]
Analytical adaptive	0.040	0.035	0.040	0.045	0.005	0.003	[0.028, 0.052]
Q-learning	0.048	0.043	0.048	0.053	0.005	0.003	[0.036, 0.060]
DQN	0.013	0.008	0.013	0.018	0.005	0.003	[0.001, 0.025]
**(6g) Ping-pong probability**							
**Method**	**Mean**	**Seed 1**	**Seed 2**	**Seed 3**	**SD**	**SE**	**95% CI**
Fixed A3	0.155	0.149	0.155	0.161	0.006	0.003	[0.140, 0.170]
Analytical adaptive	0.091	0.085	0.091	0.097	0.006	0.003	[0.076, 0.106]
Q-learning	0.071	0.065	0.071	0.077	0.006	0.003	[0.056, 0.086]
DQN	0.027	0.021	0.027	0.033	0.006	0.003	[0.012, 0.042]
**(6h) Outage probability**							
**Method**	**Mean**	**Seed 1**	**Seed 2**	**Seed 3**	**SD**	**SE**	**95% CI**
Fixed A3	0.089	0.083	0.089	0.095	0.006	0.003	[0.074, 0.104]
Analytical adaptive	0.069	0.063	0.069	0.075	0.006	0.003	[0.054, 0.084]
Q-learning	0.080	0.074	0.080	0.086	0.006	0.003	[0.065, 0.095]
DQN	0.052	0.046	0.052	0.058	0.006	0.003	[0.037, 0.067]

*Note: n* = 3 seeds, *df* = 2, *t*_0.975,2_ = 4.303, and 95% CI = mean ±t×SE. Close or overlapping intervals should be interpreted cautiously.

As shown in Table 6, DQN has the highest mean throughput and successful-handover probability and the lowest mean latency, handover rate, HOF, RLF, ping-pong and outage among the evaluated controllers.

The policy-level means with 95% confidence intervals are displayed in Fig 14. DQN shows the best observed mean multi-KPI trend under the tested simulation conditions, while the intervals represent descriptive run-to-run variation across the three evaluation runs.

**Fig 14:**
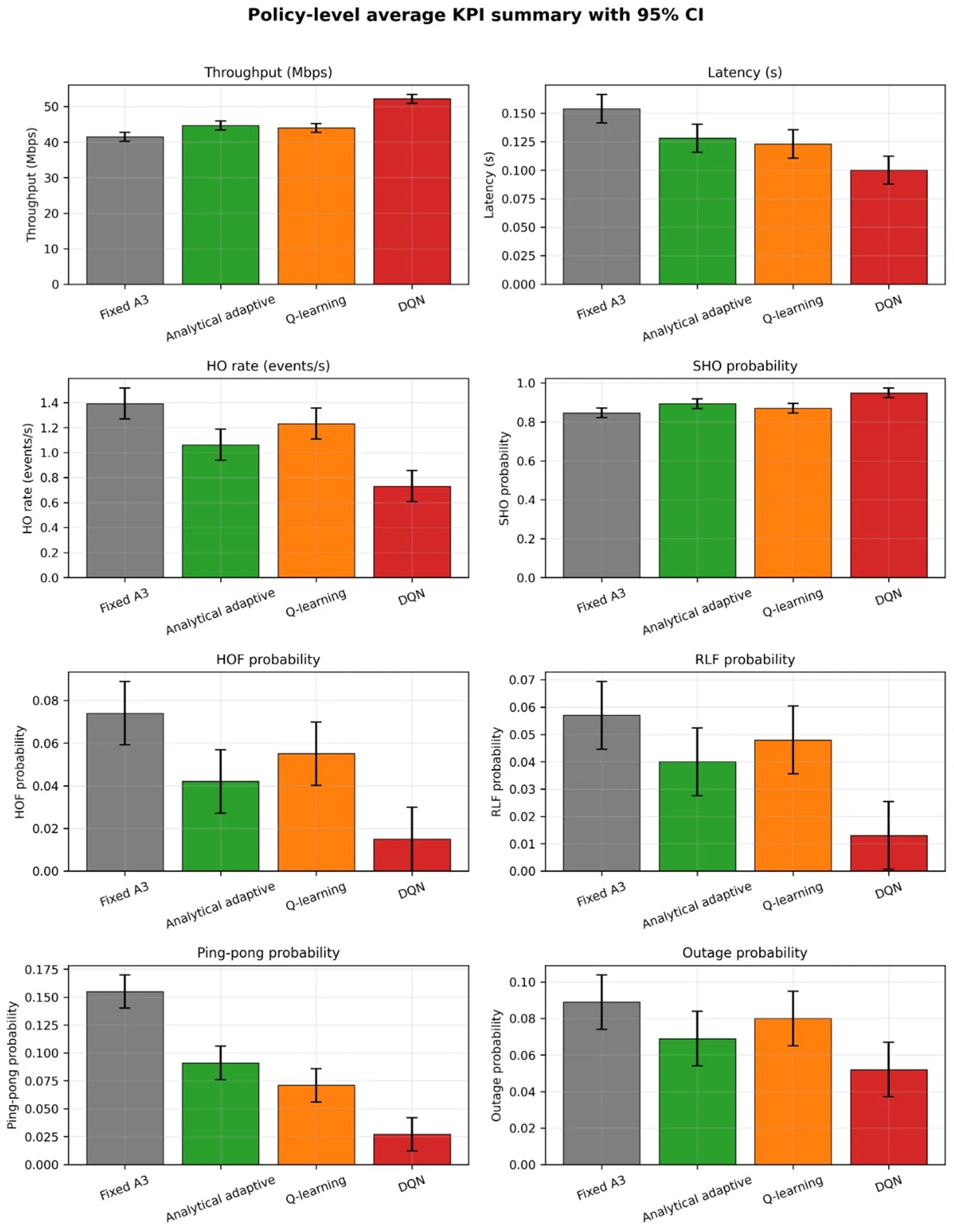
Policy-level average KPI summary with 95% confidence intervals across all tested velocities.

Fig 15 offers a normalized comparison of KPI gains or reductions between the proposed DQN and recent ML/DRL-based handover-optimization studies. The values are reported relative to each study’s fixed-A3 or existing-method baseline; therefore, the comparison is intended for relative trend benchmarking rather than absolute reproduction. The proposed DQN follows the same improvement trend reported in recent mobility-optimization literature while retaining the standard Event A3, FSM, and RLM handover framework [50,51]. Because the studies differ in deployment density, traffic model, propagation assumptions, and KPI definitions, the figure is used as a state-of-the-art contextual comparison rather than as a direct statistical ranking.

**Fig 15:**
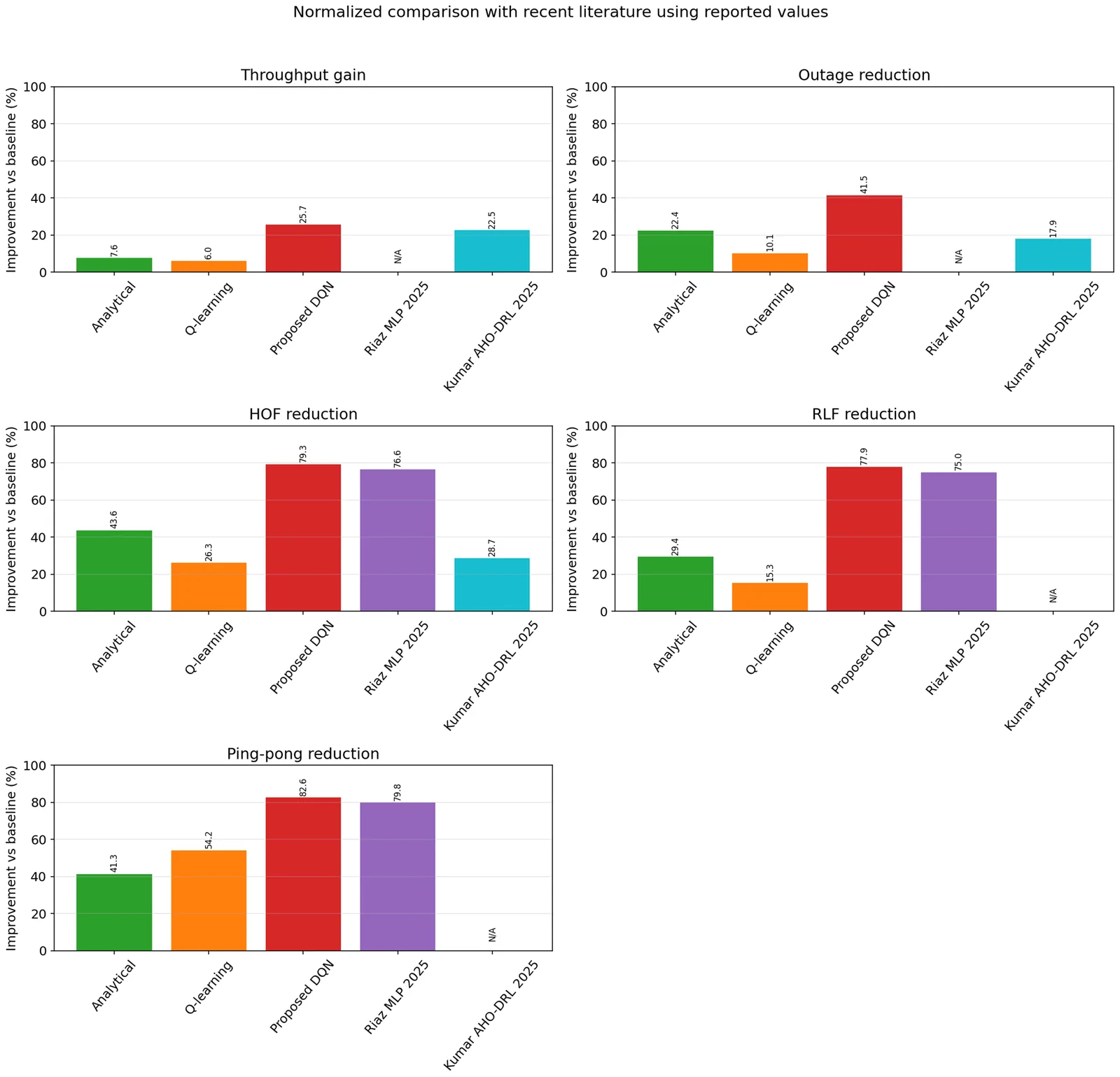
Normalized relative KPI gain/reduction comparison of the proposed DQN with recent ML/DRL-based handover-optimization studies.

### RSRP and SINR characterization

Fig 16 describes the fixed A3 baseline with serving cell RSRP and SINR CDFs. The CDFs move to the left as the velocity rises. This is why the fixed baseline exhibits the largest increase in outage, HOF and RLF at high-velocity mobility.

**Fig 16:**
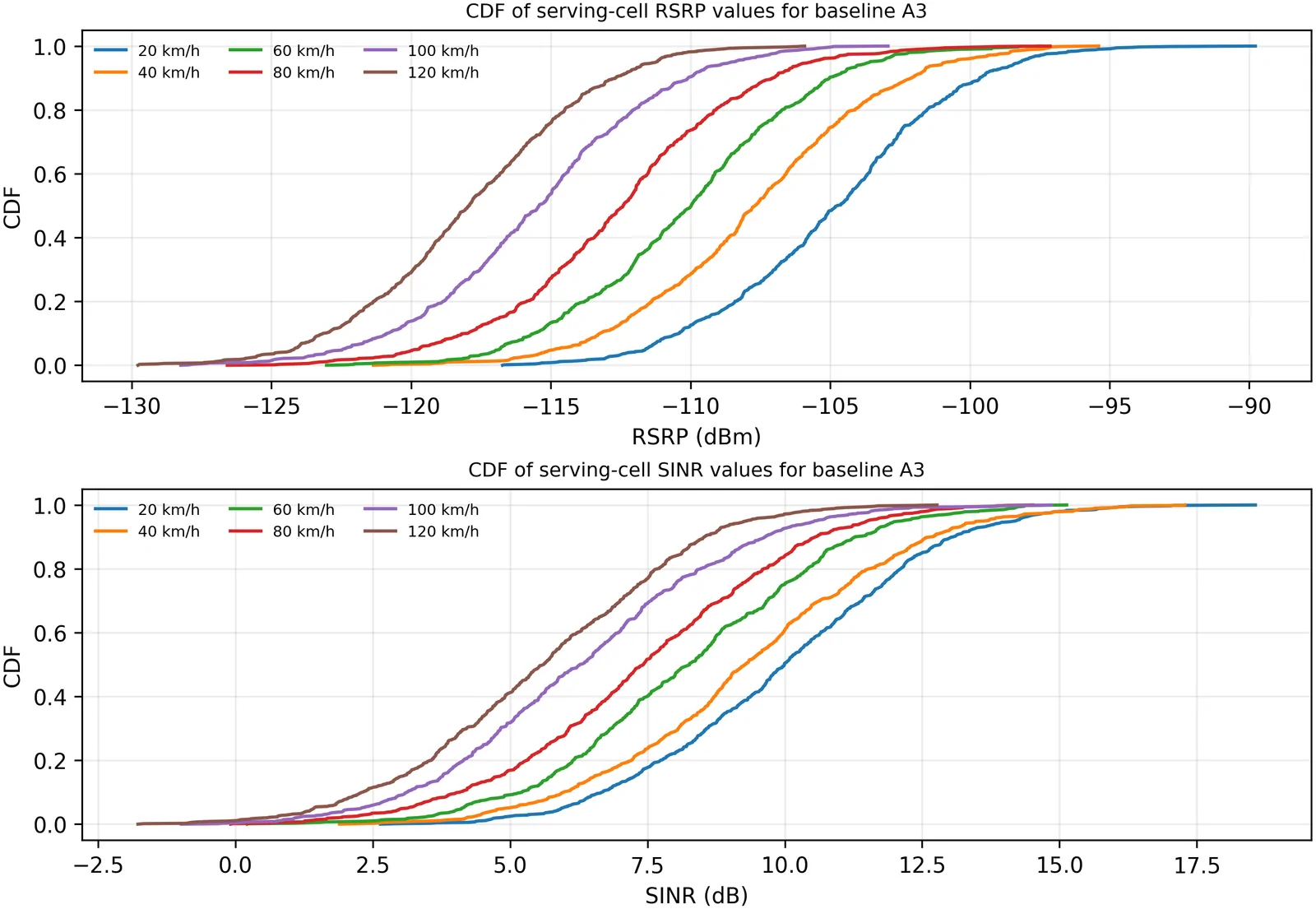
Baseline A3 serving-cell RSRP and SINR CDFs across velocity.

Figs 17 and 18 show the serving-cell RSRP and SINR distributions for all methods at different UE velocities. The DQN curves remain more favourable than those of the other methods, suggesting that its HOM–TTT actions help sustain better serving-cell conditions during mobility. The analytical controller exhibits stable performance because of its mobility-state- and handover-direction-aware decision rules, while the tuned Q-learning controller improves over fixed A3 by selecting more suitable HOM–TTT actions. Q-learning remains inferior to DQN because it uses a tabular state representation, whereas DQN has a more expressive representation that can learn stable action selections across measurement intervals.

**Fig 17:**
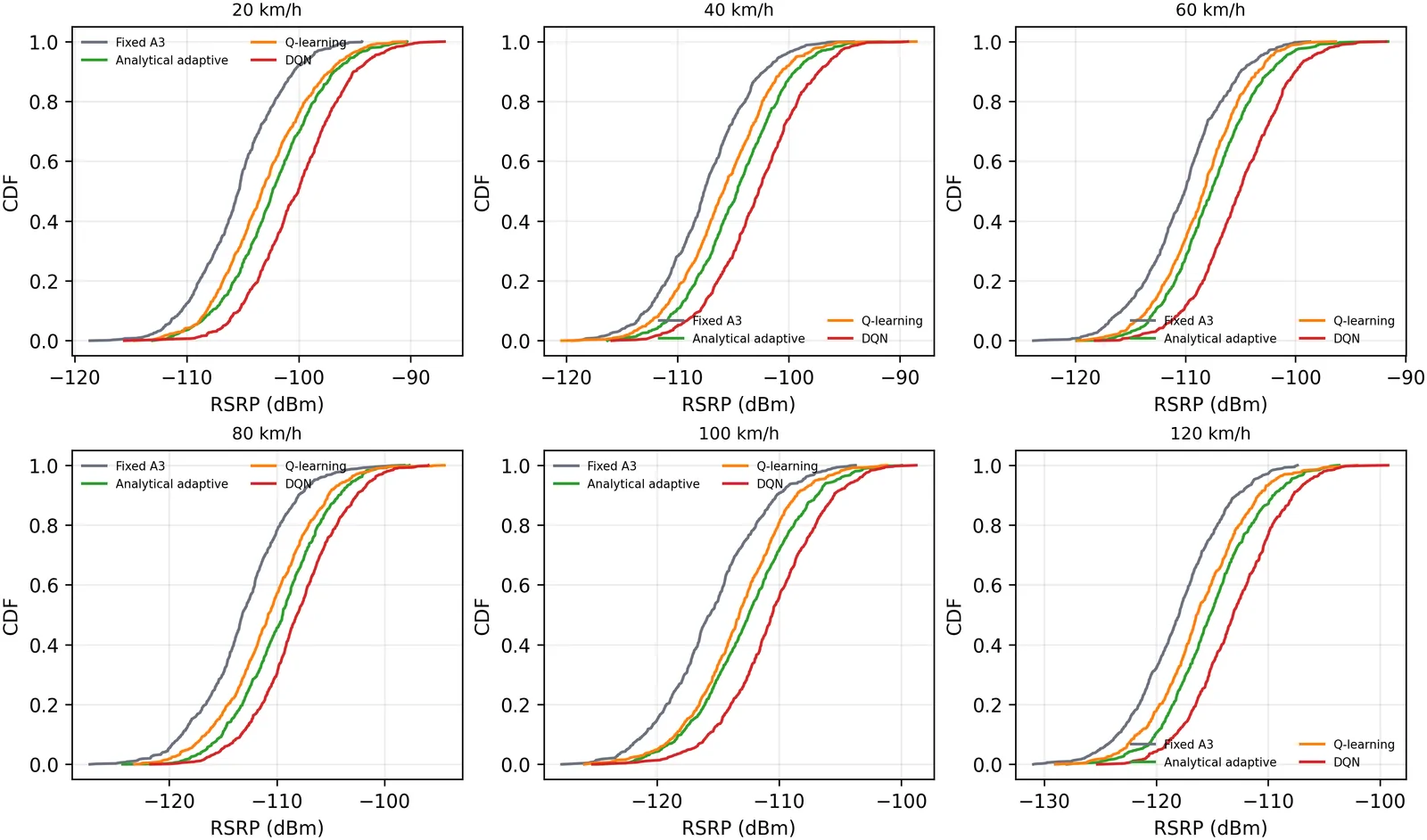
RSRP CDF comparison of all methods for each tested velocity.

**Fig 18:**
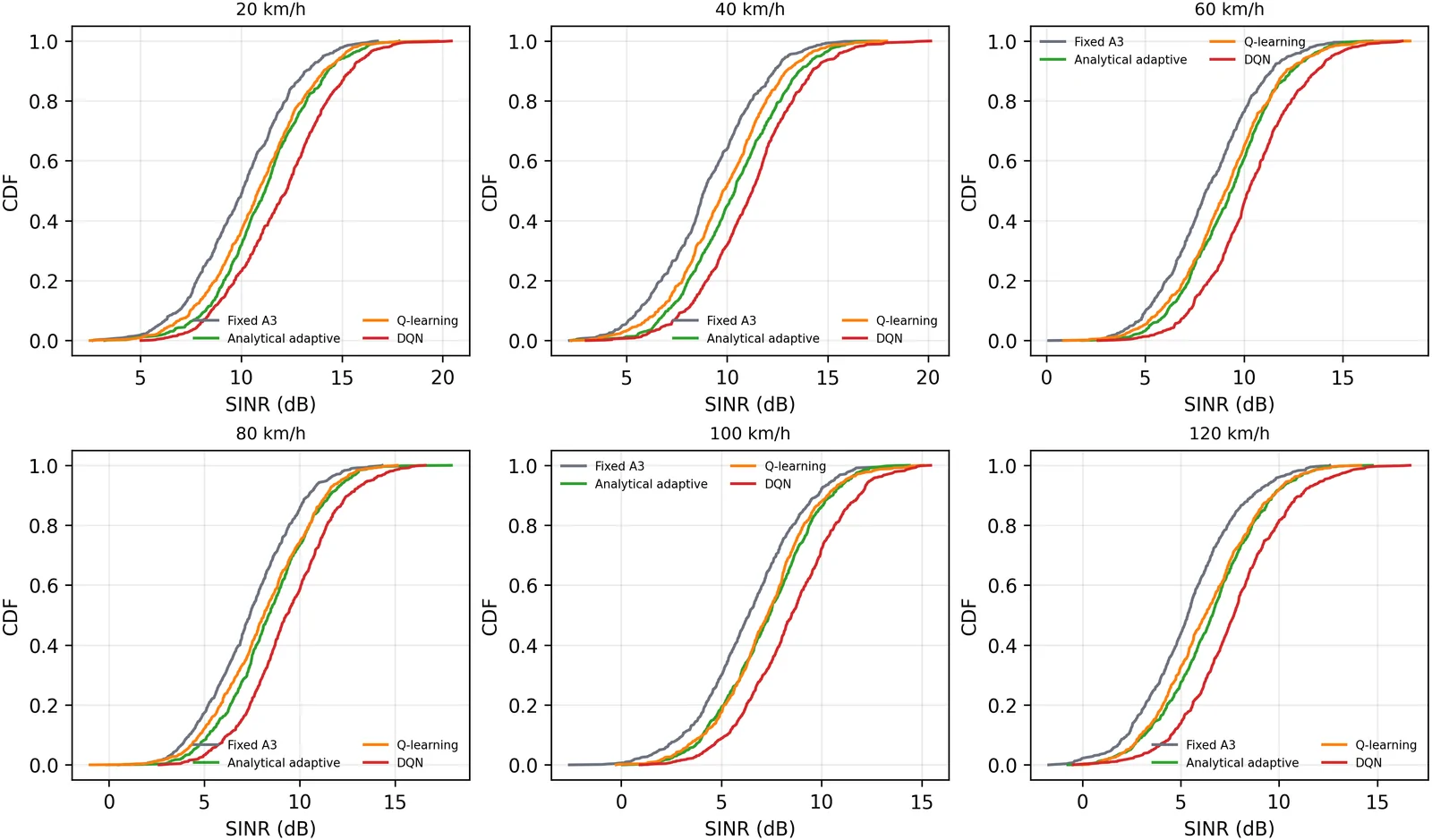
SINR CDF comparison of all methods for each tested velocity.

## Real-world applicability, limitations, and reproducibility

The proposed framework supports practical 5G mobility management because it retains the Event A3 trigger, HOM, TTT, FSM, and RLM concepts. In deployment, the analytical controller could operate as a lightweight self-optimization rule, while Q-learning and DQN could be trained offline and used as lookup or inference modules in a centralized SON or RAN-intelligence controller.

Although the proposed controllers are currently evaluated through simulation, related experimental studies demonstrate that intelligent and measurement-based handover mechanisms can be implemented in practical protocol-stack environments. Akdogan et al. implemented the FLeXHO mobility-optimisation framework on an OpenAirInterface-based end-to-end testbed and reported a 94.5% handover-success rate [54]. The proposed DQN achieves a mean handover-success probability of 0.950, which follows a similar high-reliability trend. However, this is only a contextual comparison because the topology, traffic conditions, mobility model, controller architecture and KPI definitions differ between the studies. The present DQN also preserves the standard Event A3, FSM and RLM framework while adapting HOM and TTT.

Bertolini and Maman further demonstrated end-to-end X2 handover using OpenAirInterface, USRP devices and a channel emulator, including measurements of throughput and handover preparation, execution and completion time [55]. A similar platform could validate the proposed framework by retaining the standard measurement-report and Event A3 procedures while allowing the analytical, Q-learning or DQN controller to select HOM and TTT.

The main limitations of the present study are the three-run evaluation, simulation-only validation, the single-UE scenario, the absence of hardware/testbed validation, and the qualitative rather than quantitative reward-weight sensitivity analysis.

For reproducibility,the manuscript reports the documented simulation parameters, KPI definitions, controller state variables, action sets reward expressions, DQN hyperparameters, seed-level summary values, and statistical summaries. S1 File provides implementation-level pseudocode for all four controllers, allowing the documented workflow and controller logic to be independently re-implemented.

## Conclusion

This paper presented a mobility-state- and handover-direction-aware HCP optimization framework for 5G heterogeneous networks under height-aware 3D geometry, a regular sectorized hexagonal topology, and 3GPP TR 38.901 UMa/UMi propagation. The framework preserves the standard Event A3 trigger, finite-state handover execution, and radio-link monitoring of TS 38.331 while comparing fixed A3, an analytical adaptive controller, tuned tabular Q-learning, and an improved DQN under identical deployment, propagation, mobility, and KPI definitions. Fixed A3 is used as the baseline reference because it represents the non-adaptive HOM–TTT configuration against which the adaptive and learning-based policies are evaluated.

The results confirm that fixed A3 degrades with increasing UE velocity because one HOM–TTT pair cannot jointly control ping-pong, late handover, HOF, RLF, outage, and latency across the 20–120 km/h range. The HOM and TTT sensitivity analysis further shows the main trade-off: larger HOM or TTT values reduce unnecessary triggering but delay handover, increasing HOF, RLF, outage, and latency. This supports the need for adaptive HOM–TTT selection using both mobility state and handover direction.

Relative to fixed A3, the analytical controller increased throughput by 7.6%, reduced latency by 16.9%, reduced handover rate by 23.7%, increased SHO probability by 4.8 percentage points, and reduced HOF, RLF, ping-pong, and outage probabilities by 43.2%, 29.8%, 41.3%, and 22.5%, respectively. These gains show that a standards-compatible and interpretable controller can improve mobility robustness without training overhead. Tuned Q-learning also improved over fixed A3, increasing throughput by 6.0%, reducing latency by 20.1%, reducing handover rate by 11.5%, increasing SHO probability by 2.5 percentage points, and reducing HOF, RLF, ping-pong, and outage by 25.7%, 15.8%, 54.2%, and 10.1%, respectively. However, Q-learning does not dominate every adaptive method because its tabular state representation discretizes the continuous interaction among speed, SINR, handover direction, and handover history.

Under the tested simulation conditions, DQN achieved the best observed mean multi-KPI performance among the four evaluated controllers: throughput (52.17 Mbps), latency (0.100 s), handover rate (0.732 events/s), successful handover probability (0.950), HOF (0.015), RLF (0.013), ping-pong probability (0.027), and outage probability (0.052

These results are based on the current simulation study. In the future, more independent runs, multi-UE traffic, recorded network traces, and hardware or software-defined-radio testbeds should be used.

## Supporting information

S1 File Controller implementation pseudocode. Implementation-level pseudocode for the fixed A3 baseline, the analytical controller, the tabular Q-learning controller, and the improved DQN controller, including the shared simulation modules.
(PDF)
