# Supplementary material for: Mobility-state- and handover-direction-aware analytical and machine learning-based HCP optimization in 5G heterogeneous networks
Source: PLoS One. 2026 Nov 12;21(11):e0362310. doi: 10.1371/journal.pone.0362310 (PMC13759111; doi:10.1371/journal.pone.0362310)
Supplement: Supplementary file 1 [file pone.0362310.s001.pdf]

# S1 File. Controller implementation pseudocode.

Supporting information for: *Mobility-State- and Handover-Direction-Aware Analytical and Machine Learning-Based HCP Optimization in 5G Heterogeneous Networks.*

This file presents detailed implementation-level pseudocode for the four handover-control policies evaluated in this study: the fixed A3 baseline, the mobility-state- and handover-direction-aware analytical controller, the tabular Q-learning controller, and the improved DQN controller. It is organized into shared pseudocode modules and controller-specific pseudocode algorithms. The shared modules define common operations, including initialization, measurement filtering, target selection, Event A3/FSM execution, radio-link monitoring, KPI computation, and evaluation. The controller-specific algorithms show how each policy selects HOM and TTT within the same standard handover framework. Equation numbers refer to the main manuscript.

## Shared Implementation Pseudocode Modules

The following common routines are used identically by the fixed A3 baseline, analytical adaptive controller, tabular Q-learning controller, and improved DQN controller.

**Implementation Module M1. Common data structures and initialization**

**Require:** Network topology $\mathscr{B} = \mathscr{M} \cup \mathscr{S}$, UE trajectory, carrier frequency $f_c$, simulation duration $T_{\text{sim}}$, measurement interval $\Delta t$, simulation seed, transmit powers, antenna gains, noise power, path-loss parameters, and mobility parameters
**Ensure:** Initialized simulation state, counters, timers, and handover history

Set measurement interval $\Delta t = 0.04$ s.
Set total number of measurement steps $K = T_{\text{sim}}/\Delta t$.
Set velocity limits $v_{\min} = 20$ km/h and $v_{\max} = 120$ km/h.
Set layer-3 IIR filter coefficient $\beta = 0.2$.
Set outage and RLM thresholds $\gamma_{\text{th}} = -8$ dB, $Q_{\text{out}} = -8$ dB, and $Q_{\text{in}} = -6$ dB.
Set RLM timers and counters $T_{310} = 200$ ms, $T_{311} = 1000$ ms, $N_{310} = 1$, and $N_{311} = 1$.
Set ping-pong minimum time-of-stay $T_{\text{MTS}} = 1.0$ s.
Set handover preparation and execution delays $T_{\text{prep}} = 0.02$ s and $T_{\text{exec}} = 0.01$ s.
Define the valid 3GPP TTT set using Eq. (54).
Select initial serving cell $s_{\text{cell}}$ from $\mathscr{B}$.
Initialize filtered RSRP values $\bar{R}_i[0] \leftarrow 0$ for all $i \in \mathscr{B}$.
Initialize Event A3 state: $A3_{\text{active}} \leftarrow$ FALSE and $t_{A3}^{\text{start}} \leftarrow 0$.
Initialize RLM counters: $N_{310}^{\text{cnt}} \leftarrow 0$ and $N_{311}^{\text{cnt}} \leftarrow 0$.
Initialize $T_{310}$ state: $T_{310}^{\text{running}} \leftarrow$ FALSE and $t_{310}^{\text{start}} \leftarrow 0$.
Initialize handover history $\mathscr{H}_{\text{HO}} \leftarrow [\,]$.
Initialize KPI counters $N_{\text{att}}$, $N_{\text{SHO}}$, $N_{\text{HOF}}$, $N_{\text{RLF}}$, $N_{\text{PP}}$, and $N_{\text{out}}$ to zero.
Initialize accumulated throughput $\text{Sum}_{\text{tput}} \leftarrow 0$ and latency $\text{Sum}_{\text{lat}} \leftarrow 0$.
Initialize direction-specific handover counters $N_{m\to s}$, $N_{s\to m}$, and $N_{\text{same}}$ to zero.
**return** initialized simulation state.

**Implementation Module M2. Measurement, filtering, and target selection**

**Require:** Measurement step $k$, UE position $\mathbf{q}_{\text{UE}}[k]$, serving cell $s_{\text{cell}}$, base-station set $\mathscr{B}$, and simulation seed
**Ensure:** Target candidate $j_{\text{cand}}$, signal gap $\Delta_k$, serving SINR $\gamma_s[k]$, throughput $C_k$, and handover direction $d[k]$

**for** each base station $i \in \mathscr{B}$ **do**
  Compute 2D and 3D UE–BS distance using Eqs. (42)–(56) where applicable.
  Compute tier-dependent 3GPP TR 38.901 UMa/UMi path loss.
  Compute received power $R_i[k]$ including transmit power, antenna gain, path loss, and shadowing.
  Update filtered RSRP $\bar{R}_i[k] \leftarrow (1-\beta)\bar{R}_i[k-1] + \beta R_i[k]$.
**end for**
Select target candidate $j_{\text{cand}} \leftarrow \arg\max_{i \in \mathscr{B} \setminus s_{\text{cell}}} \bar{R}_i[k]$.
Compute signal gap $\Delta_k \leftarrow \bar{R}_{j_{\text{cand}}}[k] - \bar{R}_{s_{\text{cell}}}[k]$.
Compute serving SINR $\gamma_s[k]$ using the serving signal, interference, noise, and bandwidth.
Compute instantaneous throughput $C_k$.
**if** $s_{\text{cell}} \in \mathscr{M}$ and $j_{\text{cand}} \in \mathscr{S}$ **then**
  Set handover direction $d[k] \leftarrow m \to s$.

**else if** $s_{\text{cell}} \in \mathscr{S}$ and $j_{\text{cand}} \in \mathscr{M}$ **then**
Set handover direction $d[k] \leftarrow s \rightarrow m$.
**else**
Set handover direction $d[k] \leftarrow$ same-tier.
**end if**
**return** $j_{\text{cand}}$, $\Delta_k$, $\gamma_s[k]$, $C_k$, and $d[k]$.

**Implementation Module M3. Event A3 evaluation and handover execution**

**Require:** Measurement step $k$, selected HOM $H_{\text{sel}}$, selected TTT $T_{\text{sel}}$, target candidate $j_{\text{cand}}$, and signal gap $\Delta_k$
**Ensure:** Updated Event A3 state, serving cell, handover history, and handover KPI counters

**if** $\Delta_k \geq H_{\text{sel}}$ **then**
**if** $A3_{\text{active}} = \text{FALSE}$ **then**
Start Event A3 holding condition.
$A3_{\text{active}} \leftarrow \text{TRUE}$.
$t_{A3}^{\text{start}} \leftarrow k\Delta t$.
**else if** $k\Delta t - t_{A3}^{\text{start}} \geq T_{\text{sel}}$ **then**
TTT is satisfied; initiate handover attempt.
$N_{\text{att}} \leftarrow N_{\text{att}} + 1$.
Compute latency $L_h \leftarrow T_{\text{sel}} + T_{\text{prep}} + T_{\text{exec}}$.
**if** $\gamma_s[k] > Q_{\text{in}}$ and $T_{310}^{\text{running}} = \text{FALSE}$ **then**
Declare successful handover.
$N_{\text{SHO}} \leftarrow N_{\text{SHO}} + 1$.
$\text{Sum}_{\text{lat}} \leftarrow \text{Sum}_{\text{lat}} + L_h$.
**if** handover returns to the previous serving cell within $T_{\text{MTS}}$ **then**
$N_{\text{PP}} \leftarrow N_{\text{PP}} + 1$.
**end if**
Append $(j_{\text{cand}}, k\Delta t)$ to $\mathscr{H}_{\text{HO}}$.
**if** $s_{\text{cell}} \in \mathscr{M}$ and $j_{\text{cand}} \in \mathscr{S}$ **then**
$N_{m\rightarrow s} \leftarrow N_{m\rightarrow s} + 1$.
**else if** $s_{\text{cell}} \in \mathscr{S}$ and $j_{\text{cand}} \in \mathscr{M}$ **then**
$N_{s\rightarrow m} \leftarrow N_{s\rightarrow m} + 1$.
**else**
$N_{\text{same}} \leftarrow N_{\text{same}} + 1$.
**end if**
Update serving cell $s_{\text{cell}} \leftarrow j_{\text{cand}}$.
**else**
Declare handover failure.
$N_{\text{HOF}} \leftarrow N_{\text{HOF}} + 1$.
**end if**
Reset Event A3 timer.
$A3_{\text{active}} \leftarrow \text{FALSE}$.
$t_{A3}^{\text{start}} \leftarrow 0$.
**end if**
**else**
Event A3 condition is broken before TTT expiry.
$A3_{\text{active}} \leftarrow \text{FALSE}$.
$t_{A3}^{\text{start}} \leftarrow 0$.
**end if**
**return** updated handover state and counters.

**Implementation Module M4. RLM and outage update**

**Require:** Measurement step $k$, serving SINR $\gamma_s[k]$, $Q_{\text{out}}$, $Q_{\text{in}}$, $N_{310}$, $N_{311}$, and $T_{310}$
**Ensure:** Updated RLM state, RLF counter, outage counter, and serving cell after re-establishment

**if** $\gamma_s[k] < Q_{\text{out}}$ **then**
$N_{310}^{\text{cnt}} \leftarrow N_{310}^{\text{cnt}} + 1$.
$N_{311}^{\text{cnt}} \leftarrow 0$.

**if** $N_{310}^{\text{cnt}} \geq N_{310}$ and $T_{310}^{\text{running}} = \text{FALSE}$ **then**
Start $T_{310}$.
$T_{310}^{\text{running}} \leftarrow \text{TRUE}$.
$t_{310}^{\text{start}} \leftarrow k\Delta t$.
**end if**
**else**
$N_{311}^{\text{cnt}} \leftarrow N_{311}^{\text{cnt}} + 1$.
**if** $N_{311}^{\text{cnt}} \geq N_{311}$ **then**
Stop $T_{310}$ and reset RLM counters.
$T_{310}^{\text{running}} \leftarrow \text{FALSE}$.
$N_{310}^{\text{cnt}} \leftarrow 0$ and $N_{311}^{\text{cnt}} \leftarrow 0$.
**end if**
**end if**
**if** $T_{310}^{\text{running}} = \text{TRUE}$ and $k\Delta t - t_{310}^{\text{start}} \geq T_{310}$ **then**
Declare radio-link failure.
$N_{\text{RLF}} \leftarrow N_{\text{RLF}} + 1$.
Stop $T_{310}$ and reset RLM counters.
Select strongest available cell $s_{\text{cell}} \leftarrow \arg\max_{i \in \mathscr{B}} \bar{R}_i[k]$.
Reset Event A3 state after re-establishment.
**end if**
**if** $\gamma_s[k] < \gamma_{\text{th}}$ **then**
Count outage sample $N_{\text{out}} \leftarrow N_{\text{out}} + 1$.
**end if**
**return** updated RLM state, RLF counter, and outage counter.

**Implementation Module M5. KPI computation**

**Require:** KPI counters, accumulated throughput, accumulated latency, total steps $K$, and simulation duration $T_{\text{sim}}$
**Ensure:** Final policy-level KPI values
Compute average throughput $\bar{C} \leftarrow \text{Sum}_{\text{tput}}/K$.
Compute average handover latency $\bar{L}_{\text{HO}} \leftarrow \text{Sum}_{\text{lat}}/\max(1, N_{\text{SHO}})$.
Compute handover rate $R_{\text{HO}} \leftarrow N_{\text{att}}/T_{\text{sim}}$.
Compute successful handover probability $P_{\text{SHO}} \leftarrow N_{\text{SHO}}/\max(1, N_{\text{att}})$.
Compute handover failure probability $P_{\text{HOF}} \leftarrow N_{\text{HOF}}/\max(1, N_{\text{att}})$.
Compute radio-link failure probability $P_{\text{RLF}} \leftarrow N_{\text{RLF}}/\max(1, K)$.
Compute ping-pong probability $P_{\text{PP}} \leftarrow N_{\text{PP}}/\max(1, N_{\text{SHO}})$.
Compute outage probability $P_{\text{out}} \leftarrow N_{\text{out}}/K$.
Compute excessive handover count $N_{\text{EHO}} \leftarrow N_{\text{att}} - N_{\text{SHO}}$.
Compute excessive handover probability $P_{\text{EHO}} \leftarrow N_{\text{EHO}}/\max(1, N_{\text{att}})$.
Compute direction-specific handover probabilities $P_{m \to s}$, $P_{s \to m}$, and $P_{\text{same}}$.
Compute direction-specific handover rates $R_{m \to s}$, $R_{s \to m}$, and $R_{\text{same}}$.
**return** all final KPI values.

## Controller-Specific Pseudocode Algorithms

**Algorithm A1. Fixed A3 baseline controller**

**Require:** Fixed HOM $H_{\text{fixed}} = 3.0$ dB, fixed TTT $T_{\text{fixed}} = 160$ ms, network topology, UE trajectory, and simulation seed
**Ensure:** Final fixed-A3 KPI values
Call Implementation Module M1 for common initialization.
**for** each measurement step $k = 0, 1, \ldots, K-1$ **do**
Obtain $(j_{\text{cand}}, \Delta_k, \gamma_s[k], C_k, d[k])$ using Implementation Module M2.
Accumulate throughput $\text{Sum}_{\text{tput}} \leftarrow \text{Sum}_{\text{tput}} + C_k \Delta t$.
Update RLM and outage using Implementation Module M4.
Select $H_{\text{sel}} \leftarrow H_{\text{fixed}}$ and $T_{\text{sel}} \leftarrow T_{\text{fixed}}$.
Apply Event A3/FSM handover using Implementation Module M3.
Update UE position and heading.

**end for**
**return** final KPIs using Implementation Module M5.

**Algorithm A2. Mobility-state- and handover-direction-aware analytical controller**

**Require:** Direction-specific HOM and TTT bounds, $v_{\min}$, $v_{\max}$, valid 3GPP TTT set $\mathscr{T}_{3GPP}$, network topology, UE trajectory, and simulation seed
**Ensure:** Final analytical-controller KPI values
Call Implementation Module M1 for common initialization.
Set HOM bounds $H^L_{m\to s} = 2.0$, $H^H_{m\to s} = 5.0$, $H^L_{s\to m} = 1.0$, $H^H_{s\to m} = 3.5$, and $H_0 = 3.0$ dB.
Set TTT bounds $T^L_{m\to s} = 160$, $T^H_{m\to s} = 480$, $T^L_{s\to m} = 64$, $T^H_{s\to m} = 256$, and $T_0 = 160$ ms.
**for** each measurement step $k = 0,1,\dots,K-1$ **do**
    Obtain $(j_{\text{cand}}, \Delta_k, \gamma_s[k], C_k, d[k])$ using Implementation Module M2.
    Accumulate throughput $\text{Sum}_{\text{tput}} \leftarrow \text{Sum}_{\text{tput}} + C_k \Delta t$.
    Update RLM and outage using Implementation Module M4.
    Compute normalized mobility state $\nu[k] \leftarrow \text{clip}((v[k] - v_{\min})/(v_{\max} - v_{\min}), 0, 1)$.
    **if** $d[k] = m \to s$ **then**
        $H_{\text{sel}} \leftarrow H^L_{m\to s} + \nu[k](H^H_{m\to s} - H^L_{m\to s})$.
        $T_{\text{cont}} \leftarrow T^L_{m\to s} + \nu[k](T^H_{m\to s} - T^L_{m\to s})$.
    **else if** $d[k] = s \to m$ **then**
        $H_{\text{sel}} \leftarrow H^H_{s\to m} - \nu[k](H^H_{s\to m} - H^L_{s\to m})$.
        $T_{\text{cont}} \leftarrow T^H_{s\to m} - \nu[k](T^H_{s\to m} - T^L_{s\to m})$.
    **else**
        $H_{\text{sel}} \leftarrow H_0$ and $T_{\text{cont}} \leftarrow T_0$.
    **end if**
    Quantize $T_{\text{cont}}$ to the nearest value in $\mathscr{T}_{3GPP}$ and assign it to $T_{\text{sel}}$.
    Apply Event A3/FSM handover using Implementation Module M3.
    Update UE position and heading.
**end for**
**return** final KPIs using Implementation Module M5.

**Implementation Module M6. Tabular Q-learning setup**

**Require:** Learning rate $\alpha = 0.1$, discount factor $\gamma = 0.97$, $\varepsilon$-greedy schedule, state bins, HOM–TTT actions, and 300 training episodes
**Ensure:** Initialized Q-table and discrete action space
Define compact Q-learning state $s_k = (v[k], \Delta_k, \gamma_s[k], d[k], t_{\text{sinceSHO}}[k])$.
Discretize speed, signal gap, SINR, handover direction, and time since last successful handover.
Use state-bin structure $5\times5\times4\times3\times4 = 1200$ states.
Define HOM action set $\mathscr{H} = \{1.0, 2.0, 2.5, 3.0, 3.5, 4.0, 5.0\}$ dB.
Define TTT action set $\mathscr{T} = \{40, 80, 160, 240, 320\}$ ms.
Define action space $\mathscr{A} = \mathscr{H} \times \mathscr{T}$ with $|\mathscr{A}| = 35$ actions.
Initialize Q-table $Q(s,a) \leftarrow 0$ for all $s \in \mathscr{S}$ and $a \in \mathscr{A}$.
Initialize $\varepsilon \leftarrow 1.0$ and $\varepsilon_{\min} \leftarrow 0.05$.
**return** initialized Q-learning state and action structures.

**Algorithm A3. Tabular Q-learning-based HCP optimization controller**

**Require:** Q-table $Q(s,a)$, action space $\mathscr{A}$, $\alpha$, $\gamma$, $\varepsilon$ schedule, and training episodes
**Ensure:** Trained Q-learning policy $\pi_Q(s)$
Call Implementation Module M6 to initialize the Q-table, discrete state bins, and HOM–TTT action space.
**for** each training episode $e = 1,2,\dots,300$ **do**
    Reset UE position, serving cell, Event A3 state, RLM timers, handover history, and KPI counters.
    Observe and discretize initial state $s_k$.
    **for** each measurement step $k = 0,1,\dots,K-1$ **do**
        Select action $a_k$ using $\varepsilon$-greedy exploration.
        Decode $a_k$ into selected HOM $H_{\text{sel}}$ and TTT $T_{\text{sel}}$.
        Obtain $(j_{\text{cand}}, \Delta_k, \gamma_s[k], C_k, d[k])$ using Implementation Module M2.
        Store previous KPI counters for event-indicator calculation.

Update RLM and outage using Implementation Module M4.
Apply Event A3/FSM handover using Implementation Module M3.
Compute event indicators $I_{\text{succ}}$, $I_{\text{HOF}}$, $I_{\text{RLF}}$, $I_{\text{PP}}$, $I_{\text{out}}$, and $I_{\text{EHO}}$.
Normalize throughput $\tilde{C}_k$ and handover latency $\tilde{L}_k$.
Compute Q-learning reward $r_k^Q$ using Eq. (57).
Update UE position and heading.
Observe and discretize next state $s_{k+1}$.
Update Q-table:

$$Q(s_k, a_k) \leftarrow Q(s_k, a_k) + \alpha \left[ r_k^Q + \gamma \max_{a'} Q(s_{k+1}, a') - Q(s_k, a_k) \right].$$

Set $s_k \leftarrow s_{k+1}$.
**end for**
Decay exploration $\varepsilon \leftarrow \max(0.05, \varepsilon - (1.0 - 0.05)/300)$.
**end for**
Define greedy evaluation policy $\pi_Q(s) = \arg\max_{a \in \mathscr{A}} Q(s, a)$.
**return** trained Q-learning policy.

**Implementation Module M7. DQN setup**

**Require:** Learning rate $8 \times 10^{-4}$, discount factor $\gamma = 0.97$, replay-buffer capacity 20,000, mini-batch size 32, target-network update period 8 episodes, and 300 episodes
**Ensure:** Initialized online network, target network, replay buffer, and action space
Define normalized DQN state vector $s_k = [s_1, s_2, s_3, s_4, s_5, s_6, s_7]$.
Let $s_1$ represent normalized speed, $s_2$ filtered signal gap, $s_3$ serving SINR, $s_4$ handover direction, $s_5$ time since last successful handover, $s_6$ signal-gap derivative, and $s_7$ serving-tier indicator.
Use the same 35 HOM–TTT actions as the Q-learning controller.
Define DQN architecture $7 \to 32 \to 32 \to 35$.
Use ReLU activation in hidden layers and linear Q-values in the output layer.
Initialize online network $Q_\theta$ and target network $Q_{\theta^-}$.
Set target-network parameters $\theta^- \leftarrow \theta$.
Initialize replay memory $\mathscr{D} \leftarrow \emptyset$ with capacity 20,000.
Initialize previous action $H_{\text{prev}} \leftarrow 3.0$ dB and $T_{\text{prev}} \leftarrow 160$ ms.
Initialize exploration $\varepsilon \leftarrow 1.0$.
**return** initialized DQN structures.

**Algorithm A4. Improved DQN-based HCP optimization controller**

**Require:** Online network $Q_\theta$, target network $Q_{\theta^-}$, replay memory $\mathscr{D}$, action space, and DQN hyperparameters
**Ensure:** Trained DQN policy $\pi_{\text{DQN}}(s)$
Call Implementation Module M7 to initialize the online network, target network, replay memory, and HOM–TTT action space.
**for** each training episode $e = 1, 2, \ldots, 300$ **do**
Reset common environment, RLM state, Event A3 state, handover history, and KPI counters.
Observe normalized initial state $s_k$.
**for** each measurement step $k = 0, 1, \ldots, K-1$ **do**
Select action $a_k$ using $\varepsilon$-greedy policy.
Decode $a_k$ into selected HOM $H_{\text{sel}}$ and TTT $T_{\text{sel}}$.
Obtain $(j_{\text{cand}}, \Delta_k, \gamma_s[k], C_k, d[k])$ using Implementation Module M2.
Store previous KPI counters for event-indicator calculation.
Update RLM and outage using Implementation Module M4.
Apply Event A3/FSM handover using Implementation Module M3.
Compute event indicators $I_{\text{succ}}$, $I_{\text{HOF}}$, $I_{\text{RLF}}$, $I_{\text{PP}}$, $I_{\text{out}}$, and $I_{\text{EHO}}$.
Compute base reward using Eq. (57).
Compute smoothness penalty from changes in HOM and TTT.
Compute direction-consistency reward using the handover direction and selected HOM.
Compute total DQN reward $r_k^{DQN}$ using Eq. (58).
Update successful-handover time, UE position, heading, and previous action.

Observe next normalized state $s_{k+1}$.
Store transition $(s_k, a_k, r_k^{DQN}, s_{k+1}, d_k^{\text{term}})$ in replay memory $\mathscr{D}$.
If replay memory exceeds capacity, remove the oldest transition.
Call Implementation Module M8 for replay update when $|\mathscr{D}| \geq 32$.
Set $s_k \leftarrow s_{k+1}$.
**end for**
**if** $e \bmod 8 = 0$ **then**
Synchronize target network $\theta^- \leftarrow \theta$.
**end if**
Decay exploration $\varepsilon \leftarrow \max(0.05, \varepsilon - (1.0 - 0.05)/300)$.
**end for**
Define greedy evaluation policy $\pi_{\text{DQN}}(s) = \arg\max_a Q_\theta(s,a)$.
**return** trained DQN policy.

**Implementation Module M8. DQN replay-memory update**

**Require:** Replay memory $\mathscr{D}$, mini-batch size 32, online network $Q_\theta$, target network $Q_{\theta^-}$, discount factor $\gamma$, and learning rate $8 \times 10^{-4}$

**Ensure:** Updated online-network parameters $\theta$

Sample mini-batch $\mathscr{B}$ of 32 transitions uniformly from $\mathscr{D}$.
**for** each transition $(s_i, a_i, r_i, s_{i+1}, d_i)$ in $\mathscr{B}$ **do**
**if** $d_i = 1$ **then**
Set target $y_i \leftarrow r_i$.
**else**
Set target $y_i \leftarrow r_i + \gamma \max_{a'} Q_{\theta^-}(s_{i+1}, a')$.
**end if**
**end for**
Compute Bellman loss:

$$\mathscr{L}(\theta) = \frac{1}{32} \sum_{i=1}^{32} \left[ y_i - Q_\theta(s_i, a_i) \right]^2 .$$

Update online network using Adam gradient descent:

$$\theta \leftarrow \theta - 8 \times 10^{-4} \nabla_\theta \mathscr{L}(\theta).$$

**return** updated online-network parameters.

**Implementation Module M9. Controller evaluation**

**Require:** Trained policy $\pi_Q$ or $\pi_{\text{DQN}}$, analytical policy, or fixed-A3 policy

**Ensure:** Final seed-level and policy-level KPI values

**for** each independent simulation seed **do**
Reset the common simulation environment using Implementation Module M1.
**for** each measurement step $k = 0, 1, \ldots, K-1$ **do**
Obtain measurements using Implementation Module M2.
Select HOM–TTT using the chosen controller.
Update RLM and outage using Implementation Module M4.
Apply Event A3/FSM handover using Implementation Module M3.
Update UE position and heading.
**end for**
Compute seed-level KPIs using Implementation Module M5.
**end for**
Compute mean, SD, SE, and 95% confidence interval across the three seeds.
**return** final statistical KPI summary.
