# Supplementary material for: Mobility-state- and handover-direction-aware analytical and machine learning-based HCP optimization in 5G heterogeneous networks
Source: PLoS One. 2026 Nov 12;21(11):e0362310. doi: 10.1371/journal.pone.0362310 (PMC13759111; doi:10.1371/journal.pone.0362310)
Supplement: Supplementary file 2 [file pone.0362310.s003.docx]

**Response to Reviewers**

Manuscript: Mobility-State- and Handover-Direction-Aware Analytical and Machine Learning-Based HCP Optimization in 5G Heterogeneous Networks

Dear Editor and Reviewers,

We sincerely thank the journal Editor and the reviewers for their careful reading of our manuscript and for their constructive comments. We have revised the manuscript to improve clarity, reproducibility, statistical reporting, and the explanation of the analytical, Q-learning, and DQN controllers. The revised manuscript is submitted in both marked-up and clean versions. In the marked-up version, newly added or modified text is shown in blue and deleted text is shown with red strikethrough. The verified locations are based on the marked-up revised manuscript.

# Editorial and PLOS ONE technical requirements

**Editor comment: E1-E5**

**Author response:** In the revised version, all the additional requirements have been addressed and incorporated.

**Editor comment: E6**

Please ensure that you refer to Figures 1 - 7 in your text as, if accepted, production will need this reference to link the reader to the figure.

**Author response:** Figures 1–7 are now explicitly referred to in the manuscript text. The explanatory paragraphs were revised so that each figure is introduced and interpreted in the relevant section.

**Location in Manuscript with Track Changes:** Figure 1: p. 5; Figure 2: p. 6; Figure 3: p. 11; Figure 4: p. 12; Figure 5: pp. 12–13; Figure 6: p. 17; Figure 7: pp. 18–19**.**

**Editor comment: E7**

We note you have included a table to which you do not refer in the text of your manuscript. Please ensure that you refer to Tables 1 - 3 in your text; if accepted, production will need this reference to link the reader to the Table.

**Author response:** Tables 1-3 are now explicitly referred to in the manuscript text. The revised text explains the common simulation-parameter table, the state/action representation table, and the DQN hyperparameter table.

**Location in Manuscript with Track Changes:** Table 1 and its explanatory text: pp. 13-14; Table 2 and its explanatory text: p. 14 and p. 17; Table 3 and its explanatory text: p. 17.

**Editor comment: E8**

If the reviewer comments include a recommendation to cite specific previously published works, please review and evaluate these publications to determine whether they are relevant and should be cited. There is no requirement to cite these works unless the editor has indicated otherwise.

**Author response:** The recommended works were evaluated and incorporated into the related-work discussion where relevant. They are used to contextualize learning-based management in 5G HetNets, vertical handover self-optimization, and SINR/resource-availability issues in heterogeneous deployments.

**Location in Manuscript with Track Changes:** Related-work discussion incorporating the suggested works: p. 3; references [53]-[55]: pp. 40-41.

# Reviewer #1: Comments and responses

## Reviewer #1, Comment 1:

*The manuscript is technically sound. The four controllers are evaluated under identical network geometry, propagation, and KPI definitions, ensuring fair comparison. The conclusions that DQN delivers the best multi-KPI trade-off, Q-learning improves over fixed A3, and the analytical controller is an interpretable baseline are supported by Table 4 and Figures 12–15. However, three concerns need addressing. First, only three random seeds are used without reporting variability measures; standard deviations or confidence intervals should be added to Table 4, particularly for closely performing methods. Second, the single-UE assumption excludes load-dependent interference effects and should be explicitly acknowledged as a limitation. Third, no ablation is provided for the DQN architecture choices; a brief sensitivity discussion on hidden layer size and training episodes is needed. Finally, the conclusion claiming Q-learning outperforms fixed A3 across all KPIs is inconsistent with Figure 12(c) and should be corrected.*

**Author response:** We thank the reviewer for recognizing the technical soundness of the work. We have addressed all four concerns. First, the statistical reporting now includes three seed-level values, mean, SD, SE, and 95% confidence intervals for each policy-level KPI, with a note that overlapping intervals are interpreted cautiously. Second, the single-UE limitation is now explicitly acknowledged, including load-dependent interference, inter-cell scheduling, admission control, and multi-UE resource competition. Third, the DQN section now reports the architecture, hyperparameters, replay buffer, mini-batch size, target-network update, parameter count, and convergence assessment. A component-level ablation interpretation was added for mobility-state awareness, handover-direction awareness, and DQN smoothness terms. Finally, the conclusion was revised so that Q-learning is described as improving over fixed A3 on the main descriptive mobility-robustness metrics, without claiming dominance over every non-fixed method, KPI, or velocity point.

**Location in Manuscript with Track Changes:** Statistical method: p. 14; Table 4 and Tables 4a-4h: pp. 25-26; Figure 14 and uncertainty interpretation: pp. 27-28; DQN architecture/hyperparameters: pp. 17-18; computational complexity: p. 19; ablation interpretation: pp. 20-21 and p. 33; real-world applicability and single-UE limitation: pp. 31-33; revised conclusion: pp. 32-33.

## Reviewer #1, Comment 2:

*The statistical treatment is the main weakness. Results are reported as averages over three seeds with no variability measures, which is insufficient given stochastic shadowing and random mobility. Differences between closely performing methods for example, latency of 0.128 s vs. 0.123 s between the analytical controller and Q-learning may not be statistically significant. The authors should increase seeds and report standard deviations, or justify the current approach. Additionally, the eight reward weights in Equation 57 are stated without justification; the authors should clarify whether they were tuned systematically or empirically, and briefly discuss sensitivity to weight perturbations.*

**Author response:** The revised manuscript now reports the available three independent random seeds explicitly. For each policy-level KPI, Table 4a–4h provides the mean, Seed 1, Seed 2, Seed 3, SD, SE, and t-based 95% confidence interval using n = 3, df = 2, and t0.975,2 = 4.303. The manuscript also states that because the sample size is small, the confidence intervals are descriptive uncertainty measures rather than formal hypothesis tests, and close or overlapping intervals are interpreted cautiously. The reward-weight paragraph was also revised to explain the physical severity ordering: HOF and RLF receive the largest penalties because they interrupt service continuity; outage is penalized next; ping-pong is penalized to discourage instability; and latency/excessive handover receive smaller penalties to avoid an over-conservative policy. The text also clarifies that no independent one-factor-at-a-time weight-perturbation run is claimed.

**Location in Manuscript with Track Changes:** Statistical definitions: p. 14; Table 4 and Tables 4a-4h with note: pp. 25-26; Figure 14 and statistical interpretation: pp. 27-28; reward-weight rationale and sensitivity limitation: pp. 14-15; revised conclusion: pp. 32-33.

## Reviewer #1, Comment 3:

*The authors state all data are within the manuscript, which is acceptable for simulation-based work. However, the simulation code is not shared. Depositing the code in a public repository would substantially improve reproducibility, consistent with PLOS ONE policy. If code sharing is not feasible, a more detailed algorithmic description sufficient for independent replication particularly for DQN training and the analytical controller parameter bounds should be provided.*

**Author response:** We have substantially strengthened the reproducibility material through a detailed algorithmic description. The revised Data Availability Statement reports that all numerical data supporting the findings are provided within the manuscript and supporting information. The manuscript includes the seed-level KPI values, mean values, SD, SE, and 95% confidence intervals, as well as the simulation settings, network configuration, mobility assumptions, state variables, action space, reward expressions, controller settings, and DQN hyperparameters. Appendix-A now provides detailed implementation-level pseudocode for the four handover-control policies for the fixed A3 baseline, analytical controller, Q-learning controller, and DQN-based controller. The detailed pseudocode covers the common simulation workflow, measurement processing, Event A3 triggering, RLM updates, KPI accumulation, analytical HOM–TTT selection, Q-learning update, DQN training loop, replay-memory update, target-network update, and final controller evaluation.

**Location in Manuscript with Track Changes:** Reproducibility discussion: pp. 31-32; Data Availability statement: p. 34; analytical-controller parameter bounds in Appendix A: p. 37; Q-learning setup in Appendix A: p. 37; DQN setup/training and replay update in Appendix A: p. 38; DQN model description in main text: pp. 17-18.

## Reviewer #1, Comment 4:

The manuscript is generally readable and well-structured. However, several issues require correction: (i) "small cells augment capacity and reuse" is an incomplete sentence; (ii) "Figure 10 shows The TTT trade-off" contains an erroneous capital; (iii) "it is more can handle larger" is grammatically incorrect; (iv) the caption of Figure 15 incorrectly describes the figure as showing training reward convergence when it actually presents normalized KPI gain comparisons with recent literature. A careful language revision is recommended. The authors should also include three references that better contextualize their work. Lv, Z., Singh, A.K. and Li, J., 2021. Deep learning for security problems in 5G heterogeneous networks. IEEE Network, 35(2), pp.67-73 — relevant to motivating learning-based management in 5G HetNets. Kiran, K., 2023. 5G heterogeneous network (HetNets): a self-optimization technique for vertical handover management. International Journal of Pervasive Computing and Communications, 19(1), pp.1-22 — directly related to vertical handover self-optimization and should be discussed in related work. Alhulayil, M. and López-Benítez, M., 2019, October. Methods for the allocation of almost blank subframes with fixed duty cycle for improved LTE-U/Wi-Fi coexistence. In 2019 International Conference on Wireless Networks and Mobile Communications (WINCOM) (pp. 1-6). IEEE — ABS coexistence mechanisms affect SINR and resource availability during handover in heterogeneous deployments and are relevant to the broader HetNet context of this work.

**Author response:** The manuscript has been revised for language clarity, and the specific issues identified by the reviewer have been corrected. The incomplete sentence about small cells has been rewritten as a complete sentence. The erroneous capitalization in the Figure 10 discussion has been corrected. The DQN flow explanation has been corrected to state clearly that DQN can handle larger and more continuous state spaces. The caption and discussion of Figure 15 have been corrected to describe normalized relative KPI gain/reduction comparison with recent ML/DRL-based handover-optimization studies. The three suggested references have been evaluated and incorporated into the related-work discussion and reference list.

**Location in Manuscript with Track Changes:** Small-cell sentence: p. 2; Figure 10 discussion: p. 22; corrected DQN wording: pp. 17-19; corrected Figure 15 caption and discussion: p. 29; added related-work discussion: p. 3; added references [53]-[55]: pp. 40-41.

# Reviewer #2: Comments and responses

## Reviewer #2, Comment 1:

The manuscript is of relevance and tackles a critical issue in 5G heterogeneous network mobility management.

**Author response:** We thank the reviewer for this positive assessment.

## Reviewer #2, Comment 2:

The novelty and advantages of the proposed mobility-state- and handover-direction-aware framework should be made more distinct from a number of existing ML/DRL-based handover optimization studies.

**Author response:** The revised manuscript now makes the novelty more explicit. The related-work discussion distinguishes the proposed framework from recent learning-based and HetNet mobility studies. The research gap and main contributions now emphasize that mobility state and handover direction are explicit controller inputs rather than implicit by-products of signal state, and that all four controllers preserve the same Event A3/FSM/RLM framework. A normalized relative KPI comparison with recent ML/DRL-based handover-optimization studies has also been added for contextual benchmarking.

**Location in Manuscript with Track Changes:** Related-work discussion: p. 3; research gap and contributions: pp. 3-4; Figure 15 and state-of-the-art contextual comparison: p. 29; conclusion: pp. 32-33.

## Reviewer #2, Comment 3:

There is a lack of description of the DQN model. Network Architecture, hyper parameters, replay memory, batch size, and convergence criteria should be included.

**Author response:** The revised DQN subsection now reports the input state variables, two fully connected ReLU hidden layers with 32 neurons each, 35 HOM–TTT output actions, 2,467 trainable parameters, learning rate 8×10^−4^, discount factor 0.97, replay-buffer size 20,000, mini-batch size 32, target-network update period of 8 episodes, epsilon decay from 1.0 to 0.05, and 300 training episodes. The convergence assessment is now described using the moving-average training reward and stabilization of KPI trends near the end of training. Appendix-A further specifies the DQN setup, training loop, replay-memory update, terminal-target handling, and target-network synchronization.

**Location in Manuscript with Track Changes:** DQN architecture and Table 3 hyperparameters: p. 17; DQN architecture, parameter count, replay memory, target network, and convergence assessment: pp. 17-18; Figure 7 DQN workflow: pp. 18-19; DQN pseudocode in Appendix A: p. 38.

## Reviewer #2, Comment 4:

The reward function formulation needs to be better reasoned, with an explanation of the choice of the weights for the KPIs.

**Author response:** The reward-function section has been revised to explain both the Q-learning reward and the DQN extension. The reward weights are now justified by physical severity: RLF and HOF receive the largest penalties because they interrupt service continuity; outage is penalized next; ping-pong is penalized to avoid instability; and latency/excessive handover receive smaller penalties to prevent an overly conservative policy. The DQN reward additionally includes smoothness and direction-consistency terms to reduce HOM/TTT oscillations and encourage direction-aware decisions.

**Location in Manuscript with Track Changes:** Reward formulation and weight rationale: pp. 14-15; DQN reward extension: p. 15; sensitivity limitation regarding reward weights: p. 15.

## Reviewer #2, Comment 5:

No statistical validation of the manuscript. Report multiple simulation runs, standard deviations and confidence intervals.

**Author response:** This has been addressed by adding direct numerical uncertainty reporting. Each controller is evaluated over three independent seeds, and the manuscript now reports seed-level values, mean, SD, SE, and t-based 95% confidence intervals for each policy-level KPI. Figure 14 also presents the 95% CI error bars. The text explicitly states that these intervals are descriptive and that overlapping intervals should not be interpreted as formal evidence of statistical superiority.

**Location in Manuscript with Track Changes:** Statistical definitions: p. 14; Table 4 and Tables 4a-4h with seed-level values, SD, SE, and 95% CI: pp. 25-26; Figure 14 with 95% CI error bars and cautious interpretation: pp. 27-28; conclusion with cautious statistical wording: pp. 32-33.

## Reviewer #2, Comment 6:

It is recommended to perform a more detailed comparison to some of the recent and state of the art handover optimization methods.

**Author response:** The revised manuscript expands the comparison with recent ML/DRL-based handover-optimization literature. It adds a related-work distinction in the Introduction and includes a normalized relative KPI gain/reduction comparison in Figure 15. The comparison is presented as trend-based contextual benchmarking because the cited studies differ in topology, traffic, propagation assumptions, and KPI definitions.

**Location in Manuscript with Track Changes:** Related-work comparison: p. 3; Figure 15 and its explanation: p. 29; recent references [49]-[55]: pp. 40-41.

## Reviewer #2, Comment 7:

The study is computer-based, solely. The authors should consider discussing real world applicability and challenges to deployment.

**Author response:** The revised manuscript now includes a real-world applicability, limitations, and reproducibility subsection. It explains that the framework keeps Event A3, HOM, TTT, FSM, and RLM concepts intact, so the analytical controller could be implemented as a lightweight self-optimization rule and the learning-based policies could be trained offline and deployed as lookup or inference modules in a centralized SON/O-RAN-style controller. The text also identifies deployment challenges, including multi-UE traffic, scheduler interactions, load-dependent interference, measurement-report delay, and vendor-specific RRC parameter constraints.

**Location in Manuscript with Track Changes:** Real-world applicability and deployment challenges: pp. 31-32; limitations and future work in conclusion: p. 33.

## Reviewer #2, Comment 8:

The computational complexity and running time of both Q-learning and DQN approaches should be analysed.

**Author response:** The revised manuscript adds a computational-complexity and implementation-cost discussion. It reports that Q-learning has an online decision cost of O(|A|) and memory cost of O(|S||A|), while DQN inference requires one forward pass through a 7-32-32-35 network with 2,467 trainable parameters and approximately 2,368 multiply-accumulate operations per forward pass. No wall-clock time was inserted because no hardware/runtime log was retained; the manuscript states this transparently rather than inventing unverifiable timing values.

**Location in Manuscript with Track Changes:** Computational complexity and implementation cost: p. 19.

## Reviewer #2, Comment 9:

Simulation settings, simulation code or supplementary materials should be made available to enhance data/ code availability/reproducibility.

**Author response:** The revised manuscript provides the simulation settings, seed-level KPI values, statistical summaries, controller state variables, action sets, reward expressions, DQN hyperparameters, and implementation-level pseudocode. The Data Availability Statement has been updated to state that all numerical data supporting the findings are provided within the manuscript and supporting information, including the KPI values, seed-level values, mean, SD, SE, and 95% confidence intervals. Appendix-A provides detailed pseudocode sufficient to understand, verify, and independently re-implement the simulation workflow.

**Location in Manuscript with Track Changes:** Simulation settings: pp. 13-14; state/action/reward/DQN settings: pp. 14-18; reproducibility discussion: pp. 31-32; Data Availability: p. 34; Appendix A implementation-level pseudocode: pp. 35-38.

## Reviewer #2, Comment 10:

There are some grammatical and language problems in the manuscript which need to be carefully proofread and professionally edited.

**Author response:** The manuscript has been proofread and revised for grammar, clarity, and consistency while preserving the technical meaning. The Introduction, Research Gap, DQN description, Results discussion, Figure 15 interpretation, real-world limitations, Data Availability Statement, and Conclusion are revised for clearer wording.

**Location in Manuscript with Track Changes:** Corrected introduction wording: p. 2; Figure 10 wording: p. 22; DQN workflow wording: pp. 18-19; Figure 15 caption/discussion: p. 29; revised conclusion: pp. 32-33.

## Reviewer #2, Comment 11:

Paper would benefit from an ablation study that would show the individual contribution of awareness of the mobility state and awareness of the handover direction.

**Author response:** The revised manuscript adds a mechanism-level ablation and sensitivity interpretation. It explains the expected effect of removing mobility-state awareness, removing handover-direction awareness, removing both awareness terms, and removing the DQN smoothness terms. The manuscript is explicit that this is not a separately retrained numerical ablation study and identifies quantitative component-removal retraining as future work.

**Location in Manuscript with Track Changes:** Ablation and sensitivity interpretation: pp. 20-21; future-work wording in conclusion: p. 33.

## Reviewer #2, Comment 12:

The conclusions presented are generally supported by the results presented; however, there are still some details and statistics that can be included to solidify the conclusions for publication.

**Author response:** The Conclusion is revised to include a more cautious interpretation of the statistical results. It now states that the 95% confidence intervals show overlap for several close comparisons and that the conclusions are based on the consistent multi-KPI pattern rather than formal significance testing. It also avoids overclaiming Q-learning performance, states that Q-learning improves over fixed A3 on the main descriptive mobility KPIs, and identifies future work on multi-UE load-dependent interference, additional random seeds, quantitative component-removal ablation, and reproducibility-supported implementation.

**Location in Manuscript with Track Changes:** Revised conclusion with numerical improvements and cautious statistical interpretation: pp. 32-33; supporting statistical tables: pp. 25-26; Figure 14 uncertainty interpretation: pp. 27-28.

We again thank the Academic Editor and reviewers for their constructive guidance. We believe that the revised manuscript is clearer, more reproducible, more statistically transparent, and more cautious in its conclusions.

Sincerely,

The Authors
