# Supplementary material for: Mobility-state- and handover-direction-aware analytical and machine learning-based HCP optimization in 5G heterogeneous networks
Source: PLoS One. 2026 Nov 12;21(11):e0362310. doi: 10.1371/journal.pone.0362310 (PMC13759111; doi:10.1371/journal.pone.0362310)
Supplement: Supplementary file 3 [file pone.0362310.s004.docx]

Response to Reviewers

**Mobility-State- and Handover-Direction-Aware Analytical and Machine Learning-Based HCP Optimization in 5G Heterogeneous Networks**

**Dear Academic Editor and Reviewers,**

Thank you for the constructive assessment and for recognizing the improvements made to the manuscript. We have carefully addressed all comments. The marked-up manuscript shows additions in blue and deletions in red, and the clean manuscript contains the accepted revised content without revision markup. All page references below refer to the Revised Manuscript with Track Changes PDF.

# Files supplied with this second revision

| **Submission label** | **File content** |
| --- | --- |
| Response to Reviewers | This verified point-by-point letter with locations in the final tracked manuscript. |
| Revised Manuscript with Track Changes | Marked-up revised manuscript; additions are blue and deletions are red. |
| Manuscript | Clean revised manuscript containing the accepted final content without markup. |

# Point-by-point response to Reviewer #2

**Reviewer Comment 1. *The novelty of the mobility state and handover direction should be highlighted more clearly compared to the recent DRL based on handover optimization studies. A table summarizing the differences with the latest literature would be helpful to read.***

**Response:** We thank the reviewer for this helpful suggestion. A new literature-comparison table now compares the present framework with recent learning-based handover-optimization studies in terms of the learning/control approach, principal state inputs, explicit mobility-state representation, explicit handover-direction representation, and the main distinction from the present work.

The paragraph immediately following the table clarifies that the novelty is not the use of DQN alone. The main contribution is the explicit combination of mobility state with macro-to-small, small-to-macro, and same-tier handover direction while retaining the standard Event A3, finite-state handover, and radio-link-monitoring procedures.

**Location in the revised manuscript:** Tracked manuscript p. 4, Table 1 and the paragraph immediately following it.

**Reviewer Comment 2. *The 3GPP specifications are used in the simulation environment, however, the discussion on the complexity of deployment in real-time 5G networks, as well as computational burden of the proposed DQN controller, should be extended.***

**Response:** We thank the reviewer for this important suggestion. The manuscript now includes a dedicated subsection entitled “Computational complexity and implementation cost.” It reports the online decision and memory costs of Q-learning and the inference footprint of the 7-32-32-35 DQN. The network contains 2,467 trainable parameters, requires approximately 2,368 multiply-accumulate operations per decision, and occupies approximately 9.6 kB for one float32 online network. The training-only replay buffer requires approximately 1.3-1.5 MB under the reported transition representation.

The deployment discussion now covers state collection and normalization, inference within the mobility-control cycle, operator-approved HOM-TTT mapping, action guardrails, shadow-mode evaluation, rollback criteria, telemetry delay, synchronization, signalling overhead, simultaneous UE requests, vendor-specific RRC constraints, and fallback to fixed A3.

**Location in the revised manuscript:** Tracked manuscript p. 25, subsection “Computational complexity and implementation cost.”

**Reviewer Comment 3. *The manuscript assesses a number of mobility KPIs. Adding to the reported performance increases, a statistical significance analysis (or a hypothesis test if appropriate) would add value.***

We thank the reviewer for this important comment. The statistical methodology has been clarified and expanded. Each controller is evaluated using three independent random seeds, and the manuscript reports the seed-level values, mean, standard deviation, standard error, and t-based 95% confidence interval for each policy-level KPI.

A formal pairwise hypothesis test was considered but was not applied because only three independent observations are available per controller. With such a small sample, the normality and equal-variance assumptions cannot be assessed reliably, and the resulting test would have very low statistical power. To avoid presenting potentially misleading p-values, the revised manuscript uses the confidence intervals as descriptive uncertainty measures and explicitly distinguishes non-overlapping intervals from overlapping intervals.

**Location in the revised manuscript:** Tracked manuscript p. 15, “Simulation Methodology”

**Reviewer Comment 4. *The reward function is well designed, but additional discussion about sensitivity in reward weights and the effect of reward weights on the convergence of learning would enhance the reproducibility.***

**Response:** We thank the reviewer for this valuable suggestion. We have expanded the reward-function discussion to explain the role of all reward-weight groups and the expected effects of setting the weights too small or too large. The new sensitivity analysis covers the severe service-continuity penalties for HOF, RLF, and outage; the ping-pong and excessive-handover penalties; the throughput, successful-handover, and latency terms; the HOM-TTT action-smoothing terms; and the handover-direction-consistency term.

The accompanying convergence discussion explains how poorly balanced weights can increase reward variance, produce over-conservative or overly aggressive policies, cause HOM-TTT action chattering, weaken the use of direction information, or slow adaptation under rapidly changing mobility conditions. The retained archive does not contain independent one-factor-at-a-time weight-perturbation runs. Therefore, Table 4 is explicitly presented as a qualitative, mechanism-based sensitivity analysis rather than as an unreported numerical experiment

**Location in the revised manuscript:** Tracked manuscript pp. 16-17, Eqs. (57)-(58), the expanded reward-weight and convergence discussion, and Table 4.

**Reviewer Comment 5. *The comparison is only with simulated environments. A discussion about possible validation by real network traces or hardware test bed validation would be more useful to show practical applicability.***

**Response:** We thank the reviewer for this valuable recommendation. Simulation cannot fully capture real measurement noise, protocol-processing delay, signalling overhead, hardware constraints and implementation latency. We have therefore expanded the manuscript to discuss real-network-trace replay and hardware-testbed validation and to relate our results cautiously to relevant experimental studies.

Akdogan et al. implemented the FLeXHO framework on an OpenAirInterface-based end-to-end testbed and reported a 94.5% handover-success rate. The proposed DQN achieves a mean handover-success probability of 95.0%, showing a similar reliability trend. However, we do not treat these values as directly comparable because the two studies differ in topology, traffic, mobility, controller design and KPI definitions. Our manuscript reports the DQN result within a common Event A3/FSM/RLM simulation framework.

We also discuss the OpenAirInterface SDR testbed of Bertolini and Maman, which implemented end-to-end X2 handover using USRP devices and a channel emulator. Their work demonstrates that measurement-based handover, TTT operation, throughput and handover-timing components can be evaluated in a full-stack hardware environment.

The revised manuscript proposes two future validation stages. First, recorded RSRP, RSRQ, SINR, UE mobility, measurement reports, handover outcomes, ping-pong and RLF traces would replace the simulated measurement stream, allowing all four controllers to be evaluated on identical real traces. Second, the controllers could be deployed on an OpenAirInterface/Open5GS or equivalent SDR testbed with multiple cells, a mobile or channel-emulated UE and fixed-Event-A3 fallback. The evaluation would measure handover success, interruption time, packet loss, throughput, HOF, RLF, ping-pong, decision latency, signalling overhead and computational cost.

We explicitly state that no real-trace or hardware validation has yet been conducted. Therefore, the current results remain simulation-based, and the experimental studies are used only to demonstrate the feasibility of a future validation pathway rather than to claim direct experimental verification.

**Location in the revised manuscript:** Tracked manuscript pp. 36-37, subsection “Real-world applicability, limitations, and reproducibility.”

**Reviewer Comment 6. *Enlarged some figures that include the system workflow, controller architecture, and simulation topology for better readability, especially for print out.***

**Response:** We thank the reviewer for this helpful presentation-related suggestion. The figures most directly related to the reviewer’s concern have been enlarged to improve readability of the simulation topology, system workflow, and learning-controller architecture, especially in printed form. Figure 1, which presents the simulation topology, was increased from 0.74 to 0.92 of the line width. The analytical-controller, Q-learning-controller, and DQN-controller diagrams in Figures 5, 6, and 7 were replaced with larger page-optimized versions so that their processing steps, decision branches, state/action flow, replay-memory operations, and target-network updates are easier to read.

Only the presentation size and layout were changed; the underlying controller methodology, sequence, labels, captions, numbering, and logic remain unchanged. In the marked-up manuscript, the earlier controller diagrams are shown as red deleted material and the enlarged final versions are shown as blue added material.

**Location in the revised manuscript:** Tracked manuscript: Fig. 1 on p. 6; Fig. 5 old and enlarged versions on pp. 13-14; Fig. 6 old and enlarged versions on pp. 19-20; and Fig. 7 old and enlarged versions on pp. 23-24. The related explanatory text appears on pp. 13, 18, and 22.

**Reviewer Comment 7. *The scalability to future 6G ultra-dense deployments, multi-user and dynamic traffic conditions should be explicitly discussed in the limitations section.***

**Response:** We thank the reviewer for this important suggestion. The limitations section now explains that the single-UE assumption does not fully represent load-dependent interference, inter-cell scheduling, admission control, queue dynamics, heterogeneous service classes, or resource competition. It identifies serving/target load, queue occupancy, allocated bandwidth, traffic class, fairness indicators, and conflict resolution as requirements for a multi-user extension.

The 6G discussion addresses per-UE inference growth, centralized telemetry and signalling bottlenecks, ultra-dense cells, high-frequency blockage, beam switching, multi-connectivity, rapidly changing traffic, batched inference, hierarchical control, parameter sharing, edge execution, and multi-agent coordination.

**Location in the revised manuscript:** Tracked manuscript p. 37, the multi-user limitation and 6G ultra-dense scalability paragraphs.

**Reviewer Comment 8. *The conclusion could be briefly a direction of future research, such as online learning, federated reinforcement learning or multi-agent handover optimization.***

**Response:** We thank the reviewer for this constructive suggestion. The final paragraph of the Conclusion now identifies real-trace and hardware-testbed validation, multi-UE dynamic traffic, additional random seeds, quantitative component-removal ablation, online or continual learning, federated reinforcement learning, and multi-agent reinforcement learning as future directions.

The paragraph also retains safety constraints, fixed-A3 fallback, and explicit evaluation of control latency and signalling overhead as requirements for practical deployment.

**Location in the revised manuscript:** Tracked manuscript p. 38, final paragraph of the Conclusion.

**Closing statement**

We appreciate the reviewers' careful and constructive suggestions. We believe that the revised manuscript is clearer regarding novelty, computational cost, statistical interpretation, reward sensitivity, practical validation, figure readability, scalability, limitations, and future work directions.

Sincerely,
The Authors
