# Supplementary material for: Mobility-state- and handover-direction-aware analytical and machine learning-based HCP optimization in 5G heterogeneous networks
Source: PLoS One. 2026 Nov 12;21(11):e0362310. doi: 10.1371/journal.pone.0362310 (PMC13759111; doi:10.1371/journal.pone.0362310)
Supplement: Supplementary file 4 [file pone.0362310.s005.docx]

# Response to Reviewers

### Manuscript: Mobility-State- and Handover-Direction-Aware Analytical and Machine Learning-Based HCP Optimization in 5G Heterogeneous Networks

**Dear Academic Editor and Reviewer #2,**

# Thank you for the constructive assessment and for recognizing the improvements made to the manuscript. We have carefully addressed all comments. The marked-up manuscript shows newly added or revised text in blue, while the clean manuscript contains the final revised content without revision markup. All page references in this response refer to the Revised Manuscript with Track Changes PDF.

# Files supplied with this revision

| **Submission label** | **File content** |
| --- | --- |
| Response to Reviewers | This point-by-point response letter, including the locations of the corresponding revisions in the final tracked manuscript. |
| Revised Manuscript with Track Changes | Marked-up revised manuscript showing newly added or revised text in blue. |
| Manuscript | Clean revised manuscript containing the final revised content without revision markup. |

## Reviewer #2 - Comment 1

**Reviewer comment:** There are only three independent seeds used. Three runs is a small number to make any claim to robustness of the claimed improvement, especially for a stochastic reinforcement-learning method, and the manuscript rightly calls the confidence intervals descriptive. The number of independent evaluation runs should be increased and/or a better reason should be given for restricting the analysis to the three seeds.

**Response:** We agree that 3 runs is not enough to make a powered inferential comparison. The manuscript now reports the confidence intervals descriptively, omits inferential claims of superiority, and calls for a larger prespecified multi-seed evaluation to be required for future confirmatory analysis.

***Changes addressed in revised manuscript: Abstract (p. 1); Simulation Methodology (p. 15); Limitations and Conclusion (p. 34).***

## Reviewer #2 - Comment 2

**Reviewer comment:** The seed level values shown in Table 6 are rather regular and symmetrical around the reported averages. Many of the metrics also exhibit very similar standard deviations between the various controllers. The authors should explain how these seed-level results were obtained, and ensure that they are actual simulation outputs, and not combined results. A solution to this concern would be to give the full raw output from each seed, velocity and controller, along with every KPI.

**Response:** Thank you for raising this concern. Table 6 now reports the retained Seed 1–3 values for all four controllers and eight KPIs together with their mean, SD, SE, and descriptive 95% confidence intervals. To improve transparency and facilitate direct verification, these Table 6 source values are also provided as a machine-readable CSV Supporting Information file. The complete per-velocity, per-seed raw simulation outputs were not retained and therefore were not retrospectively reconstructed.

***Changes addressed in revised manuscript: Results - Table 6 (pp. 28-29), including the seed-level provenance wording on p. 29.***

## Reviewer #2 - Comment 3

**Reviewer comment:** Training and evaluation protocol needs to be further clarified. The authors should explicitly inform the reader whether the training and testing were performed with independent and random seed, trajectory, channel realization, and/or exploration settings. It should also include details on preventing information leakage between training and evaluation.

**Response:** It is now clearly distinguished between training and evaluation. Following training, the policy is fixed and future evaluation is done by taking greedy actions without updating the Q-table/network. Original seed identifiers and random stream mapping are not stored, and were not reconstructed.

***Changes addressed in revised manuscript: Simulation Methodology - training/evaluation separation (p. 15).***

## Reviewer #2 - Comment 4

**Reviewer comment:** There are several implementation details that are not clearly specified, such as the simulation duration in number of steps for the numerical simulations, the length of the episodes, exact boundaries of the state bins for the Q-learning, the normalization range of all the DQN inputs, the definition of the terminal state, the parameters for the shadowing, optimizer parameters, and the full epsilon-decay procedure. These details would be needed for independent reproduction.

**Response:** All the implementable details (timing, number of episodes, Q-learning/DQN hyperparameters, state dimensions, architecture, replay details, epsilon schedule, definition of terminal state) have been reported in the manuscript. Details which could not be verified were not guessed or built up.

***Changes addressed in revised manuscript: Simulation Methodology (pp. 15); Machine Learning-Based HCP Optimization (pp. 15-16); Appendix A - Controller Implementation Pseudocode (pp. 36-39).***

## Reviewer #2 - Comment 5

**Reviewer comment:** The reward is structured to make both learning controllers better than the fixed A3 reward, which could imply an outcome-directed reward. The authors should explain the reward selection and hyper parameter tuning process, including if the reported evaluated scenarios were part of the process. It is worth to note the constraints that stem from the lack of quantitative reward-weight sensitivity experiments.

**Response:** The reward now is expressed in terms of mobility goals, rather than ranking controllers. The verified base reward weights are retained, smoothness/direction terms are interpreted as policy preferences, and reward sensitivity is only qualitative.

***Changes addressed in revised manuscript: Machine Learning-Based HCP Optimization - State representation, action space, and reward (pp. 16); reward-sensitivity discussion/Table 4 (p. 17).***

## Reviewer #2 - Comment 6

**Reviewer comment:** The Data Availability Statement indicates that all relevant data are included in the manuscript and supporting information. Not all underlying data is aggregated or pseudocoded, however. The authors are requested to make the per-run numerical outputs, the trained model parameters or Q-tables, and the configuration files available in a public repository, as well as the simulation code if possible. If it is not possible to share the code this should be clearly and explicitly stated with a clear reason.

**Response:** Thank you for highlighting this important reproducibility issue. All numerical values currently retained from the original study record and used directly in the manuscript tables and figures are reported in the manuscript and Supporting Information. In particular, Table 6 presents the retained seed-level numerical values together with their descriptive summaries and uncertainty measures, and these values are additionally supplied in a machine-readable CSV file for direct inspection and reuse.

The complete simulation source code is not publicly deposited because it forms part of an actively developed project implementation and is not maintained as a stand-alone released software package. Nevertheless, the revised manuscript provides the mathematical model, documented simulation parameters, KPI definitions, controller states and actions, reward formulations, training and evaluation procedures, controller hyperparameters, and implementation-level pseudocode for all four controllers in Appendix A.

***Changes addressed in revised manuscript: Reproducibility/limitations statement (p. 34); Data Availability (p. 35); Appendix A implementation pseudocode (pp. 36-39).***

## Reviewer #2 - Comment 7

**Reviewer comment:** Claims that DQN is the “best” or “strongest” should be qualified due to the evidence only being collected from three runs and no adequately powered inferential comparison. Conclusions should mention the best observed mean performance for the tested simulation conditions.

**Response:** Unqualified uses of statistically superior, best, and strongest have been removed or qualified. The manuscript now uses wording such as best observed mean performance under the tested conditions and does not provide an inferential claim of superiority.

***Changes addressed in revised manuscript: Abstract (p. 1); Convergence of Q-learning and DQN (p. 23); velocity-based results and Table 6 (pp. 28-29); Figure 14 discussion (p. 30); Conclusion (p. 34).***
