# Supplementary material for: Mobility-state- and handover-direction-aware analytical and machine learning-based HCP optimization in 5G heterogeneous networks
Source: PLoS One. 2026 Nov 12;21(11):e0362310. doi: 10.1371/journal.pone.0362310 (PMC13759111; doi:10.1371/journal.pone.0362310)
Supplement: Peer review history

# Peer review: Decision Letter 0

**DOI:** 10.1371/journal.pone.0362310.r001

**Authors:** Dinesh Nishad

5 Jun 2026

Dear Dr. Ameer,

Please submit your revised manuscript by Aug 04 2026 11:59PM. If you will need more time than this to complete your revisions, please reply to this message or contact the journal office at plosone@plos.org. When you're ready to submit your revision, log on to https://www.editorialmanager.com/pone/ and select the 'Submissions Needing Revision' folder to locate your manuscript file.

- A letter that responds to each point raised by the academic editor and reviewer(s). You should upload this letter as a separate file labeled 'Response to Reviewers'.
- A marked-up copy of your manuscript that highlights changes made to the original version. You should upload this as a separate file labeled 'Revised Manuscript with Track Changes'.
- An unmarked version of your revised paper without tracked changes. You should upload this as a separate file labeled 'Manuscript'.

If you would like to make changes to your financial disclosure, please include your updated statement in your cover letter. Guidelines for resubmitting your figure files are available below the reviewer comments at the end of this letter.

If applicable, we recommend that you deposit your laboratory protocols in protocols.io to enhance the reproducibility of your results. Protocols.io assigns your protocol its own identifier (DOI) so that it can be cited independently in the future. For instructions see: https://journals.plos.org/plosone/s/submission-guidelines#loc-laboratory-protocols. Additionally, PLOS ONE offers an option for publishing peer-reviewed Lab Protocol articles, which describe protocols hosted on protocols.io. Read more information on sharing protocols at https://plos.org/protocols?utm_medium=editorial-email&utm_source=authorletters&utm_campaign=protocols.

As the corresponding author, your ORCID iD is verified in the submission system and will appear in the published article. PLOS supports the use of ORCID, and we encourage all coauthors to register for an ORCID iD and use it as well. Please encourage your coauthors to verify their ORCID iD within the submission system before final acceptance, as unverified ORCID iDs will not appear in the published article. *Only*  the individual author can complete the verification step; PLOS staff *cannot*  verify ORCID iDs on behalf of authors.

We look forward to receiving your revised manuscript.

Kind regards,

Dinesh Kumar Nishad, PhD

Academic Editor

PLOS One

Journal Requirements:

When submitting your revision, we need you to address these additional requirements.

1. Please ensure that your manuscript meets PLOS ONE's style requirements, including those for file naming. The PLOS ONE style templates can be found at

https://journals.plos.org/plosone/s/file?id=wjVg/PLOSOne_formatting_sample_main_body.pdf and

https://journals.plos.org/plosone/s/file?id=ba62/PLOSOne_formatting_sample_title_authors_affiliations.pdf

2. Please note that PLOS One has specific guidelines on code sharing for submissions in which author-generated code underpins the findings in the manuscript. In these cases, we expect all author-generated code to be made available without restrictions upon publication of the work. Please review our guidelines at https://journals.plos.org/plosone/s/materials-and-software-sharing#loc-sharing-code and ensure that your code is shared in a way that follows best practice and facilitates reproducibility and reuse.

3. Thank you for stating the following in the Acknowledgments Section of your manuscript:

“The authors acknowledge the support of The Pennsylvania State University for providing open access funding under an institutional open access agreement.”

We note that you have provided funding information that is not currently declared in your Funding Statement. However, funding information should not appear in the Acknowledgments section or other areas of your manuscript. We will only publish funding information present in the Funding Statement section of the online submission form.

Please remove any funding-related text from the manuscript and let us know how you would like to update your Funding Statement. Currently, your Funding Statement reads as follows:

“The author(s) received no specific funding for this work.”

Please include your amended statements within your cover letter; we will change the online submission form on your behalf.

4. Thank you for stating the following financial disclosure:

“The authors acknowledge the support of The Pennsylvania State University for providing open access funding under an institutional open access agreement.”

At this time, please address the following queries:

a) Please clarify the sources of funding (financial or material support) for your study. List the grants or organizations that supported your study, including funding received from your institution.

b) State what role the funders took in the study. If the funders had no role in your study, please state: “The funders had no role in study design, data collection and analysis, decision to publish, or preparation of the manuscript.”

c) If any authors received a salary from any of your funders, please state which authors and which funders.

d) If you did not receive any funding for this study, please state: “The authors received no specific funding for this work.”

Please include your amended statements within your cover letter; we will change the online submission form on your behalf.

5. PLOS requires an ORCID iD for the corresponding author in Editorial Manager on papers submitted after December 6th, 2016. Please ensure that you have an ORCID iD and that it is validated in Editorial Manager. To do this, go to ‘Update my Information’ (in the upper left-hand corner of the main menu), and click on the Fetch/Validate link next to the ORCID field. This will take you to the ORCID site and allow you to create a new iD or authenticate a pre-existing iD in Editorial Manager.

6. Please ensure that you refer to Figures 1 - 7 in your text as, if accepted, production will need this reference to link the reader to the figure.

7. We note you have included a table to which you do not refer in the text of your manuscript. Please ensure that you refer to Tables 1 - 3 in your text; if accepted, production will need this reference to link the reader to the Table.

8. If the reviewer comments include a recommendation to cite specific previously published works, please review and evaluate these publications to determine whether they are relevant and should be cited. There is no requirement to cite these works unless the editor has indicated otherwise.

[Note: HTML markup is below. Please do not edit.]

Reviewers' comments:

Reviewer's Responses to Questions

**Comments to the Author**

1. Is the manuscript technically sound, and do the data support the conclusions?

Reviewer #1: Yes

Reviewer #2: Yes

**********

2. Has the statistical analysis been performed appropriately and rigorously?

Reviewer #1: Yes

Reviewer #2: No

**********

3. Have the authors made all data underlying the findings in their manuscript fully available?

The PLOS Data policy

Reviewer #1: Yes

Reviewer #2: No

**********

4. Is the manuscript presented in an intelligible fashion and written in standard English?

Reviewer #1: Yes

Reviewer #2: No

**********

Reviewer #1: 1. Is the manuscript technically sound, and do the data support the conclusions?

The manuscript is technically sound. The four controllers are evaluated under identical network geometry, propagation, and KPI definitions, ensuring fair comparison. The conclusions — that DQN delivers the best multi-KPI trade-off, Q-learning improves over fixed A3, and the analytical controller is an interpretable baseline — are supported by Table 4 and Figures 12–15. However, three concerns need addressing. First, only three random seeds are used without reporting variability measures; standard deviations or confidence intervals should be added to Table 4, particularly for closely performing methods. Second, the single-UE assumption excludes load-dependent interference effects and should be explicitly acknowledged as a limitation. Third, no ablation is provided for the DQN architecture choices; a brief sensitivity discussion on hidden layer size and training episodes is needed. Finally, the conclusion claiming Q-learning outperforms fixed A3 across all KPIs is inconsistent with Figure 12(c) and should be corrected.

2. Has the statistical analysis been performed appropriately and rigorously?

The statistical treatment is the main weakness. Results are reported as averages over three seeds with no variability measures, which is insufficient given stochastic shadowing and random mobility. Differences between closely performing methods — for example, latency of 0.128 s vs. 0.123 s between the analytical controller and Q-learning — may not be statistically significant. The authors should increase seeds and report standard deviations, or justify the current approach. Additionally, the eight reward weights in Equation 57 are stated without justification; the authors should clarify whether they were tuned systematically or empirically, and briefly discuss sensitivity to weight perturbations.

3. Have the authors made all data underlying the findings fully available?

The authors state all data are within the manuscript, which is acceptable for simulation-based work. However, the simulation code is not shared. Depositing the code in a public repository would substantially improve reproducibility, consistent with PLOS ONE policy. If code sharing is not feasible, a more detailed algorithmic description sufficient for independent replication — particularly for DQN training and the analytical controller parameter bounds — should be provided.

4. Is the manuscript presented in an intelligible fashion and written in standard English?

The manuscript is generally readable and well-structured. However, several issues require correction: (i) "small cells augment capacity and reuse" is an incomplete sentence; (ii) "Figure 10 shows The TTT trade-off" contains an erroneous capital; (iii) "it is more can handle larger" is grammatically incorrect; (iv) the caption of Figure 15 incorrectly describes the figure as showing training reward convergence when it actually presents normalized KPI gain comparisons with recent literature. A careful language revision is recommended. The authors should also include three references that better contextualize their work. Lv, Z., Singh, A.K. and Li, J., 2021. Deep learning for security problems in 5G heterogeneous networks. IEEE Network, 35(2), pp.67-73 — relevant to motivating learning-based management in 5G HetNets. Kiran, K., 2023. 5G heterogeneous network (HetNets): a self-optimization technique for vertical handover management. International Journal of Pervasive Computing and Communications, 19(1), pp.1-22 — directly related to vertical handover self-optimization and should be discussed in related work. Alhulayil, M. and López-Benítez, M., 2019, October. Methods for the allocation of almost blank subframes with fixed duty cycle for improved LTE-U/Wi-Fi coexistence. In 2019 International Conference on Wireless Networks and Mobile Communications (WINCOM) (pp. 1-6). IEEE — ABS coexistence mechanisms affect SINR and resource availability during handover in heterogeneous deployments and are relevant to the broader HetNet context of this work.

Reviewer #2: 1. The manuscript is of relevance and tackles a critical issue in 5G heterogeneous network mobility management.

2. The novelty and advantages of the proposed mobility-state- and handover-direction-aware framework should be made more distinct from a number of existing ML/DRL-based handover optimization studies.

There is a lack of description of the DQN model. Network Architecture, hyper parameters, replay memory, batch size, and convergence criteria should be included.

4. The reward function formulation needs to be better reasoned, with an explanation of the choice of the weights for the KPIs.

5. No statistical validation of the manuscript. Report multiple simulation runs, standard deviations and confidence intervals.

It is recommended to perform a more detailed comparison to some of the recent and state of the art handover optimization methods.

7. The study is computer-based, solely. The authors should consider discussing real world applicability and challenges to deployment.

The computational complexity and running time of both Q-learning and DQN approaches should be analysed.

9. Simulation settings, simulation code or supplementary materials should be made available to enhance data/ code availability/reproducibility.

10.There are some grammatical and language problems in the manuscript which need to be carefully proofread and professionally edited.

11. paper would benefit from an ablation study that would show the individual contribution of awareness of the mobility state and awareness of the handover direction.

12. The conclusions presented are generally supported by the results presented; however, there are still some details and statistics that can be included to solidify the conclusions for publication.

**********

what does this mean?). If published, this will include your full peer review and any attached files.

If you choose “no”, your identity will remain anonymous but your review may still be made public.

**Do you want your identity to be public for this peer review?**  For information about this choice, including consent withdrawal, please see our Privacy Policy

Reviewer #1: No

Reviewer #2: No

**********

[NOTE: If reviewer comments were submitted as an attachment file, they will be attached to this email and accessible via the submission site. Please log into your account, locate the manuscript record, and check for the action link "View Attachments". If this link does not appear, there are no attachment files.]

To ensure your figures meet our technical requirements, please review our figure guidelines: https://journals.plos.org/plosone/s/figures

You may also use PLOS’s free figure tool, NAAS, to help you prepare publication quality figures: https://journals.plos.org/plosone/s/figures#loc-tools-for-figure-preparation.

NAAS will assess whether your figures meet our technical requirements by comparing each figure against our figure specifications.

# Author comment: Author response to Decision Letter 1

**DOI:** 10.1371/journal.pone.0362310.r002

8 Jul 2026

We uploaded a document containing a response to the reviewer's comments.

Attachment Submitted filename: Response_to_Reviewers.docx

# Peer review: Decision Letter 1

**DOI:** 10.1371/journal.pone.0362310.r003

**Authors:** Dinesh Nishad

17 Jul 2026

Dear Dr. Ameer,

Thank you for submitting your manuscript to PLOS ONE. After careful consideration, we feel that it has merit but does not fully meet PLOS ONE’s publication criteria as it currently stands. Therefore, we invite you to submit a revised version of the manuscript that addresses the points raised during the review process.

Please submit your revised manuscript by Aug 16 2026 11:59PM. If you will need more time than this to complete your revisions, please reply to this message or contact the journal office at plosone@plos.org. When you're ready to submit your revision, log on to https://www.editorialmanager.com/pone/ and select the 'Submissions Needing Revision' folder to locate your manuscript file.

- A letter that responds to each point raised by the academic editor and reviewer(s). You should upload this letter as a separate file labeled 'Response to Reviewers'.
- A marked-up copy of your manuscript that highlights changes made to the original version. You should upload this as a separate file labeled 'Revised Manuscript with Track Changes'.
- An unmarked version of your revised paper without tracked changes. You should upload this as a separate file labeled 'Manuscript'.

If you would like to make changes to your financial disclosure, please include your updated statement in your cover letter. Guidelines for resubmitting your figure files are available below the reviewer comments at the end of this letter.

If applicable, we recommend that you deposit your laboratory protocols in protocols.io to enhance the reproducibility of your results. Protocols.io assigns your protocol its own identifier (DOI) so that it can be cited independently in the future. For instructions see: https://journals.plos.org/plosone/s/submission-guidelines#loc-laboratory-protocols. Additionally, PLOS ONE offers an option for publishing peer-reviewed Lab Protocol articles, which describe protocols hosted on protocols.io. Read more information on sharing protocols at https://plos.org/protocols?utm_medium=editorial-email&utm_source=authorletters&utm_campaign=protocols.

As the corresponding author, your ORCID iD is verified in the submission system and will appear in the published article. PLOS supports the use of ORCID, and we encourage all coauthors to register for an ORCID iD and use it as well. Please encourage your coauthors to verify their ORCID iD within the submission system before final acceptance, as unverified ORCID iDs will not appear in the published article. *Only*  the individual author can complete the verification step; PLOS staff *cannot*  verify ORCID iDs on behalf of authors.

We look forward to receiving your revised manuscript.

Kind regards,

Dinesh Kumar Nishad, PhD

Academic Editor

PLOS One

Journal Requirements:

1. If the reviewer comments include a recommendation to cite specific previously published works, please review and evaluate these publications to determine whether they are relevant and should be cited. There is no requirement to cite these works unless the editor has indicated otherwise.

2. Please review your reference list to ensure that it is complete and correct. If you have cited papers that have been retracted, please include the rationale for doing so in the manuscript text, or remove these references and replace them with relevant current references. Any changes to the reference list should be mentioned in the rebuttal letter that accompanies your revised manuscript. If you need to cite a retracted article, indicate the article’s retracted status in the References list and also include a citation and full reference for the retraction notice.

[Note: HTML markup is below. Please do not edit.]

Reviewers' comments:

Reviewer's Responses to Questions

**Comments to the Author**

Reviewer #1: All comments have been addressed

Reviewer #2: All comments have been addressed

**********

2. Is the manuscript technically sound, and do the data support the conclusions?

Reviewer #1: Yes

Reviewer #2: Yes

**********

3. Has the statistical analysis been performed appropriately and rigorously?

Reviewer #1: Yes

Reviewer #2: Yes

**********

4. Have the authors made all data underlying the findings in their manuscript fully available?

The PLOS Data policy

Reviewer #1: Yes

Reviewer #2: Yes

**********

5. Is the manuscript presented in an intelligible fashion and written in standard English?

Reviewer #1: Yes

Reviewer #2: Yes

**********

Reviewer #1: No further comments. In this version of the manuscript, the authors have addressed all my concerns and questions.

Reviewer #2: The manuscript provides a thorough research on optimizing the handover control parameters of 5G heterogeneous networks, from analytical and machine learning perspectives, based on the knowledge of the mobility state and the handover direction. The design of the comparison of the fixed Event A3, analytical adaptive control, Q-learning, and DQN is technically sound and in line with the standards. The updated manuscript has been greatly improved with respect to the coverage of literature, methodological clarity, details about the simulations, and statistical reporting. A few points, however, might enhance the manuscript before it is published.

1.The novelty of the mobility state and handover direction should be highlighted more clearly compared to the recent DRL based on handover optimization studies. A table summarizing the differences with the latest literature would be helpful to read.

2.The 3GPP specifications are used in the simulation environment, however, the discussion on the complexity of deployment in real-time 5G networks, as well as computational burden of the proposed DQN controller, should be extended.

3.The manuscript assesses a number of mobility KPIs. Adding to the reported performance increases, a statistical significance analysis (or a hypothesis test if appropriate) would add value.

4.The reward function is well designed, but additional discussion about sensitivity in reward weights and the effect of reward weights on the convergence of learning would enhance the reproducibility.

5.The comparison is only with simulated environments. A discussion about possible validation by real network traces or hardware test bed validation would be more useful to show practical applicability.

6.Enlarged some figures that include the system workflow, controller architecture, and simulation topology for better readability, especially for print out.

7.The scalability to future 6G ultra-dense deployments, multi-user and dynamic traffic conditions should be explicitly discussed in the limitations section.

8. The conclusion could be briefly a direction of future research, such as online learning, federated reinforcement learning or multi-agent handover optimization.

Overall, the manuscript is a valuable contribution to mobility optimization in 5G heterogeneous networks. The methodology is well developed and the experiments extensive, and the conclusions, although generally supported by the results presented, are generally valid. The rest of the suggestions are mostly about discussion, presentation or practical implications.

**********

what does this mean?). If published, this will include your full peer review and any attached files.

If you choose “no”, your identity will remain anonymous but your review may still be made public.

**Do you want your identity to be public for this peer review?**  For information about this choice, including consent withdrawal, please see our Privacy Policy

Reviewer #1: No

Reviewer #2: No

**********

[NOTE: If reviewer comments were submitted as an attachment file, they will be attached to this email and accessible via the submission site. Please log into your account, locate the manuscript record, and check for the action link "View Attachments". If this link does not appear, there are no attachment files.]

To ensure your figures meet our technical requirements, please review our figure guidelines: https://journals.plos.org/plosone/s/figures

You may also use PLOS’s free figure tool, NAAS, to help you prepare publication quality figures: https://journals.plos.org/plosone/s/figures#loc-tools-for-figure-preparation.

NAAS will assess whether your figures meet our technical requirements by comparing each figure against our figure specifications.

# Author comment: Author response to Decision Letter 2

**DOI:** 10.1371/journal.pone.0362310.r004

3 Aug 2026

We have uploaded a file.

Attachment Submitted filename: Response to Reviewers.docx

# Peer review: Decision Letter 2

**DOI:** 10.1371/journal.pone.0362310.r005

**Authors:** Dinesh Nishad

10 Sep 2026

Dear Dr. Ameer,

Thank you for submitting your manuscript to PLOS One. After careful consideration, we feel that it has merit but does not fully meet PLOS One’s publication criteria as it currently stands. Therefore, we invite you to submit a revised version of the manuscript that addresses the points raised during the review process.

Please submit your revised manuscript by Nov 09 2026 11:59PM. If you will need more time than this to complete your revisions, please reply to this message or contact the journal office at plosone@plos.org. When you're ready to submit your revision, log on to https://www.editorialmanager.com/pone/ and select the 'Submissions Needing Revision' folder to locate your manuscript file.

- A letter that responds to each point raised by the academic editor and reviewer(s). You should upload this letter as a separate file labeled 'Response to Reviewers'.
- A marked-up copy of your manuscript that highlights changes made to the original version. You should upload this as a separate file labeled 'Revised Manuscript with Track Changes'.
- An unmarked version of your revised paper without tracked changes. You should upload this as a separate file labeled 'Manuscript'.

If you would like to make changes to your financial disclosure, please include your updated statement in your cover letter. Guidelines for resubmitting your figure files are available below the reviewer comments at the end of this letter.

If applicable, we recommend that you deposit your laboratory protocols in protocols.io to enhance the reproducibility of your results. Protocols.io assigns your protocol its own identifier (DOI) so that it can be cited independently in the future. For instructions see: https://journals.plos.org/plosone/s/submission-guidelines#loc-laboratory-protocols. Additionally, PLOS One offers an option for publishing peer-reviewed Lab Protocol articles, which describe protocols hosted on protocols.io. Read more information on sharing protocols at https://plos.org/protocols?utm_medium=editorial-email&utm_source=authorletters&utm_campaign=protocols.

As the corresponding author, your ORCID iD is verified in the submission system and will appear in the published article. PLOS supports the use of ORCID, and we encourage all coauthors to register for an ORCID iD and use it as well. Please encourage your coauthors to verify their ORCID iD within the submission system before final acceptance, as unverified ORCID iDs will not appear in the published article. *Only*  the individual author can complete the verification step; PLOS staff *cannot*  verify ORCID iDs on behalf of authors.

We look forward to receiving your revised manuscript.

Kind regards,

Dinesh Kumar Nishad, PhD

Academic Editor

PLOS One

Journal Requirements:

If the reviewer comments include a recommendation to cite specific previously published works, please review and evaluate these publications to determine whether they are relevant and should be cited. There is no requirement to cite these works unless the editor has indicated otherwise.

[Note: HTML markup is below. Please do not edit.]

Reviewers' comments:

Reviewer's Responses to Questions

**Comments to the Author**

Reviewer #2: All comments have been addressed

**********

2. Is the manuscript technically sound, and do the data support the conclusions?

Reviewer #2: Partly

**********

3. Has the statistical analysis been performed appropriately and rigorously?

Reviewer #2: No

**********

4. Have the authors made all data underlying the findings in their manuscript fully available?

The PLOS Data policy

Reviewer #2: No

**********

5. Is the manuscript presented in an intelligible fashion and written in standard English?

Reviewer #2: Yes

**********

Reviewer #2: The revised manuscript is much improved. The novelty, computational demands, real-world deployment factors, reward-weight interpretation, restrictions, scalability, and future research directions have been emphasized by the authors. The larger figures and pseudocode at the lower level also make the code easier to read. Before the manuscript can be considered acceptable, however, the following points should be dealt with:

1.There are only three independent seeds used. Three runs is a small number to make any claim to robustness of the claimed improvement, especially for a stochastic reinforcement-learning method, and the manuscript rightly calls the confidence intervals descriptive. The number of independent evaluation runs should be increased and/or a better reason should be given for restricting the analysis to the three seeds.

2.The seed level values shown in Table 6 are rather regular and symmetrical around the reported averages. Many of the metrics also exhibit very similar standard deviations between the various controllers. The authors should explain how these seed-level results were obtained, and ensure that they are actual simulation outputs, and not combined results. A solution to this concern would be to give the full raw output from each seed, velocity and controller, along with every KPI.

3.Training and evaluation protocol needs to be further clarified. The authors should explicitly inform the reader whether the training and testing were performed with independent and random seed, trajectory, channel realization, and/or exploration settings. It should also include details on preventing information leakage between training and evaluation.

4.There are several implementation details that are not clearly specified, such as the simulation duration in number of steps for the numerical simulations, the length of the episodes, exact boundaries of the state bins for the Q-learning, the normalization range of all the DQN inputs, the definition of the terminal state, the parameters for the shadowing, optimizer parameters, and the full epsilon-decay procedure. These details would be needed for independent reproduction.

5.The reward is structured to make both learning controllers better than the fixed A3 reward, which could imply an outcome-directed reward. The authors should explain the reward selection and hyper parameter tuning process, including if the reported evaluated scenarios were part of the process. It is worth to note the constraints that stem from the lack of quantitative reward-weight sensitivity experiments.

6.The Data Availability Statement indicates that all relevant data are included in the manuscript and supporting information. Not all underlying data is aggregated or pseudocoded, however. The authors are requested to make the per-run numerical outputs, the trained model parameters or Q-tables, and the configuration files available in a public repository, as well as the simulation code if possible. If it is not possible to share the code this should be clearly and explicitly stated with a clear reason.

7.Claims that DQN is the “best” or “strongest” should be qualified due to the evidence only being collected from three runs and no adequately powered inferential comparison. Conclusions should mention the best observed mean performance for the tested simulation conditions.

The manuscript shows promise and most of the previous presentation related issues are addressed. However, more transparency with regard to the statistical findings, separation of training and evaluation, and underlying data are needed to meet the requirements of reproducability.

**********

what does this mean?). If published, this will include your full peer review and any attached files.

If you choose “no”, your identity will remain anonymous but your review may still be made public.

**Do you want your identity to be public for this peer review?**  For information about this choice, including consent withdrawal, please see our Privacy Policy

Reviewer #2: No

**********

[NOTE: If reviewer comments were submitted as an attachment file, they will be attached to this email and accessible via the submission site. Please log into your account, locate the manuscript record, and check for the action link "View Attachments". If this link does not appear, there are no attachment files.]

To ensure your figures meet our technical requirements, please review our figure guidelines: https://journals.plos.org/plosone/s/figures

You may also use PLOS’s free figure tool, NAAS, to help you prepare publication quality figures: https://journals.plos.org/plosone/s/figures#loc-tools-for-figure-preparation.

NAAS will assess whether your figures meet our technical requirements by comparing each figure against our figure specifications.

# Author comment: Author response to Decision Letter 3

**DOI:** 10.1371/journal.pone.0362310.r006

17 Sep 2026

We uploaded a file.

Attachment Submitted filename: Response_to_Reviewers_auresp_3.docx

# Peer review: Decision Letter 3

**DOI:** 10.1371/journal.pone.0362310.r007

**Authors:** Dinesh Nishad

22 Oct 2026

Mobility-State- and Handover-Direction-Aware Analytical and Machine Learning-Based HCP Optimization in 5G Heterogeneous Networks

PONE-D-26-22505R3

Dear Dr. Ameer,

We’re pleased to inform you that your manuscript has been judged scientifically suitable for publication and will be formally accepted for publication once it meets all outstanding technical requirements.

Within one week, you’ll receive an e-mail detailing the required amendments. When these have been addressed, you’ll receive a formal acceptance letter and your manuscript will be scheduled for publication.

An invoice will be generated when your article is formally accepted. Please note, if your institution has a publishing partnership with PLOS and your article meets the relevant criteria, all or part of your publication costs will be covered. Please make sure your user information is up-to-date by logging into Editorial Manager at Editorial Manager® and clicking the ‘Update My Information' link at the top of the page. For questions related to billing, please contact billing support.

If your institution or institutions have a press office, please notify them about your upcoming paper to help maximize its impact. If they’ll be preparing press materials, please inform our press team as soon as possible -- no later than 48 hours after receiving the formal acceptance. Your manuscript will remain under strict press embargo until 2 pm Eastern Time on the date of publication. For more information, please contact onepress@plos.org.

Kind regards,

Dinesh Kumar Nishad, PhD

Academic Editor

PLOS One

Additional Editor Comments (optional):

Reviewers' comments:

Reviewer's Responses to Questions

**Comments to the Author**

Reviewer #3: All comments have been addressed

**********

2. Is the manuscript technically sound, and do the data support the conclusions?

Reviewer #3: Yes

**********

3. Has the statistical analysis been performed appropriately and rigorously?

Reviewer #3: Yes

**********

4. Have the authors made all data underlying the findings in their manuscript fully available?

The PLOS Data policy

Reviewer #3: Yes

**********

5. Is the manuscript presented in an intelligible fashion and written in standard English?

Reviewer #3: Yes

**********

Reviewer #3: The revision improves methodological clarity, documents controller implementation, and appropriately limits performance claims to the tested simulation conditions. While the three-seed evaluation and incomplete retention of raw outputs remain limitations, these are explicitly acknowledged. I recommend acceptance for publication in PLOS ONE as an exploratory simulation study.

**********

what does this mean?). If published, this will include your full peer review and any attached files.

If you choose “no”, your identity will remain anonymous but your review may still be made public.

**Do you want your identity to be public for this peer review?**  For information about this choice, including consent withdrawal, please see our Privacy Policy

Reviewer #3: No

**********

# Editor report: Acceptance letter

**DOI:** 10.1371/journal.pone.0362310.r008

**Authors:** Dinesh Nishad

PONE-D-26-22505R3

PLOS One

Dear Dr. Ameer,

I'm pleased to inform you that your manuscript has been deemed suitable for publication in PLOS One. Congratulations! Your manuscript is now being handed over to our production team.

At this stage, our production department will prepare your paper for publication. This includes ensuring the following:

* All references, tables, and figures are properly cited

* All relevant supporting information is included in the manuscript submission,

* There are no issues that prevent the paper from being properly typeset

You will receive further instructions from the production team, including instructions on how to review your proof when it is ready. Please keep in mind that we are working through a large volume of accepted articles, so please give us a few days to review your paper and let you know the next and final steps.

Lastly, if your institution or institutions have a press office, please let them know about your upcoming paper now to help maximize its impact. If they'll be preparing press materials, please inform our press team within the next 48 hours. Your manuscript will remain under strict press embargo until 2 pm Eastern Time on the date of publication. For more information, please contact onepress@plos.org.

You will receive an invoice from PLOS for your publication fee after your manuscript has reached the completed accept phase. If you receive an email requesting payment before acceptance or for any other service, this may be a phishing scheme. Learn how to identify phishing emails and protect your accounts at https://explore.plos.org/phishing.

If we can help with anything else, please email us at customercare@plos.org.

Thank you for submitting your work to PLOS One and supporting open access.

Kind regards,

PLOS One Editorial Office Staff

on behalf of

Dr. Dinesh Kumar Nishad

Academic Editor

PLOS One
